# Looking forwards and backwards: dynamics and genealogies of locally regulated populations

**Published:** 2023-05-23

**Authors:** Alison M. Etheridge, Ian Letter, Terence Tsui Ho Lung, Thomas G. Kurtz, Peter L. Ralph

**Affiliations:** Department of Statistics, Oxford University, 24-29 St Giles, Oxford OX1 3LB, UK; Department of Statistics, Oxford University, 24-29 St Giles, Oxford OX1 3LB, UK; Department of Statistics, Oxford University, 24-29 St Giles, Oxford OX1 3LB, UK; Departments of Mathematics and Statistics, University of Wisconsin - Madison, 480 Lincoln Drive, Madison, WI 53706-1388, USA; Departments of Mathematics and Biology, University of Oregon, Fenton Hall, Eugene, OR 97403-1222, USA

**Keywords:** population model, interacting superprocess, lookdown construction, porous medium equation, reaction-diffusion equation, travelling waves, genealogies, Fisher-KPP equation, Primary, Secondary

## Abstract

We introduce a broad class of mechanistic spatial models to describe how spatially heterogeneous populations live, die, and reproduce. Individuals are represented by points of a point measure, whose birth and death rates can depend both on spatial position and local population density, defined at a location to be the convolution of the point measure with a suitable non-negative integrable kernel centred on that location. We pass to three different scaling limits: an interacting superprocess, a nonlocal partial differential equation (PDE), and a classical PDE. The classical PDE is obtained both by a two-step convergence argument, in which we first scale time and population size and pass to the nonlocal PDE, and then scale the kernel that determines local population density; and in the important special case in which the limit is a reaction-diffusion equation, directly by simultaneously scaling the kernel width, timescale and population size in our individual based model.

A novelty of our model is that we explicitly model a juvenile phase. The number of juveniles produced by an individual depends on local population density at the location of the parent; these juvenile offspring are thrown off in a (possibly heterogeneous, anisotropic) Gaussian distribution around the location of the parent; they then reach (instant) maturity with a probability that can depend on the local population density at the location at which they land. Although we only record mature individuals, a trace of this two-step description remains in our population models, resulting in novel limits in which the spatial dynamics are governed by a nonlinear diffusion.

Using a lookdown representation, we are able to retain information about genealogies relating individuals in our population and, in the case of deterministic limiting models, we use this to deduce the backwards in time motion of the ancestral lineage of an individual sampled from the population. We observe that knowing the history of the population density is not enough to determine the motion of ancestral lineages in our model. We also investigate (and contrast) the behaviour of lineages for three different deterministic models of a population expanding its range as a travelling wave: the Fisher-KPP equation, the Allen-Cahn equation, and a porous medium equation with logistic growth.

## 1 Introduction

As one takes a journey, long or short, the landscape changes: forests thicken or thin or change their composition; even in flat plains springtime grasslands host intergrading mosaics of different types of flowers. The aim of this paper is to introduce and study a broad class of mechanistic spatial models that might describe how spatially heterogeneous populations live, die, and reproduce. Questions that we (start to) address include: How does population density change across space and time? How might we learn about the underlying dynamics from genealogical or genetic data? And, how does genetic ancestry spread across geography when looking back through time in these populations?

Reproduction of individuals naturally leads to spatial branching process models, including branching random walk, branching Brownian motion, and the Dawson-Watanabe superprocesses. However, as a result of the branching assumption (once born, individuals behave independently of one another), a population evolving according to any of these models will either die out or grow without bound and, in so doing, can develop clumps of arbitrarily large density and extent. Our starting point here is an individual-based model of a single species in continuous space in which birth, death, and establishment may all depend on local population density as well as on spatial location, allowing for stable populations through density-dependent feedback.

Although it is often mathematically convenient to assume that individuals follow Brownian motion during their lifetime, in our model, offspring are thrown off according to some spatial distribution centred on the location of the parent and do not subsequently move. This is particularly appropriate for modelling plant populations, in which this dispersal of offspring around the parent is the only source of spatial motion.

Often models do not distinguish between juveniles and adults, so, for example, the number of adults produced by a single parent is determined only by the degree of crowding at the location of the parent. Although we shall similarly only follow the adult population, in formulating the dynamics of the models we shall distinguish between production of juveniles, which will depend upon the location of the adult, and their successful establishment, which will depend on the location in which a juvenile lands. The result is that not only the absolute number, but also the spatial distribution around their parent, of those offspring that survive to adulthood will depend upon the local population density.

We shall consider three different classes of scaling limits for our model. The first yields a class of (generalised) superprocesses in which coefficients governing both the spatial motion and the branching components of the process can depend on local population density; the second is a corresponding class of deterministic non-local differential equations; and the third are classical PDEs. We measure local population density around a point by convolving with a smooth kernel ρ(⋅), which may differ for the two stages of reproduction. When the limiting population process is deterministic, it is a (weak) solution of an equation of the form

(1.1) $$\partial_{t}\varphi_{t}\left(x\right)=r\left(x,\varphi_{t}\right){ℬ}^{*}\left[\varphi_{t}\left(\cdot \right)\gamma \left(\cdot ,\varphi_{t}\right)\right]\left(x\right)+\varphi_{t}\left(x\right)F\left(x,\varphi_{t}\right),$$

where $\varphi_{t}(x)$ can be thought of as the population density at x (although the limit may be a measure without a density), and ${ℬ}^{*}$ is (the adjoint of) a strictly uniformly elliptic second order differential operator, typically the Laplacian. The dependence of each of the terms r,γ, and F on φ is only through the local density at x, e.g., F(x,φ)=F(x,ρ*φ(x)). We shall be more specific about the parameters below.

By replacing ρ by $\rho^{\epsilon }(\cdot )=\rho (\cdot /\epsilon )/\epsilon^{d}$, we can also scale the “width” of the region over which we measure local population density. When the population follows (1.1), we expect that if we take a second limit of ϵ→0, thus scaling the kernels appearing in r,γ, and F and making interactions pointwise, we should recover a nonlinear PDE. We verify that this is indeed the case in two important examples: a special case of the porous medium equation with a logistic growth term, in which the limiting equation takes the form

(1.2) $$\partial_{t}\varphi =\Delta \left(\varphi^{2}\right)+\varphi (1-\varphi );$$

and a wide class of semi-linear PDEs of the form

(1.3) $$\partial_{t}\varphi ={ℬ}^{*}\varphi +\varphi F(\varphi ),$$

which includes the Fisher-KPP equation and the Allen-Cahn equation. Equations of this form have been studied extensively in the context of spatial ecology (see for instance Lam and Lou [2023] and Cantrell and Cosner [2004]) and in many other fields; for instance, Ghosh and Good [2022] derive a stochastic version of (1.3) to describe abundances of mutant bacteria strains along the human gut, while Li et al. [2022] study the effects of nonlinear diffusion on long-term survival of a lattice-based interacting particle system. However, we do not study the effect of movement of adults, which can additionally affect the limiting equations: see for instance Holmes et al. [1994] or Potts and Börger [2023].

It is of interest to understand under what conditions we can replace the two-step limiting process described above by one in which we simultaneously scale the kernels and the other parameters in our population model to arrive at the PDE limit. This is mathematically much more challenging, but we establish such one-step convergence in cases for which the limit is a classical reaction-diffusion equation of the form (1.3) with ℬ=Δ, and ρ is a Gaussian density. We allow a wide class of reaction terms, F, so that the Fisher-KPP equation (that is equation (1.3) with ℬ=Δ and F(φ)=1-φ) emerges as a special case.

Such results on (one-step) convergence to reaction-diffusion equation limits have been achieved for a variety of interacting particle systems. Following the now classical contributions of De Masi et al. [1986], DeMasi and Presutti [2006], Oelschläger [1985], much of this work has focused on lattice based models with one particle per site, or on systems with a fixed number, N, of interacting diffusions as N→∞. For systems of proliferating particles, as considered for example by Oelschläger [1989], Flandoli et al. [2019], Flandoli and Huang [2021], an additional challenge (also apparent in our models), is the control of concentration of particles. We follow Oelschläger [1989], Flandoli et al. [2019] in considering ‘moderate interactions’, meaning that the number of individuals in the neighbourhood over which we measure local population density tends to infinity, whereas Flandoli and Huang [2021] also consider the situation in which that number remains finite. We refer to Flandoli and Huang [2021] for a more thorough literature review, but note that both our model and scaling differ from those considered in the body of work discussed there: whereas in those settings, the only scalings are the number of particles in the system and the size of the neighbourhood over which individuals interact with one another, in keeping with the vast literature on continuous state branching models, we also scale time and so must ensure that births are adequately compensated by deaths to prevent the population from exploding.

The history of a natural population is often only accessible indirectly, through patterns of genetic diversity that have been laid down; from genetic data, one can try to infer the genealogical trees that relate individuals in a sample from the population, and these have been shaped by its history [see e.g., Neigel and Avise, 1993, Kelleher et al., 2019]. It is therefore of interest to establish information about the distribution of genealogical trees under our population model, which we do with a lookdown construction. Lookdown constructions were first introduced in Donnelly and Kurtz [1996] to provide a mechanism for retaining information about genealogical relationships between individuals sampled from a population evolving according to the Moran model when passing to the infinite population limit. Since then, they have been extended to a wide range of models. Of particular relevance to our work here are the papers of Kurtz and Rodrigues [2011] and Etheridge and Kurtz [2019], in which lookdown constructions are provided for a wide variety of population models, including spatially structured branching processes.

In general, even armed with a lookdown construction, calculation of relevant statistics of the genealogy remains a difficult question. However, in special circumstances, some progress can be made. As an illustration, we shall consider a scenario that has received a great deal of attention in recent years, in which a population is expanding into new territory as a travelling wave. In Section 3.2 we shall describe the motion of a single ancestral lineage relative to three different (deterministic) wavefronts across $R^{1}$.

Most work on the topic of “waves” of expanding populations has focused on models that caricature the classical Fisher-KPP equation with a stochastic term, i.e.

$$dw=(\Delta w+sw(1-w))dt+\sqrt{\frac{\alpha (w)}{N}}W(dt,dx),$$

where W is space-time white noise, and N is a measure of the local population density. The coefficient α(w) is generally taken to be either w, corresponding to a superprocess limit, or w(1-w) giving a spatial analogue of a Wright-Fisher diffusion. Starting with the pioneering work of Brunet et al. [2006], a considerable body of evidence has been amassed to underpin the conjecture that for this, and a wide class of related models, genealogies converge on suitable timescales in the infinite density limit to a Bolthausen-Sznitman coalescent. This reflects the fact that, for this equation, ancestral lineages become trapped in the wavefront, where the growth rate of the population is highest. Once there, they will experience rapid periods of coalescence corresponding to significant proportions of individuals in the front being descended from particularly reproductively successful ancestors.

If one replaces the logistic growth term of the classical Fisher-KPP equation with a nonlinearity that reflects cooperative behaviour in the population, such as

(1.4) $$wF\left(w\right)=w\left(1-w\right)\left(Cw-1\right),$$

then, for sufficiently large C (strong cooperation), the nature of the deterministic wave changes from “pulled” to “pushed”, [Birzu et al., 2018, 2021], and so the genealogies will be quite different from the Fisher-KPP case. For example, Etheridge and Penington [2022] show that for a discrete space model corresponding to this nonlinearity with C>2, after suitable scaling, the genealogy of a sample converges not to a Bolthausen-Sznitman coalescent, but to a Kingman coalescent. The reason, roughly, is that ancestral lineages settle to a stationary distribution relative to the position of the wavefront which puts very little weight close to the ‘tip’ of the wave, so that when ancestral lineages meet it is typically at a location in which population density is high, where no single ancestor produces a disproportionately large number of descendants in a short space of time.

The shape of the wave is not determined solely by the reaction term. For example, as a result of the nonlinear diffusion, for suitable initial conditions, the solution to the one-dimensional porous medium equation with logistic growth (1.2) converges to a travelling wave with a sharp cut-off; i.e., in contrast to the classical Fisher KPP equation, the solution at time t vanishes beyond $x=x_{0}+ct$ for some constant wavespeed c>0 [Kamin and Rosenau, 2004]. As a first step towards understanding what we should expect in models with nonlinear diffusion, one can ask about the position of an ancestral lineage relative to the wavefront in the deterministic models. In Section 3.2 we shall see that in our framework, even with logistic growth, the nonlinear diffusion corresponding to the porous medium equation results in a stationary distribution for the ancestral lineage that is concentrated behind the wavefront, leading us to conjecture that in the stochastic equation the cooperative behaviour captured by the nonlinear diffusion will also result in a qualitatively different pattern of coalescence to that seen under the stochastic Fisher-KPP equation. Indeed, we believe that it should be feasible to show that in an appropriate limit one recovers a Kingman coalescent.

### Structure of the paper

In this paper we study scaling limits of spatial population models, obtaining convergence of both the population process (i.e., the population density as a function of time, although strictly speaking it is a measure that may not have a density) and of lineages traced back through such a population. We retain information about lineages as we pass to the scaling limit by means of a lookdown construction.

In what follows we first study various scaling limits of the spatial population process, and then turn our attention to lineages traced back through these populations. First, in Section 2, we describe the model and the main results, Theorems 2.10, 2.20, and 2.23. Next, in Section 3, we discuss a few striking consequences of these results regarding the behavior of genealogies in traveling waves, the appearance of periodic “clumps” in seemingly homogeneous population models, and identifiability of the underlying dynamics from a stationary population profile. In Section 4, we provide heuristic explanations of why the theorems ought to be true, and some key ideas behind them, and in Section 5 we define and discuss the lookdown construction. Proofs of the results begin in Section 6, which proves results for population models with nonlocal interactions, while Section 7 gives the more difficult proof for the case when interaction distances also go to zero in the limit. Finally, Section 8 gives proofs for convergence of the lookdown process and the associated results for the motion of lineages. The Appendix contains a few more technical and less central lemmas. The results are illustrated in a few places with individual-based simulations, made using SLiM [Haller and Messer, 2019], but these are provided for visualization and we do not embark on numerical study.

## 2 Model and main results

Our model is one of individuals distributed across a continuous space which we shall take to be $R^{d}$. For applications, d=1 or d=2 (or even d=3 for cells within the body), but our main results apply more generally. At time zero, the population is distributed over a bounded region, with 𝒪(N) individuals per unit area in that region, so the total number of individuals will also be 𝒪(N). The population changes in continuous time, and we encode the state of the population at time t by a counting measure X(t), which assigns one unit of mass to the location of each individual.

Population dynamics are controlled by three quantities, birth (γ), establishment (r), and death (μ), each of which can depend on spatial location and local population density in a way specified below. Each individual gives birth at rate γ to a single (juvenile) offspring, which is dispersed according to a kernel q(x,⋅) away from the location x of the parent. We assume that q is the density of a multivariate Gaussian, allowing a nonzero mean and anisotropic variance. Both the mean and covariance of q can change across space, but do not depend on population density. The offspring does not necessarily survive to be counted in the population: it “establishes” with probability r, or else it dies immediately. Independently, each individual dies with rate μ.

We aim to capture universal behaviour by passing to a scaling limit. Specifically, we shall take the “density”, N, to infinity, and also scale time by a factor of θ=θ(N), in such a way that defining $\eta^{N}(t)=X(\theta t)/N$, the process ${\left\{\eta^{N}(t)\right\}}_{t\geq 0}$ will converge to a suitable measure-valued process as N and θ tend to infinity, with the nature of the limit depending on how they tend to infinity together. Evidently, we also need to scale the dispersal kernel if we are to obtain a nontrivial limit, for which we use $q_{\theta }(x,\cdot )$, the density of the multivariate Gaussian obtained by multiplying the mean and variance components of q(x,⋅) by 1/θ.

Birth, establishment, and death can depend on the location of the individual and the local population density. Since we would like the population density to scale with N, these are functions of X/N, i.e., the counting measure with mass 1/N placed at the location of each individual. First consider birth rates, defined by a nonnegative function $\gamma \left(x,m\right)\;:R^{d}\times R_{\geq 0}\to R_{\geq 0}$ of location x and local population density m. Local population density is defined as the convolution of X/N with a smooth (non-negative, integrable) kernel $\rho_{\gamma }(\cdot )$. We write this convolution as $\rho_{\gamma }*X/N$. Then, when the state of the population is X, an individual at location x gives birth to a single juvenile offspring at rate $\gamma \left(x,\rho_{\gamma }*X(x)/N\right)$. Similarly, the establishment probability of an offspring at location y is is $r\left(y,\rho_{r}*X(y)/N\right)$, where $r(y,m):R^{d}\times R_{\geq 0}\to [0,1]$ and again $\rho_{r}*X$ is the convolution of X/N with the smooth kernel $\rho_{r}$.

We shall write $\mu_{\theta }(x,X/N)$ for the per-capita death rate of mature individuals in the population. In order for the population density to change over timescales of order θ, we should like the net per capita reproductive rate to scale as 1/θ. In classical models, in which r,γ, and μ are constant, this quantity is simply rγ-μ. Here, because production of juveniles and their establishment are mediated by population density measured relative to different points, the net reproductive rate will take a more complicated form. In particular, the total rate of production of mature offspring by an individual at x will be

(2.1) $$\gamma \left(x,\rho_{\gamma }*X(x)/N\right)\int r\left(y,\rho_{r}*X(y)/N\right)q_{\theta }(x,dy).$$

Nonetheless, it will be convenient to define the death rate $\mu_{\theta }$ in terms of its deviation from rγ. To this end, we define the death rate of an individual at x to be

(2.2) $$\mu_{\theta }(x)=max\left\{0,r\left(x,\rho_{r}*X(x)/N\right)\gamma \left(x,\rho_{\gamma }*X(x)/N\right)-\frac{1}{\theta }F\left(x,\rho_{F}*X(x)/N\right)\right\},$$

where this equation defines $F(x,m):R^{d}\times R_{\geq 0}\to R$, and $\rho_{F}$ is again a smooth kernel. The function F is nearly the net per capita reproductive rate, scaled by θ, and would be equal to it in a nonspatial model; but, as can be seen from (2.1), differs because an offspring’s establishment probability is measured at their new location rather than that of their parent. For the most part, we work with F instead of $\mu_{\theta }$.

So, each of the three demographic parameters r,γ, and F, depends on local density, measured by convolution with a smooth kernel, each of which can be different. As a result, death rate depends (in principle) on population densities measured in three different ways, so that we could write $\mu_{\theta }(x)=\mu_{\theta }(x,\rho_{\gamma }*X(x)/N$, $\rho_{r}*X(x)/N,\;\rho_{F}*X(x)/N)$. This may seem unnecessarily complex. However, not only is it natural from a biological perspective, it also turns out to be convenient for capturing nontrivial examples in the scaling limit.

### Remark 2.1

Although this model allows fairly general birth and death mechanisms, there are a number of limitations. Perhaps most obviously, to simplify the notation individuals give birth to only one offspring at a time, although this restriction could be easily lifted [as in Section 3.4 of Etheridge and Kurtz, 2019]. Furthermore, individuals do not move during their lifetime, and the age of an individual does not affect its fecundity or death rate. Finally, there is no notion of mating (although limitations on reproduction due to availability of mates can be incorporated into the birth rate, γ), so the lineages we follow will be uniparental. For these reasons, the model is most obviously applicable to bacterial populations or selfing plants, although we do not anticipate that incorporation of these complications will change the general picture.

For each N and θ, we study primarily the process with mass scaled by N and time scaled by θ,

$${\left(\eta_{t}^{N}\right)}_{t\geq 0}:=(X(\theta t)/N{)}_{t\geq 0},$$

which takes values in the space of càdlàg paths in ${ℳ}_{F}\left(R^{d}\right)$ (the space of finite measures on $R^{d}$ endowed with the weak topology). In fact $\eta_{t}^{N}$ will be a purely atomic measure comprised of atoms of mass 1/N.

### Notation 2.2

*Expressions like* $\gamma \left(x,\rho_{\gamma }*\eta (x)\right)$ *will appear repeatedly in what follows. To make formulae more readable, we overload notation to define*

$$\gamma \left(x,\eta \right):=\gamma \left(x,\rho_{\gamma }*\eta \left(x\right)\right),$$

*and similarly write* r(x,η) for $r\left(x,\rho_{r}*\eta (x)\right),\;F(x,\eta )$ for $F\left(x,\rho_{F}*\eta (x)\right)$, *and* $\mu_{\theta }(x,\eta )$ *for the expression of* equation (2.2). When convenient, we may also suppress the arguments completely, writing simply γ,r,F, *and* $\mu_{\theta }$ *for these quantities*.

### Terminology 2.3

*In our prelimiting model, the population is represented by a point measure in which each individual is assigned a mass* 1/N. *We use the term “population density” for this process, as it is supposed to measure population size relative to a nominal occupancy of* N *individuals per unit area. There is no implication that the measure representing the population is absolutely continuous with respect to Lebesgue measure; indeed in the prelimit it is certainly not*.

In summary, at each time $t,\;\eta_{t}^{N}$ is purely atomic, consisting of atoms of mass 1/N (which are the individuals). At instantaneous rate $\theta \gamma \left(x,\eta_{t}^{N}\right)N\eta_{t}^{N}(dx)$ an offspring of mass 1/N is produced at location x; it disperses to a location y offset from x by an independent Gaussian random variable with mean $\vec{b}(x)/\theta$ and covariance matrix C(x)/θ, and once there establishes instantaneously with probability $r\left(y,\eta_{t}^{N}\right)$, or else dies. At instantaneous rate $\theta \mu_{\theta }\left(x,\eta_{t}^{N}\right)N\eta_{t}^{N}(dx)$ an individual at location x dies. Note that the process ${\left(\eta_{t}^{N}\right)}_{t\geq 0}$, which records numbers and locations of adult individuals, is just a scaled spatial birth and death process. If, for example, we insist that γ(x,m) is bounded, then existence (and in particular non-explosion) is guaranteed by comparison with a pure birth process. We do not dwell on this, as we shall require more stringent conditions if we are to pass to the limit as θ and N tend to infinity.

It is convenient to characterise the process as a solution to a martingale problem. We write $C_{b}^{\infty }\left(R^{d}\right)$ for the space of bounded smooth functions on $R^{d}$, and, where convenient, we write $\left\langle f,\eta \rangle =\int_{R^{d}}\;f(x)\eta (dx\right)$.

### Definition 2.4 (Martingale Problem Characterisation)

For each value of N and θ, and each purely atomic $\eta_{0}^{N}\in {ℳ}_{F}\left(R^{d}\right)$ *with atoms of mass* $1/N,\;{\left(\eta_{t}^{N}\right)}_{t\geq 0}$ *is the (scaled) empirical measure of a birth-death process with càdlàg paths in* ${ℳ}_{F}\left(R^{d}\right)$ *for which, for all* $f\in C_{b}^{\infty }\left(R^{d}\right)$, *writing* $q_{\theta }(x,dy)$ *for the Gaussian kernel with mean* $x+\vec{b}(x)/\theta$ *and covariance* C(x)/θ,

(2.3) $$M_{t}^{N}(f):=\left\langle f,\eta_{t}^{N}\right\rangle -\left\langle f,\eta_{0}^{N}\right\rangle \;\;-\int_{0}^{t}\;\;\{\langle (\int \theta \left(f(z)r\left(z,\eta_{s}^{N}\right)-f(x)r\left(x,\eta_{s}^{N}\right)\right)q_{\theta }(x,dz))\gamma \left(x,\eta_{s}^{N}\right),\eta_{s}^{N}(dx)\rangle \;\;+\langle f(x)F\left(x,\eta_{s}^{N}\right),\eta_{s}^{N}(dx)\rangle \}ds$$

is a martingale (with respect to the natural filtration), with angle bracket process

(2.4) $${\left\langle M^{N}(f)\right\rangle }_{t}=\frac{\theta }{N}\int_{0}^{t}\;\{\langle \gamma \left(x,\eta_{s}^{N}\right)\int f^{2}(z)r\left(z,\eta_{s}^{N}\right)q_{\theta }(x,dz),\eta_{s}^{N}(dx)\rangle \;\;+\langle \mu_{\theta }\left(x,\eta_{s}^{N}\right)f^{2}(x),\eta_{s}^{N}(dx)\rangle \}ds.$$

The angle bracket process (or, “conditional quadratic variation”) is the unique previsible process making ${\left(M^{N}(f{)}_{t}\right)}^{2}-{\left\langle M^{N}(f)\right\rangle }_{t}$ a martingale with respect to the natural filtration. It differs from the usual quadratic variation (usually denoted ${\left[M^{N}(f)\right]}_{t}$) because the process has jumps; for the (continuous) limit the two notions will coincide. The use of angle brackets for both integrals and this process is unfortunately standard but should not cause confusion, since the angle bracket process always carries a subscript for time.

The form of (2.3) and (2.4) is explained in Section 4. Note that since (juvenile) individuals are produced at rate Nγη, but each has mass 1/N, these factors of N cancel in (2.3). Under our scaling, N and θ=θ(N) will tend to infinity in such a way that $\alpha :={lim}_{N\to \infty }\;\theta (N)/N$ exists and is finite. From the expression (2.4) it is easy to guess that whether the limiting processes will be deterministic or stochastic is determined by whether α is zero or nonzero.

It is convenient to record some notation for the generator of the diffusion limit of a random walk with jump distribution determined by $q_{\theta }(x,dy)$.

### Definition 2.5 (Dispersal generator)

*As above, we define the dispersal kernel*, $q_{\theta }(x,dy)$, *to be the density of a multivariate Gaussian with mean* $\vec{b}(x)/\theta$ *and covariance matrix* C(x)/θ *(although often we omit the dependence of* $\vec{b}$ *and* C *on* x). *Furthermore, we define for* $f\in C_{b}^{\infty }\left(R^{d}\right)$,

(2.5) $$ℬf\left(x\right)=\sum_{ij}C{\left(x\right)}_{ij}\partial_{x_{i}}\partial_{x_{j}}f\left(x\right)+\sum_{i}\vec{b}{\left(x\right)}_{i}\partial_{x_{i}}f\left(x\right)$$

*and denote the adjoint of* ℬ *by*

$${ℬ}^{*}f(x)=\;\sum_{ij}\;\partial_{x_{i}}\partial_{x_{j}}(C(x{)}_{ij}f(x))-\sum_{i}\;\partial_{x_{i}}(f(x)\vec{b}(x{)}_{i})\;\;=\;\sum_{ij}\;C_{ij}(x)\partial_{x_{i}}\partial_{x_{j}}f(x)+\sum_{i}\;\left(\sum_{j}\;\partial_{x_{j}}C_{ij}(x)-{\vec{b}}_{i}(x)\right)\partial_{x_{i}}f(x)\;\;+\left(\sum_{ij}\;\partial_{x_{i}}\partial_{x_{j}}C_{ij}(x)-\sum_{i}\;\partial_{x_{i}}{\vec{b}}_{i}(x)\right)f(x).$$

### Remark 2.6

ℬ *is defined so that*

$$\theta \int \left(f\left(y\right)-f\left(x\right)\right)q_{\theta }\left(x,dy\right)\to ℬf\left(x\right)\;as\;\theta \to \infty .$$

### Remark 2.7

*An equivalent way to describe the model would be to say that when the state of the population is* η, *an individual at* x *gives birth at rate*

$$\theta \gamma \left(x,\eta \right)\int r\left(y,\eta \right)q\left(x,dy\right),$$

and that offspring disperse according to the kernel

$$q_{\theta }^{m}(x,\eta ,dy):=\frac{r(y,\;\eta )q_{\theta }(x,\;dy)}{\int r(z,\;\eta )q_{\theta }(x,\;dz)}.$$

Clearly, the random walk driven by this dispersal kernel is biased towards regions of higher establishment probability. For comparison with future results, it is interesting to write down the limiting generator:

(2.6) $$\underset{\theta \to \infty }{lim}\;\theta \int (f(y)-f(x))q_{\theta }^{m}(x,\eta ,dy)=\frac{ℬ[f(\cdot )r(\cdot ,\eta )](x)-f(x)ℬ[r(\cdot ,\eta )](x)}{r\left(x,\eta^{N}\right)}.$$

*In the simplest case of unbiased isotropic dispersal* (*i.e*., $\vec{b}=0$ and C=I),ℬ=Δ, *and so* (2.6) *is equal to*

$$\Delta f\left(x\right)+2\nabla f\left(x\right)\cdot \nabla \log r\left(\cdot ,\rho_{r}*\eta \left(\cdot \right)\right)\left(x\right).$$

*One might guess that the spatial motion described by following the ancestral lineage of an individual back through time would be described (in the limit) by the adjoint of this generator. However, we will see in* Section 2.2 *that this is not in fact the case.*

In order to pass to a scaling limit, we will need to impose some conditions on the parameters of our model.

### Assumptions 2.8

*We shall make the following assumptions on the parameters of our model*.

#### Dispersal generator:

We assume that

1. $\vec{b}(x)$ *and* C(x) are α-Hölder continuous for some α∈(0,1] and uniformly bounded in each component, and
2. the operator ℬ is uniformly strictly elliptic, i.e., ${inf}_{x}\;{\;inf}_{y:∥y∥=1}\;\sum_{ij}\;y_{i}C(x{)}_{ij}y_{j}>0$.

#### Reproduction parameters:

We assume that

- The function F(x,m) satisfies
  - F(x,m) is locally Lipschitz in m;
  - F(x,m) is uniformly bounded above (but not necessarily below);
  - for each fixed $m,\;{sup}_{x\in R^{d}}\;{sup}_{k\leq m}\;|F(x,k)|<\infty$;
- The functions r(x,m), γ(x,m) have bounded first and second derivatives in both arguments;
- γ(x,m) is uniformly bounded;
- *For each* $f\in C_{b}^{2}\left(R^{d}\right)$, there is a $C_{f}$ such that
$$\left|\gamma (x,\eta )\theta \int (r(y,\eta )f(y)-r(x,\eta )f(x))q_{\theta }(x,dy)\right|\leq C_{f}(1+|f(x)|)$$
  *for all* $x\in R^{d}$ *and* $\eta \in {ℳ}_{F}\left(R^{d}\right)$. *Furthermore*, $C_{f}$ only depends on the norm of the first two derivatives of f, i.e.,
$$C_{f}=C\left(\underset{x}{\sup}\underset{\|z\|=1}{\sup}\max\left(\sum_{i}z_{i}\partial_{x_{i}}f(x),\sum_{ij}z_{i}z_{j}\partial_{x_{i}x_{j}}f(x)\right)\right).$$
  *To keep expressions manageable, we shall also assume that*
$$\mu_{\theta }(x)=r(x,\eta )\gamma (x,\eta )-\frac{1}{\theta }F(x,\eta ),$$
  that is, this expression is non-negative so that there is no need to take the maximum with zero in (2.2). (This is anyway true for sufficiently large θ.)

Since we take bounded f, for most situations the bound $C_{f}(1+|f(x)|)$ in Condition 6 above can be safely replaced simply by $C_{f}$; however, this will be useful in certain situations where we consider a sequence of f with increasing upper bounds. We now give two concrete situations in which Condition 6 is satisfied. The proof is in Section 6.1.

### Lemma 2.9

Assume that Conditions 2.8 are satisfied, except for Condition 6. If either

1. $\left|\partial_{x_{i}}r(x,\eta )\right|$ *and* $\left|\partial_{x_{i}x_{j}}r(x,\eta )\right|$ are uniformly bounded for $x\in R^{d},\;\eta \in {ℳ}_{F}\left(R^{d}\right)$;
2. *or*, $m^{2}\gamma (x,m)$ is uniformly bounded and there exists C<∞ such that for θ sufficiently large, and all $x\in R^{d},\;\eta \in {ℳ}_{F}\left(R^{d}\right)$,
$$\theta \int \left(\rho_{r}*\eta \left(y\right)-\rho_{r}*\eta \left(x\right)\right)q_{\theta }\left(x,dy\right)\leq C\rho_{\gamma }*\eta \left(x\right),$$
  *and*
$$\theta \int {\left(\rho_{r}*\eta \left(y\right)-\rho_{r}*\eta \left(x\right)\right)}^{2}q_{\theta }\left(x,dy\right)\leq C{\left(\rho_{\gamma }*\eta \left(x\right)\right)}^{2},$$
  then Condition 6 is also satisfied.

The purpose of the conditions that we have placed on the reproduction parameters is to ensure that the net per capita reproduction rate (before time scaling) is order 1/θ. As remarked above, because of the non-local reproduction mechanism, it no longer suffices to assume that $r(x,\eta )\gamma (x,\eta )-\mu_{\theta }(x)$ is of order 1/θ. Perhaps the simplest example in which we can see this is the case where γ≡1 and F≡0, so that $\mu_{\theta }=r$, and $\eta =\delta_{x}$ (i.e., the population has all individuals at a single location), so that $\rho_{r}*\eta (y)=\rho_{r}(y)$. In this case, the mean rate of change of the total population size is $\int \left(r\left(y,\rho_{r}(y)\right)-r\left(x,\rho_{r}(x)\right)\right)q_{\theta }(x,dy)$; the first condition of Lemma 2.9 would ensure this is of order 1/θ.

If r(x,m) is independent of m, then the conditions are easy to satisfy; they just require some regularity of r as a function of x. Condition 1 of Lemma 2.9 is also satisfied if for example $∥\nabla \rho_{r}∥\leq C\rho_{r}$ and $m\partial_{m}r(x,m)$, $m^{2}\partial_{mm}r(x,m)$ are bounded. This is the case, for instance, if $\rho_{r}$ decays exponentially. On the other hand, it might seem more natural to take $\rho_{r}$ to be a Gaussian density with parameter $\sigma_{r}$, say. Then, as we check in Lemma B.1, Condition 2 of Lemma 2.9 is satisfied if $\rho_{\gamma }$ is also Gaussian with parameter $\sigma_{\gamma }$ and $\sigma_{\gamma }>\sigma_{r}$. For large enough θ, this condition guarantees that $\sigma_{r}+1/\theta <\sigma_{\gamma }$, so that the establishment probability of a juvenile is controlled by individuals that are already ‘felt’ by the fecundity-regulating kernel $\rho_{\gamma }$ at the location of their parent.

### 2.1 Scaling limits of the population process

**Figure 1:**
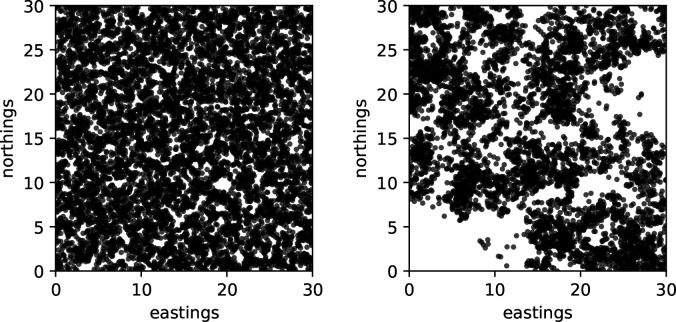
Snapshots of two simulations, with small α=θ/N (left) and large α=θ/N (right). Simulations are run with a Fisher-KPP-like parameterization: birth and establishment are constant, while death increases linearly with density, at slope 1/θ. Left: α=0.1. Right: α=10. Other parameters were the same: dispersal and interactions distance were set to 1, and the equilibrium density is 10 individuals per unit area.

Our main results depend on two dichotomies: Is the limiting process deterministic or a (generalized) superprocess? And, are interactions pointwise in the limit or nonlocal? See Figure 1 for snapshots of the population from direct simulation of the process using SLiM [Haller and Messer, 2019] illustrating this first dichotomy. Below we have results for deterministic limits with pointwise and nonlocal interactions, and for superprocess limits with nonlocal interactions.

#### Scaling limits with nonlocal interactions:

Recall that the process ${\left(\eta_{t}^{N}\right)}_{t\geq 0}$ takes its values in the space ${𝒟}_{\left[0,\infty \right)}\left({ℳ}_{F}\left(R^{d}\right)\right)$ of càdlàg paths on ${ℳ}_{F}\left(R^{d}\right)$. We endow ${ℳ}_{F}\left(R^{d}\right)$ with the topology of weak convergence and ${𝒟}_{\left[0,\infty \right)}\left({ℳ}_{F}\left(R^{d}\right)\right)$ with the Skorohod topology. A sequence of processes taking values in ${𝒟}_{\left[0,\infty \right)}\left({ℳ}_{F}\left(R^{d}\right)\right)$ is said to be tight if the corresponding sequence of distributions is tight, i.e., that any infinite subsequence has a weakly convergent subsubsequence. Our first main result establishes tightness of our rescaled population processes in the case in which interactions remain nonlocal under the scaling, and characterises limit points as solutions to a martingale problem.

#### Theorem 2.10

*Let* ${\left(\eta_{t}^{N}\right)}_{t\geq 0}$ *be as defined in* Definition 2.4 *and assume that as* N→∞,θ(N)→∞ *in such a way that* θ(N)/N→α. *(However, the kernels* $\rho_{r},\;\rho_{\gamma }$, *and* $\rho_{F}$ *remain fixed.) Suppose that* Assumptions 2.8 *hold and, further, that* ${\left\{\eta_{0}^{N}\right\}}_{N\geq 1}$ *is a sequence of purely atomic measures, with* $\eta_{0}^{N}$ *comprised of atoms of mass* 1/N, *which is tight in* ${ℳ}_{F}\left(R^{d}\right)$. *Also assume there exists a nonnegative* $f_{0}\in C\left(R^{d}\right)$ *with uniformly bounded first and second derivatives (i.e., with* ${sup}_{x}\;{sup}_{∥z∥=1}\;\sum_{i}\;\partial_{x_{i}}f_{0}(x)z_{i}$ *and* ${sup}_{x}\;{sup}_{∥z∥=1}\;\sum_{ij}\;\partial_{x_{i}x_{j}}f_{0}(x)z_{i}z_{j}$ *both finite) and* $f_{0}(x)\to \infty$ *as* |x|→∞ *for which* $\left\langle f_{0}(x),\eta_{0}^{N}(dx)\right\rangle <C<\infty$ *for some* C *independent of* N. *Then the sequence of processes* ${\left(\eta_{t}^{N}\right)}_{t\geq 0}$ *is tight, and for any limit point* ${\left(\eta_{t}\right)}_{t\geq 0}$, *for every* $f\in C_{b}^{\infty }\left(R^{d}\right),$

(2.7) $$M_{t}(f):=\langle f(x),\eta_{t}(dx)\rangle -\langle f(x),\eta_{0}(dx)\rangle \;\;-\int_{0}^{t}\left\langle \gamma \left(x,\eta_{s}\right)ℬ\left(f(\cdot )r\left(\cdot ,\eta_{s}\right)\right)(x)+f(x)F\left(x,\eta_{s}\right),\eta_{s}(dx)\right\rangle ds$$

is a martingale (with respect to the natural filtration), with angle bracket process

(2.8) $$\langle M(f)\rangle_{t}=\alpha \int_{0}^{t}\;\left\langle 2\gamma \left(x,\eta_{s}\right)r\left(x,\eta_{s}\right)f^{2}\left(x\right),\eta_{s}\left(dx\right)\right\rangle ds.$$

*If* α=0 *the limit is deterministic*.

Recall when interpreting (2.7) that, for instance, $r\left(x,\eta_{s}\right)=r\left(x,\rho_{r}*\eta_{s}(x)\right)$, and so $ℬ(fr)(x)=ℬ\left(f(\cdot )r\left(\cdot ,\rho_{r}*\eta_{s}(\cdot )\right)\right)(x)$. The proof of this theorem appears in Section 6.2.

Theorem 2.10 provides tightness of the rescaled processes. If the limit points are unique, then this is enough to guarantee convergence.

#### Corollary 2.11

*Under the assumptions of Theorem 2.10, if the martingale problem defined by* equations (2.7) *and* (2.8) *has a unique solution, then* ${\left(\eta_{t}^{N}\right)}_{t\geq 0}$ *converges weakly to that solution as* N→∞.

When α>0, the limit points can be thought of as interacting superprocesses. For example, when r and γ are constant, and $F\left(x,\eta_{s}\right)=1-\rho_{F}*\eta_{s}(x)$, we recover a superprocess with nonlinear death rates corresponding to logistic growth [Etheridge, 2004] that is a continuous limit of the Bolker-Pacala model [Bolker and Pacala, 1997, 1999]. We are not aware of a general result to determine when we will have uniqueness of solutions to the martingale problem of Theorem 2.10 when α>0. However, the Dawson–Girsanov transform tells us that we have uniqueness in this special case of the superprocess with nonlinear death rates, and Perkins stochastic calculus (and its adaptation to a lookdown setting) provides uniqueness for cases with interactions in the dispersal mechanism of the superprocess. We refer to Dawson [1993], Perkins [1992], and Donnelly and Kurtz [1999] for approaches to showing that these sorts of martingale problems are well-posed.

For the deterministic case of α=0, the limiting process is a weak solution to a nonlocal PDE. We next describe some situations in which more is known about uniqueness and whether the solution is close to the corresponding local PDE. First, recall the following notion of solution to a PDE.

#### Definition 2.12 (Weak solutions)

*We say that* ${\left(\eta_{t}\right)}_{t\geq 0}$, *with* $\eta_{t}\in {ℳ}_{F}\left(R^{d}\right)$, *is a weak solution to the PDE*

(2.9) $$\partial_{t}\varphi =r{ℬ}^{*}(\gamma \varphi )+\varphi F$$

(where r,γ and F can all be functions of φ) if, for all $f\in C_{b}^{\infty }\left(R^{d}\right)$,

$$\frac{d}{dt}\left\langle f,\eta_{t}\right\rangle =\left\langle \gamma ℬ(rf)+fF,\eta_{t}\right\rangle .$$

The notation φ is meant to be suggestive of a density, and recall that equation (2.9) has made dependencies on x and φ implicit; written out more explicitly, (2.9) is

$$\partial_{t}\varphi_{t}(x)=r\left(x,\rho_{r}*\varphi_{t}(x)\right){ℬ}^{*}\left[\varphi_{t}(\cdot )\gamma \left(x,\rho_{\gamma }*\varphi_{t}(\cdot )\right)\right](x)+\varphi_{t}(x)F\left(x,\rho_{F}*\varphi_{t}(x)\right).$$

Because Theorem 2.10 only tells us about weak convergence, in the case α=0 we can only deduce that any limit point $\eta_{t}$ is a weak solution to this nonlocal PDE.

Specialising the results of Kurtz and Xiong [1999] to the deterministic setting provides general conditions under which we have existence and uniqueness of solutions to (2.9) which have an $L^{2}$-density with respect to Lebesgue measure. Recall that the Wasserstein metric, defined by

$$\rho \left(\nu_{1},\nu_{2}\right)=sup\left\{|\int fd\nu_{1}-\int fd\nu_{2}|:\underset{x}{sup}\;|f(x)|\leq 1,|f(x)-f(y)|\leq ∥x-y∥\right\},$$

determines the topology of weak convergence on ${ℳ}_{F}\left(R^{d}\right)$. We write $r(x,\eta )\gamma (x,\eta )C(x)=J(x,\eta )J(x,\eta {)}^{T}$, and $\beta (x,\eta )=r(x,\eta )\gamma (x,\eta )(\vec{b}(x)+2C(x)\nabla \;logr(x,\eta ))$ (quantities that will appear in Proposition 5.6). If J,β, and F are bounded and Lipschitz in the sense that

(2.10) $$\left|J\left(x_{1},\nu_{1}\right)-J\left(x_{2},\nu_{2}\right)\right|,\left|\beta \left(x_{1},\nu_{1}\right)-\beta \left(x_{2},\nu_{2}\right)\right|,\left|F\left(x_{1},\nu_{1}\right)-F\left(x_{2},\nu_{2}\right)\right|\leq C\left(∥x_{1}-x_{2}∥+\rho \left(\nu_{1},\nu_{2}\right)\right)$$

for some C>0, the methods of Kurtz and Xiong [1999] show that if the initial condition $\eta_{0}$ for our population process has an $L^{2}$ density, then so does $\eta_{t}$ for t>0. Although the necessary estimates (for which we refer to the original paper) are highly nontrivial, the idea of the proof is simple. Take a solution to the equation and use it to calculate the coefficients r,γ and F that depend on local population density. Then η solves the *linear* equation obtained by regarding those values of r,γ and F as given. It remains to prove that the solution to the linear equation has a density which is achieved by obtaining $L^{2}$ bounds on its convolution with the heat semigroup at time δ and letting δ→0. We also have the following uniqueness result.

#### Theorem 2.13 (Special case of Kurtz and Xiong [1999], Theorem 3.5)

*Suppose* J,β, *and* F *are bounded and Lipschitz in the sense of* (2.10). *If* $\eta_{0}$ *has an* $L^{2}\left(R^{d}\right)$-*density, then there exists a unique* $L^{2}\left(R^{d}\right)$-*valued solution of* (2.9) *in the sense of* Definition 2.12.

#### Remark 2.14

*Kurtz and Xiong [1999] considers an infinite system of stochastic differential equations for the locations and weights of a collection of particles that interact through their weighted empirical measure, which is shown to be the unique solution to a stochastic PDE. As we shall see through our lookdown representation in* Section 5, *the solution to our deterministic equation can be seen as the empirical measure of a countable number of particles (all with the same weight) which, in the notation above, evolve according to*

$$X(t)=X(0)+\int_{0}^{t}\;\beta \left(X(s),\eta_{s}\right)ds+\int_{0}^{t}\;J\left(X(s),\eta_{s}\right)dW(s)$$

(with an independent Brownian motion W for each particle).

#### Two-step convergence to PDE:

Although the coefficients at x in (2.9) are nonlocal, we can choose our kernels $\rho_{\gamma },\;\rho_{r}$, and $\rho_{F}$ in such a way that they depend only on the population in a region close to x, and so we expect that under rather general conditions solutions of the nonlocal PDE will be close to the corresponding classical PDE. The following propositions provide two concrete situations in which this is true. In the first, the PDE is a reaction-diffusion equation, and in the proof in Section 6.3.1 we borrow an idea from Penington [2017] to express the solutions to both the nonlocal equation and the classical PDE through a Feynman-Kac formula.

#### Proposition 2.15

*Let* $\rho_{F}^{\epsilon }(x)=\rho_{F}(x/\epsilon )/\epsilon^{d}$. *Assume* $\varphi_{0}\in L^{2}(R)$ is a positive, uniformly Lipschitz, and uniformly bounded function. Suppose that $\varphi^{\epsilon }\in L^{2}\left(R^{d}\right)$ is a weak solution to the equation

(2.11) $$\partial_{t}\varphi^{\epsilon }={ℬ}^{*}\varphi^{\epsilon }+\varphi^{\epsilon }F\left(\rho_{F}^{\epsilon }*\varphi^{\epsilon }\right),\;x\in R^{d},t>0,$$

with initial condition $\varphi_{0}(\cdot )$, and that φ is a weak solution to the equation

(2.12) $$\partial_{t}\varphi ={ℬ}^{*}\varphi +\varphi F(\varphi ),\;x\in R^{d},t>0,$$

*also with initial condition* $\varphi_{0}(\cdot )$. *Suppose further that* F *is a Lipschitz function which is bounded above, and that* $\vec{b}(x)$ *and* C(x), *the drift and covariance matrix of* ℬ, *satisfy the conditions of* Assumptions 2.8. *Then, for all* T>0 *there exists a constant* $K=K\left(T,{∥\varphi_{0}∥}_{\infty }\right)<\infty$ *and a function* δ(ϵ) *(dependent on* $\rho_{F}$*) with* δ(ϵ)→0 *as* ϵ→0, *such that, for all* 0≤t≤T, *and* ϵ *small enough*,

$${∥\varphi_{t}(\cdot )-\varphi_{t}^{\epsilon }(\cdot )∥}_{\infty }\leq K\delta (\epsilon ).$$

*In particular, as* ϵ→0, *we have that* $\varphi^{\epsilon }$ *converges uniformly in compact intervals of time to* φ.

#### Remark 2.16

Note that Theorem 2.13 guarantees uniqueness of solutions to equation (2.11).

Our second example in which we know solutions to the nonlocal PDE converge to solutions of the local PDE as interaction distances go to zero is a nonlocal version of a porous medium equation with logistic growth. That is, we consider non-negative solutions to the equation

(2.13) $$\partial_{t}\psi^{\epsilon }=\Delta \left(\psi^{\epsilon }\rho_{\gamma }^{\epsilon }*\psi^{\epsilon }\right)+\psi^{\epsilon }\left(1-\rho_{\gamma }^{\epsilon }*\psi^{\epsilon }\right).$$

The case without the reaction term (and with $R^{d}$ replaced by a torus) is considered by Lions and Mas-Gallic [2001] who use it as a basis for a particle method for numerical solution of the porous medium equation. Of course this does not quite fit into our framework, since in the notation of our population models this would necessitate $\gamma (x,m)=\rho_{\epsilon }*m$ which is not bounded. However, this can be overcome by an additional layer of approximation (c.f. our numerical experiments of Section 3.1) and we do not allow this to detain us here. Existence and uniqueness of solutions to (2.13) can be obtained using the approach of Lions and Mas-Gallic [2001], so we should like to prove that as ϵ→0 we have convergence to the solution to the porous medium equation with logistic growth:

(2.14) $$\partial_{t}\psi =\Delta \left(\psi^{2}\right)+\psi (1-\psi ).$$

#### Notation 2.17

*We use* ⇀ *to denote weak convergence in the sense of analysts; that is,* $\psi^{\epsilon }⇀\psi$ *in* $L^{1}$ *means* $\int \psi^{\epsilon }vdx\to \int \psi vdx$ *for all* $v\in L^{\infty }$.

*We write* $L_{t}^{2}\left(H^{1}\right)$ *for functions for which the $H^{1}$ norm in space is in* $L^{2}$ *with respect to time, i.e.*

$$\int_{0}^{T}\;\int \{\psi_{t}(x{)}^{2}+{∥\nabla \psi_{t}(x)∥}^{2}\}dxdt<\infty ,$$

and $C_{t}\left(L^{1}\right)$ will denote functions for which the $L^{1}$ norm in space is continuous in time.

#### Proposition 2.18

*Suppose that we can write* $\rho_{\gamma }=\zeta *\overset{ˇ}{\zeta }$, *where* $\overset{ˇ}{\zeta }(x)=\zeta (-x)$ *and* $\zeta \in 𝒮\left(R^{d}\right)$ *(the Schwartz space of rapidly decreasing functions). Furthermore, suppose that* $\psi_{0}^{\epsilon }\geq 0$ *is such that there exist* λ∈(0,1) *and* C∈(0,∞) *(independent of* ϵ) such that

$$\int exp(\lambda ∥x∥)\psi_{0}^{\epsilon }(x)dx<C,\;and\;\underset{\epsilon }{sup}\;\int \psi_{0}^{\epsilon }\left|log\psi_{0}^{\epsilon }\right|dx<\infty ,$$

*with* $\psi_{0}^{\epsilon }⇀\psi_{0}$ *as* ϵ→0. *Then writing* $\psi^{\epsilon }$ *for the solution to* (2.13) *on* $[0,T]\times R^{d}$ *with initial condition* $\psi_{0}^{\epsilon }$, $\psi^{\epsilon }⇀\psi_{0}$ *as* ϵ→0 where $\psi \in L_{t}^{2}\left(H^{1}\right)\cap C_{t}\left(L^{1}\right),\;\int \psi |log\psi |dx<\infty$, *and* ψ *solves* (2.14) *on* $[0,T]\times R^{d}$.

The example that we have in mind for the kernel $\rho_{\gamma }$ is a Gaussian kernel. For the proof, see Section 6.3.2.

#### Remark 2.19

*Although it seems hard to formulate an all-encompassing result, Propositions 2.15 and 2.18 are by no means exhaustive. When the scaling limit is deterministic, one can expect analogous results under rather general conditions. However, when the limit points are stochastic, they resemble “nonlinear superprocesses” and so one cannot expect a density with respect to Lebesgue measure in* d≥2. *It is then not reasonable to expect to be able to make sense of the limit if we scale the kernels in this way. Moreover, in one dimension, where the classical superprocess does have a density with respect to Lebesgue measure, the form of (2.7) suggests that even if one can remove the local averaging from* γ, *it will be necessary to retain averaging of* r *in order to obtain a well-defined limit*.

#### One-step convergence to PDE:

Theorem 2.10, combined with Proposition 2.15 or 2.18 implies that we can take the limit N→∞ followed by the limit ϵ→0 to obtain solutions to the PDE (2.12). However, it is of substantial interest to know whether we can take those two limits simultaneously. The general case seems difficult, but we prove such “diagonal” convergence in the following situation. The proof is provided in Section 7.

#### Theorem 2.20 (Convergence to a PDE)

*Let* ${\left(\eta_{t}^{N}\right)}_{t\geq 0}$ *be as defined in* Definition 2.4 *with* r(x,m)≡1≡γ(r,m), F(x,m)≡F(m), $\rho_{F}^{\epsilon }$ *a symmetric Gaussian density with variance parameter* $\epsilon^{2}$, *and* ℬ=Δ/2. *Further suppose that* F(m) *is a polynomial with* $F(m)1_{m\geq 0}$ *bounded above. Assume that* $\left\langle 1,\eta_{0}^{N}\right\rangle$ *is uniformly bounded, and that for all* $x\in R^{d}$ *and* k∈N,

$$\underset{\epsilon \to 0}{lim\;sup}\;E[\rho_{F}^{\epsilon }*\eta_{0}(x{)}^{k}]<\infty ,$$

*and*

$$\underset{\epsilon \to 0}{lim\;sup}\;\int E[\rho_{F}^{\epsilon }*\eta_{0}(x{)}^{k}]dx<\infty .$$

Finally assume that N→∞,θ→∞ *and* ϵ→0 *in such a way that*

(2.15) $$\frac{1}{\theta \epsilon^{2}}+\frac{\theta }{N\epsilon^{d}}\to 0.$$

*Then the sequence of* ${𝒟}_{\left[0,\infty \right)}\left({ℳ}_{F}\left(R^{d}\right)\right)$-*valued stochastic processes* ${\left(\rho_{F}^{\epsilon }*\eta_{t}^{N}(x)dx\right)}_{t\geq 0}$ *converges weakly to a measure-valued process with a density* φ(t,x) *that solves*

(2.16) $$\partial_{t}\varphi \left(t,x\right)=\frac{1}{2}\Delta \varphi \left(t,x\right)+\varphi \left(t,x\right)F\left(\varphi \left(t,x\right)\right).$$

#### Remark 2.21

In fact, our proof goes through without significant change under the conditions that $F(m)1_{m\geq 0}$ is bounded above (but not necessarily below), and that for all m,n∈[0,∞)

$$|F(m)|\leq \sum_{j=1}^{k}a_{j}m^{j},\;\;\;\;\;and\;|F(n)-F(m)|\leq |n-m|\sum_{j=1}^{k^{\prime }}b_{j}\left(n^{j}+m^{j}\right),$$

*for some non-negative constants* ${\left\{a_{j}\right\}}_{j=0}^{k},\;{\left\{b_{j}\right\}}_{j=0}^{k^{\prime }}$. *We take* F *to be polynomial to somewhat simplify notation in the proof*.

### 2.2 Ancestral lineages in the scaling limit

Now that we have established what we can say about how population density changes with time, we turn to results on ancestral lineages, i.e., how genealogical ancestry can be traced back across the landscape. Informally, a *lineage* ${\left(L_{t}^{N}\right)}_{t\geq 0}$, begun at a spatial location $L_{0}^{N}=x$ where there is a focal individual in the present day can be obtained by, for each time t, setting $L_{t}^{N}$ to be the spatial location of the individual alive at time t before the present from whom the focal individual is descended. Since in our model individuals have only one parent, this is unambiguous. Although we did not explicitly retain such information, it is clear that for finite N, since individuals are born one at a time, one could construct the lineage ${\left(L_{t}^{N}\right)}_{t=0}^{T}$ given the history of the population ${\left(\eta_{t}^{N}\right)}_{t=0}^{T}$, for each starting location to which $\eta_{T}^{N}$ assigns positive mass. It is less clear, however, how to formally retain such information when we pass to the scaling limit.

The *lookdown construction* in Section 5 will enable us to recover information about ancestry in the infinite population limit. Roughly speaking, each particle is assigned a unique “level” from [0,∞) that functions as a label and thus allows reconstruction of lineages. The key to the approach is that levels are assigned in such a way as to be exchangeable, so that sampling a finite number, k say, of individuals from a given region is equivalent to looking at the individuals in that region with the k lowest levels. Moreover, as we pass to the infinite population limit, the collection of (individual, level) pairs converges, as we show in Theorem 5.4. See Etheridge and Kurtz [2019] for an introduction to these ideas. In particular, even in the infinite population limit, we can sample an individual from a region (it will be the individual in that region with the lowest level) and trace its line of descent. This will allow us to calculate, for each x and $y\in R^{d}$, the proportion of the population at location x in the present day population that are descended from a parent who was at location y at time t in the past. To make sense of this in our framework, in Section 8.2, we justify a weak reformulation of this idea.

We are interested in two questions. First, when is the motion of an ancestral lineage, given complete knowledge of the population process, a well-defined process? In other words, is knowledge of the process ${\left(\eta_{t}\right)}_{t=0}^{T}$ that records numbers of individuals but not their ancestry sufficient to define the distribution of ${\left(L_{t}\right)}_{t=0}^{T}$? Second, does the process have a tractable description?

We focus on the simplest situation, that in which the population process is deterministic. However, the results here apply when the population process solves either a nonlocal or a classical PDE. There will be no coalescence of ancestral lineages in the deterministic limit, but understanding motion of single lineages is useful in practice, and our results can be seen as a first step towards understanding genealogies for high population densities.

Proofs of results in this section are found in Section 8.

#### Definition 2.22 (Ancestral lineage)

*Let* ${\left(\varphi_{t}(x)\right)}_{0\leq t\leq T}$ *denote the density of the scaling limit of our population model, solving* (2.9), *and let* y *be a point with* $\varphi_{T}(y)>0$. *We define* ${\left(L_{s}\right)}_{s=0}^{T}$, *the ancestral lineage of an individual sampled from the population at* y *at time* T, *by setting* $L_{0}=y$ *and* $L_{s}$ *to be the position of the unique ancestor of that individual at time* T-s. *We define* ${\left(Q_{s}\right)}_{s\geq 0}$ *to be the time inhomogeneous semigroup satisfying*

$$Q_{s}f(y):=E_{y}\left[f\left(L_{s}\right)\right].$$

Our next result identifies the ancestral lineage as a diffusion by characterizing its generator.

#### Theorem 2.23

*For* $\varphi :R^{d}\to R$, *define*

(2.17) $${ℒ}_{\varphi }f\;=\frac{r}{\varphi }\left[{ℬ}^{*}(\gamma \varphi f)-f{ℬ}^{*}(\gamma \varphi )\right]$$

(2.18) $$=r\gamma \left[\sum_{ij}C_{ij}\partial_{x_{i}x_{j}}f+\sum_{j}{\vec{m}}_{j}\partial_{x_{j}}f\right],$$

where $\vec{m}$ is the vector

$${\vec{m}}_{j}=2\sum_{i}C_{ij}\partial_{x_{i}}\log(\gamma \varphi )+2\sum_{i}\partial_{x_{i}}C_{ij}-{\vec{b}}_{j}.$$

Then the generator of the semigroup $Q_{s}$ of Definition 2.22 is given by $\partial_{s}Q_{s}f(y)={ℒ}_{\varphi_{T-s}}Q_{s}f(y)$.

#### Remark 2.24

As usual, to make the generator readable, we’ve written it in concise notation, omitting the dependencies on location and population density, which itself changes with time. When interpreting this, remember that everything depends on location and density at that location and time – for instance, “r” is actually $r\left(x,\varphi (x)\right.$) (in the classical case), or $r\left(x,\rho_{r}*\right.\eta (x)$) (in the nonlocal case).

Moreover, we haven’t proved any regularity of the population density process φ, so, as written, the generator (2.17) may not make sense. Instead, it should be interpreted in a weak sense which is made precise in *Section 8.2.*

#### Corollary 2.25

*In addition to the assumptions of Theorem 2.23, if the covariance of the dispersal process is isotropic (i.e*., $C=\sigma^{2}I$), *then*

(2.19) $${ℒ}_{\varphi }f=r\gamma \left(\sigma^{2}\Delta f+\left(2\sigma^{2}\nabla \;log(\gamma \varphi )-\vec{b}\right)\cdot \nabla f\right).$$

(However, $\vec{b}$ can still depend on location.)

In other words, the lineage behaves as a diffusion driven by Brownian motion run at speed $\sigma^{2}$ multiplied by the local per-capita production of mature offspring (rγ) in a potential tilted by migration bias $({\left(\varphi_{s}\gamma \right)}^{2}exp(-\vec{b}\cdot x/\sigma^{2})$, whose gradient appears in the drift term of the generator). In particular, lineages are drawn to regions of high fecundity (production of juveniles), but their speed is determined by the rate of production of mature offspring. This can be compared to Remark 2.7.

#### Corollary 2.26

*In addition to the assumptions of* Corollary 2.25, *if the population process is stationary (so* $\varphi_{t}\equiv \varphi$), *and* $\vec{b}(x)=\nabla h(x)$ *for some function* h, *then* Y *is reversible with respect to*

(2.20) $$\pi (x)=\frac{\gamma }{r}\varphi (x{)}^{2}e^{-h(x)/\sigma^{2}}.$$

Long-term fitness of an individual is proportional to the fraction of lineages from the distant future that pass through the individual, and hence the total long-term fitness at a location is proportional to the stationary distribution of Y there, if it exists. Therefore, if π is integrable then the per-capita long-term fitness of an individual at x is proportional to π(x)/φ(x).

#### Corollary 2.27

In addition to the assumptions of Corollary 2.25, suppose that the population process is described by a travelling wave with velocity c, i.e., the population has density φ(t,x)=w(x-tc) where w solves

$$r{ℬ}^{*}(\gamma w)+wF+c\cdot \nabla w=r\sigma^{2}\Delta (\gamma w)-\vec{b}\cdot \nabla (\gamma w)+c\cdot \nabla w=0.$$

Then the semigroup $Q_{s}$ of the motion of a lineage in the frame that is moving at speed c is time-homogeneous with generator

(2.21) $$ℒf=\sigma^{2}r\gamma (\Delta f+2\nabla \;log(\gamma w)\cdot \nabla f)+(c-r\gamma \vec{b})\cdot \nabla f.$$

## 3 Examples and applications

We now discuss some consequences of these results.

### 3.1 Beyond linear diffusion

Equation (2.9) is a nonlocal version of a reaction-diffusion equation; the diffusion is nonlinear if γ depends on population density: in other words, if the diffusivity of the population depends on the population density. Passing to the classical limit, we recover equations like (2.14). Such equations are widely used in a number of contexts in biology in which motility within a population varies with population density. For example, density dependent dispersal is a common feature in spatial models in ecology, eukaryotic cell biology, and avascular tumour growth; see Sherratt [2010] and references therein for further discussion. In particular, it has been suggested as a model for the expansion of a certain type of bacteria on a thin layer of agar in a Petri dish [Cohen et al., 1999]. We shall pay particular attention to the case in which the equation can be thought of as modelling the density of an expanding population. We focus on the monostable reaction of (2.14).

**Figure 2:**
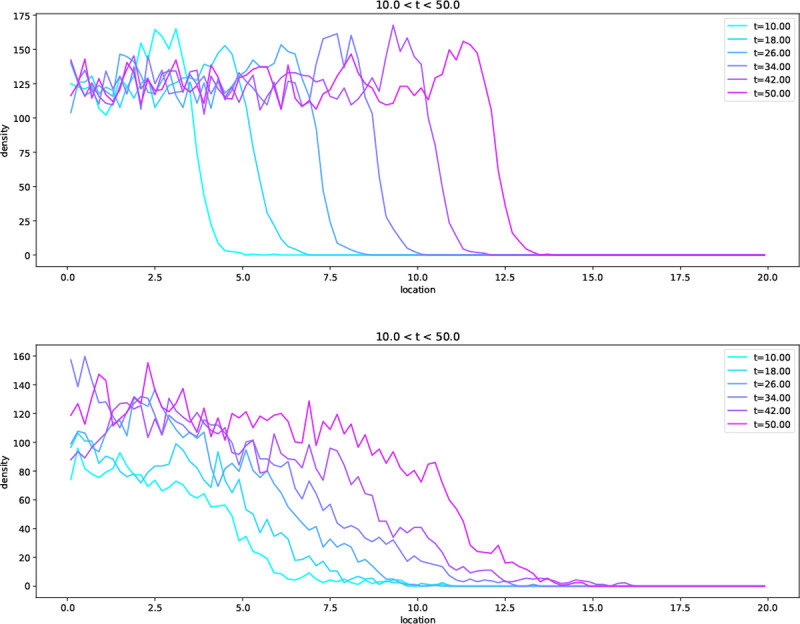
Simulated populations under a porous medium equation with logistic growth (2.14) in d=1,θ/N small on the left; large on the right. Values of θ in top and bottom figures are 1 and 100, respectively, and both have N set so that the density is roughly 100 individuals per unit of habitat (as displayed on the vertical axis). See text for details of the simulations.

Comparing with (2.9), we see that to set up a limit in which the population density φ follows the porous medium equation with logistic growth of (2.14), we need r=1,γ=φ, and F=1-φ. Consulting equation (2.2), this implies that $\mu_{\theta }=max(0,(1+1/\theta )\varphi -1/\theta )$. In other words, establishment is certain and birth rates increase linearly with population density, but to compensate, death rates increase slightly faster (also linearly). Alert readers will notice that the condition from Assumptions 2.8 that γ(x,m) be uniformly bounded is violated. This can be corrected by use of a cut-off, and in fact the downwards drift provided by the logistic control of the population size prevents m from getting too big. In practice the simulations shown in Figure 2 take discrete time steps of length dt (with dt suitably small), and have each individual reproduce and die with probabilities, respectively,

$$p_{\text{birth}}\left(m\right)=\left(1-e^{-mdt}\right)\;p_{\text{death}}\left(m\right)=\left(1-e^{-\left(m\left(1+1/\theta \right)-1/\theta \right)dt}\right),$$

where m is the local density at their location. This makes $\gamma (x,m)=p_{\text{birth}\;}(m)/dt\approx m$ and

F(x,m)=θ(γ(x,m)-μ(x,m))/dt≈1-m.

Birth and death rates are equal at density m=1, corresponding to an unscaled density of N individuals per unit area.

In one dimension, equation (2.14) has an explicit travelling wave solution

(3.1) $$w^{P}(t,x):={\left(1-e^{\frac{1}{2}\left(x-x_{0}-t\right)}\right)}_{+}.$$

Notice that the wave profile has a sharp boundary at $x=x_{0}+t$. There are also travelling wave solutions with c>1 [Gilding and Kersner, 2005], which lack this property. However, for initial conditions that decay sufficiently rapidly at infinity, such as one might use in modelling a population invading new territory, the solution converges to (3.1) [Kamin and Rosenau, 2004]. In Figure 2 we show simulations of the individual based model described above, which display travelling wave solutions qualitatively similar to solutions of (2.14), with better agreement for smaller θ/N (but in both cases, N is reasonably large).

### 3.2 Ancestry in different types of travelling waves

Although it remains challenging to establish the distribution of genealogical trees relating individuals sampled from our population model, as described in the introduction, we can gain some insight by investigating the motion of a single ancestral lineage. Here we do that in the context of a one-dimensional population expanding into new territory as a travelling wave. We focus on three cases in which we have explicit information about the shape of the travelling wave profile: the Fisher-KPP equation, a special case of the Allen-Cahn equation with a bistable nonlinearity, and the porous media equation with logistic growth, equation (2.14). We work here in one dimension, and take $\sigma^{2}=1$ and $\vec{b}=0$.

#### Fisher-KPP equation:

Consider the classical Fisher-KPP equation,

(3.2) $$\partial_{t}\varphi =\partial_{xx}\varphi +\varphi (1-\varphi ).$$

Even though we do not have an explicit formula for the wave shape in this case, our methods provide information about ancestral lineages. The equation has non-negative travelling wave solutions of speed c for all c≥2, but, started from any compact perturbation of a Heaviside function, the solution will converge to the profile $w^{F}$ with the minimal wavespeed, c=2 [Kolomogorov et al., 1937, Fife and McLeod, 1977, Bramson, 1983]. No matter what initial condition, for any t>0 the support of the solution will be the whole real line. In this case, we must have r=γ=1, and F(x,m)=1-m so $\mu_{\theta }(x,m)=1+(m-1)/\theta$. By Corollary 2.27, the generator of the motion of an ancestral lineage is

(3.3) $${ℒ}_{F}f=\partial_{xx}f+2\frac{\partial_{x}w^{F}}{w^{F}}\partial_{x}f+2\partial_{x}f.$$

Near the tip of the wave (for x large), $w^{F}(x)∼e^{-x}$, so (3.3) implies that the motion of a lineage is close to unbiased Brownian motion. On the other hand, in the “bulk”, a lineage behaves approximately as Brownian motion with drift at rate two to the right. This implies that ancestral lineages are pushed into the tip of the wave, and there is no stationary distribution, so that long-term dynamics of genetic inheritance depend on the part of the wave not well-approximated by a smooth profile, in agreement with the previous results referred to in the Introduction.

#### Allen-Cahn equation:

Now take the Allen-Cahn equation:

(3.4) $$\partial_{t}\varphi =\partial_{xx}\varphi +\varphi \left(1-\varphi \right)\left(2\varphi -1+s\right),$$

for a given s∈(0,2). Once again we have taken r=γ=1, but now the reaction term F(x,m)=(1-m)(2m-1+s) is bistable. This equation can be used to model the motion of so-called hybrid zones in population genetics; see, for example, Barton [1979], Gooding [2018], and Etheridge et al. [2022]. This equation has an explicit travelling wave solution with speed s and shape

$$w^{A}(x)={\left(1+e^{x}\right)}^{-1},$$

i.e., $\phi_{t}(x)=w^{A}(x-st)$ solves (3.4). Substituting $w^{A}$ in place of $w^{F}$ in (3.3), we find that the generator of an ancestral lineage relative to the wavefront is now,

$${ℒ}_{A}f\;=\partial_{xx}f+2\frac{\partial_{x}w^{A}}{w^{A}}\partial_{x}f+s\partial_{x}f\;\;=\partial_{xx}f-2\frac{e^{x}}{1+e^{x}}\partial_{x}f+s\partial_{x}f,$$

so lineages in the tip are pushed leftwards into the bulk of the wave at a rate $s-2e^{x}/\left(1+e^{x}\right)$. The density of the speed measure for this diffusion is

$$m_{A}(x)\propto e^{sx}{\left(1+e^{x}\right)}^{-2},$$

which is integrable, and so determines the unique stationary distribution. Thus the position of the ancestral lineage relative to the wavefront will converge to a stationary distribution which is maximised away from the extreme tip of the wave. This is consistent with Etheridge and Penington [2022], who consider an analogous stochastic population model, although the stronger result there (that the genealogy of a sample from behind the wavefront is approximately a Kingman coalecsent) requires the stronger condition s<1.

#### Porous Medium equation with logistic growth:

Finally, consider equation (2.14). Setting $x_{0}=0$ (for definiteness) and substituting the form of $w^{P}$ from equation (3.1) into Corollary 2.27, with c=1,γ(x,m)=m,r(x,w)=1, and F(x,m)=(1-m), the generator of the diffusion governing the position of the ancestral lineage relative to the wavefront is, for x<0,

$${ℒ}_{P}f\;=w^{P}\left(\partial_{xx}f+2\frac{\partial_{x}\left({\left(w^{P}\right)}^{2}\right)}{{\left(w^{P}\right)}^{2}}\partial_{x}f\right)+\partial_{x}f\;\;=\left(1-e^{\frac{1}{2}x}\right)\partial_{xx}f-2e^{\frac{1}{2}x}\partial_{x}f+\partial_{x}f.$$

The speed measure corresponding to this diffusion has density

$$m_{P}(\xi )\;\propto \frac{1}{2\left(1-e^{\xi /2}\right)}exp\left(\int_{\eta }^{\xi }\left\{1-\frac{e^{x/2}}{1-e^{x/2}}\right\}dx\right)\;\;\propto e^{\xi }\left(1-e^{\xi /2}\right),\;\;\text{for}\;\xi <0$$

and $m_{P}(\xi )=0$ for ξ≥0, which is integrable and so when suitably normalised gives the unique stationary distribution. Notice that even though we have the same reaction term as in the Fisher-KPP equation, with this form of nonlinear diffusion, at stationarity the lineage will typically be significantly behind the front, suggesting a different genealogy.

It is interesting to compare the stationary distribution we have obtained here to the expression that we’d get by setting $\vec{b}=-c$ and using Corollary 2.26, i.e., by giving each offspring a mean displacement that offsets the motion of the wave. In the Fisher-KPP and Allen-Cahn cases above, we get the same expressions, but this is only the case because r≡γ≡1 in both. For the PME with $\vec{b}=1$ we have ${ℬ}^{*}=\Delta +\nabla$ and so the equation solved by the population density is

$$\partial_{t}\varphi =\Delta \varphi +2\varphi \nabla \varphi +\varphi (1-\varphi ),$$

which has a traveling wave solution of the same shape but moving at half the speed, $\varphi (x,t)=w^{P}(x-t/2)$, and a stationary distribution of the lineage relative to the wavefront of $\pi (\xi )\propto e^{3\xi /2}{\left(1-e^{\xi /2}\right)}^{3}$.

### 3.3 Clumping from nonlocal interactions

**Figure 3:**
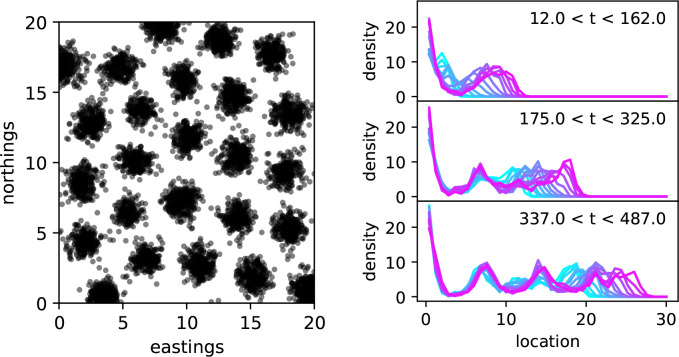
**Left:** A snapshot of individual locations in a two-dimensional simulation in which the constant density is unstable and a stable, periodic pattern forms. **Right:** Population density in an expanding wave in a one-dimensional simulation forming a periodic pattern; each panel shows the wavefront in three periods of time; within each period of time the wavefront at earlier times is shown in blue and later times in pink. In both cases, γ(m)=3/(1+m), μ≡0.3, and r≡1; dispersal is Gaussian with σ=0.2 and density is measured with $\rho_{\gamma }(x)=p_{9}(x)$, i.e., using a Gaussian kernel with standard deviation 3.

Simulating these processes and exploring parameter space, one sooner or later comes upon a strange observation: with certain parameter combinations, the population spontaneously forms a regular grid of stable, more or less discrete patches, separated by areas with nearly no individuals, as shown in Figure 3. The phenomenon is discussed in Section 16.10 of Haller and Messer [2022], and has been described in similar models, e.g., by Britton [1990], Sasaki [1997], Hernández-García and López [2004], Young et al. [2001], and Berestycki et al. [2009]. For example, if the density-dependent effects of individuals extend farther (but not too much farther) than the typical dispersal distance, then depending on the interaction kernel new offspring landing between two clumps can effectively find themselves in competition with *both* neighbouring clumps, while individuals within a clump compete with only one.

More mathematically, consider the case in which $ℬ=\sigma^{2}\Delta$ and all parameters are spatially homogeneous, so that $r(x,\eta )=r\left(\rho_{r}*\eta (x)\right)$, and similarly for γ and F. If $\varphi_{0}$ is such that $F\left(\varphi_{0}\right)=0$ and $F^{\prime }\left(\varphi_{0}\right)<0$, then the constant solution $\varphi \equiv \varphi_{0}$ is a nontrivial equilibrium of (1.1). However, this constant solution may not be unique, it may be unstable, and a stable solution may have oscillations on a scale determined by the interaction distance.

To understand the stability of the constant solution $\varphi \equiv \varphi_{0}$, we linearise (1.1) around $\varphi_{0}$ : let $\varphi_{t}(x)=\varphi_{0}+\psi_{t}(x)$, and (informally) $r(x)\approx r\left(\varphi_{0}\right)+r^{\prime }\left(\varphi_{0}\right)\rho_{r}*\psi (x)$. Writing $r_{0}=r\left(\varphi_{0}\right)$ and $r_{0}^{\prime }=r^{\prime }\left(\varphi_{0}\right)$, with analogous expressions for γ and F,

$$\partial_{t}\psi \approx \sigma^{2}\varphi_{0}r_{0}\gamma_{0}^{\prime }\Delta \rho_{\gamma }*\psi +\sigma^{2}r_{0}\gamma_{0}\Delta \psi +\varphi_{0}F_{0}^{\prime }\rho_{F}*\psi .$$

Letting $\hat{f}(u)=\int e^{2\pi iux}f(x)dx$ denote the Fourier transform,

(3.5) $$\partial_{t}\hat{\psi }\left(u\right)\approx \left\{-u^{2}\sigma^{2}\varphi_{0}r_{0}\gamma_{0}^{\prime }{\hat{\rho }}_{\gamma }\left(u\right)-u^{2}\sigma^{2}r_{0}\gamma_{0}+\varphi_{0}F_{0}^{\prime }{\hat{\rho }}_{F}\left(u\right)\right\}\hat{\psi }\left(u\right).$$

In the simplest case, in which γ is constant, so $\gamma_{0}^{\prime }=0$, this reduces to

(3.6) $$\partial_{t}\hat{\psi }(u)\approx \left(-u^{2}\sigma^{2}r_{0}\gamma_{0}+\varphi_{0}F_{0}^{\prime }{\hat{\rho }}_{F}(u)\right)\hat{\psi }(u).$$

If we take $\rho_{F}=p_{\epsilon^{2}}$, then ${\hat{\rho }}_{F}(u)=exp\left(-2\pi \epsilon^{2}u^{2}\right)$ and (recalling that $F_{0}^{\prime }<0$) the term in brackets is always negative, and we recover the well-known fact that in this case the constant solution is stable. If, on the other hand, ${\hat{\rho }}_{F}$ changes sign, there may be values of u for which the corresponding quantity is positive. For example, if d=1 and $\rho_{F}(x)=1_{\left[-\epsilon ,\epsilon \right]}(x)/2\epsilon$, then ${\hat{\rho }}_{F}(u)=sin(2\pi \epsilon u)/(2\pi \epsilon u)$, which is negative for u∈(1/(2ϵ),1/ϵ) (and periodically repeating intervals). Setting v=ϵu, the bracketed term on the right hand side of (3.6) becomes

$$\varphi_{0}F_{0}^{\prime }\frac{1}{2\pi v}\sin\left(2\pi v\right)-\frac{\sigma^{2}}{\epsilon^{2}}v^{2}r_{0}\gamma_{0},$$

and we see that if $\sigma^{2}/\epsilon^{2}$ is sufficiently small, there are values of v for which this is positive. In other words, in keeping with our heuristic above, if dispersal is sufficiently short range relative to the range over which individuals interact, there are unstable frequencies that scale with the interaction distance ϵ. In two dimensions, replacing the indicator of an interval by that of a ball of radius ϵ, a similar analysis applies, except that the sine function is replaced by a Bessel function.

Now suppose that γ is not constant. Then, from (3.5), if we take $\rho_{\gamma }=\rho_{F}=p_{\epsilon }^{2}$,

$$\partial_{t}\hat{\psi }\left(u\right)=e^{-2\pi^{2}\epsilon^{2}u^{2}}\left\{-\sigma^{2}\varphi_{0}r_{0}\gamma_{0}^{\prime }u^{2}-\sigma^{2}r_{0}\gamma_{0}u^{2}e^{2\pi^{2}\epsilon^{2}u^{2}}+\varphi_{0}F_{0}^{\prime }\right\}\hat{\psi }\left(u\right).$$

If we make the (reasonable) assumption that $\gamma_{0}^{\prime }<0$, then we see that even when the Fourier transform of ρ does not change sign, there may be parameter values for which the constant solution is unstable. As before, we set v=ϵu. The term in brackets becomes

$$\frac{\sigma^{2}}{\epsilon^{2}}v^{2}r_{0}\left(-\varphi_{0}\gamma_{0}^{\prime }-\gamma_{0}e^{2\pi^{2}v^{2}}\right)+\varphi_{0}F_{0}^{\prime },$$

and, provided $-\varphi_{0}\gamma_{0}^{\prime }/\gamma_{0}>1$, for sufficiently small v the term in round brackets is positive. We now see that if $\sigma^{2}/\epsilon^{2}$ is sufficiently large, the equilibrium state $\varphi \equiv \varphi_{0}$ is unstable. As before, the unstable frequencies will scale with ϵ and for given F,r and γ, whether or not such unstable frequencies exist will be determined by $\sigma^{2}/\epsilon^{2}$, but in this case of Gaussian kernels, it is interaction distance being sufficiently small relative to dispersal that will lead to instability.

### 3.4 Lineage motion is not uniquely determined by population density

It is natural for applications to wonder about identifiability: when can the observed quantities like population density or certain summaries of lineage movement uniquely determine the underlying demographic parameters? Consider a deterministic, continuous population generated by parameters γ,r, and F, with $\vec{b}=0$ and C=I. Suppose it has a stationary profile w(x), that must satisfy

rΔ(γw)+Fw=0.

It is easy to see that w does not uniquely specify γ,F, and r: let λ(x) be a smooth, nonnegative function on $R^{d}$, and let $\tilde{r}(x,m)=\lambda (x)r(x,m)$ and $\tilde{F}(x,m)=\lambda (x)F(x,m)$ (and, let $\tilde{\gamma }=\gamma$). Since μ=rγ-F/θ, this corresponds to multiplying both establishment probabilities and death rates by λ. Then the population with parameters $\tilde{\gamma },\;\tilde{r}$, and $\tilde{F}$ has the same stationary profile(s) as the original population.

Can these two situations be distinguished from summaries of lineage movement? The first has lineage generator

f↦ℒf=rγ(Δf+2∇log(γw)⋅∇f),

while the second has lineage generator f↦λ(x)ℒf(x). In other words, although the stationary profile of the population is unchanged when we scale local establishment and death by λ, the motion of lineages is sped up locally by λ. This corresponds to making areas with λ>1 more “sink-like” and λ<1 more “source-like”: if λ(x)>1, then at x both the death rate and probability of establishment of new individuals are higher. As a result, lineages in the second model spend more time in areas with λ<1, i.e., those areas have higher reproductive value, something that is, in principle, discernible from genetic data (because, for instance, making reproductive value less evenly distributed reduces long-term genetic diversity).

## 4 Heuristics

In this section we perform some preliminary calculations and use them to provide heuristic arguments for our main results, to build intuition before the formal proofs.

### 4.1 The population density

*We reiterate that in our prelimiting model, the population is represented by a point measure* $\eta^{N}$ *in which each individual is assigned a mass* 1/N. *We use the term “population density” for this process, as it is supposed to measure population size relative to a nominal occupancy of* N *individuals per unit area, but it is not absolutely continuous with respect to Lebesgue measure*.

We write ${𝒫}^{N}$ for the generator of the scaled population process $\eta^{N}$ of Definition 2.4 acting on test functions of the form G(⟨f,η⟩), where f≥0 is smooth and bounded on $R^{d}$ and $G\in C^{\infty }([0,\infty ))$. Recall that θ=θ(N)→∞ as N→∞ in such a way that θ(N)/N→α.

A Taylor expansion allows us to write

(4.1) $${𝒫}^{N}G(\langle f,\eta \rangle )=G^{\prime }(\langle f,\eta \rangle )\underset{\delta t↓0}{lim}\;\frac{1}{\delta t}E\left[\left\langle f,\eta_{\delta t}\right\rangle -\langle f,\eta \rangle |\eta_{0}=\eta \right]\;\;+\frac{1}{2}G^{\prime \prime }(\langle f,\eta \rangle )\underset{\delta t↓0}{lim}\;\frac{1}{\delta t}E\left[{\left(\left\langle f,\eta_{\delta t}\right\rangle -\langle f,\eta \rangle \right)}^{2}|\eta_{0}=\eta \right]+\epsilon_{N}(f,G,\eta ),$$

where the terms that make up $\epsilon_{N}(f,G,\eta )$ will be negligible in our scaling limit (at least if $G^{\prime \prime \prime }<\infty$).

### Mean measure

Recall that in our parameterization only death rates $\mu_{\theta }$ and the dispersal kernel $q_{\theta }$ depend on θ. For a suitable test function f, we find

(4.2) $${𝒫}^{N}\langle f,\eta \rangle \;=\underset{\delta t↓0}{lim}\;\frac{1}{\delta t}E\left[\left\langle f,\eta_{\delta t}\right\rangle -\langle f,\eta \rangle |\eta_{0}=\eta \right]\;\;=\theta \int \int f\left(z\right)r\left(z,\eta \right)q_{\theta }\left(x,dz\right)\gamma \left(x,\eta \right)\eta \left(dx\right)-\theta \int f\left(x\right)\mu_{\theta }\left(x,\eta \right)\eta \left(dx\right).$$

The first term is the increment in ⟨f,η⟩ resulting from a birth event (recalling that we don’t kill the parent) integrated against the rate of such events, and the second reflects death events. The factor of θ appears from the time rescaling. In both terms, the rate of events has a factor of N (because events happen at a rate proportional to the number of individuals, whereas η has mass 1/N for each individual) which is offset by the fact that the birth or loss of a single individual at the point y, say, changes ⟨f,η⟩ by f(y)/N.

We use the fact that $\int q_{\theta }(x,dz)=1$ to rewrite (4.2) as

(4.3) $$\int (\int \theta (f(z)r\left(z,\eta )-f(x)r(x,\eta ))q_{\theta }(x,dz\right))\gamma (x,\eta )\eta (dx)\;\;+\int f\left(x\right)\theta (r\left(x,\eta \right)\gamma \left(x,\eta \right)-\mu_{\theta }\left(x,\eta \right))\eta \left(dx\right).$$

We have defined $\mu_{\theta }$ so that the second term is simple:

$$\theta (r(x,\eta )\gamma (x,\eta )-\mu_{\theta }(x,\eta ))=F\left(x,\eta \right).$$

Furthermore, recall from Remark 2.6 that

(4.4) $$\int \theta (r(z,\eta )f(z)-r(x,\eta )f(x))q_{\theta }(x,dz)\;\overset{\theta \to \infty }{⟶}\;ℬ(r(\cdot ,\eta )f(\cdot ))(x).$$

In particular, if dispersal is determined by a standard multivariate Gaussian with mean zero and covariance $\sigma^{2}I/\theta$, then $ℬ=\sigma^{2}\Delta$, where Δ denotes the Laplacian.

In summary, equation (4.3) converges to

(4.5) $$\int \gamma \left(x,\eta \right)ℬ\left(f\left(\cdot \right)r\left(\cdot ,\eta \right)\right)\left(x\right)\eta \left(dx\right)+\int f\left(x\right)F\left(x,\eta \right)\eta \left(dx\right),$$

which explains the form of the martingale of Theorem 2.10.

### Quadratic variation

We now look at the second order term in (4.1), which will converge to the quadratic variation of the limiting process. An individual at location x gives birth to a surviving offspring at y at rate

$$\gamma (x,\eta )r(y,\eta )q_{\theta }(x,dy),$$

and since this increments ⟨f,η⟩ by f(y)/N, the contribution to the quadratic variation from birth events, which occur at rate θ per individual (so, rate Nθ|η| overall), is

$$\int N\theta \gamma \left(x,\eta \right)\int \frac{1}{N^{2}}f^{2}\left(y\right)r\left(y,\eta \right)q_{\theta }\left(x,dy\right)\eta \left(dx\right).$$

Similarly, the increment in ⟨f,η⟩ resulting from the death of an individual at x is -f(x)/N, and so combining with the above, the second order term in the generator takes the form

$$G^{\prime \prime }(\langle f,\eta \rangle )\frac{1}{2}N\theta \left\{\int \gamma (x,\eta )\int \frac{1}{N^{2}}f^{2}(y)r(y,\eta )q_{\theta }(x,dy)\eta (dx)+\int \mu_{\theta }(x,\eta )\frac{1}{N^{2}}f^{2}(x)\eta (dx)\right\}\;\;=\frac{1}{2}G^{\prime \prime }(\langle f,\eta \rangle )\frac{\theta }{N}\int \left\{\gamma (x,\eta )\int f^{2}(y)r(y,\eta )q_{\theta }(x,dy)+f^{2}(x)\mu_{\theta }(x,\eta )\right\}\eta (dx).$$

Since $\int f^{2}(y)r(y,\eta )q_{\theta }(x,dy)\to f^{2}(x)r(x,\eta )$ and $r\gamma +\mu_{\theta }=2r\gamma -F/\theta \to 2r\gamma$ as θ→∞, this converges to

$$\frac{\alpha }{2}G^{\prime \prime }\left(\left\langle f,\eta \right\rangle \right)\left\langle 2r\left(x,\eta \right)\gamma \left(x,\eta \right),\eta \left(dx\right)\right\rangle .$$

An entirely analogous argument shows that if $G^{\prime \prime \prime }$ is bounded, then the term $\epsilon_{\theta ,N}(f,G,\eta )$ in (4.1) will be $𝒪\left(\theta /N^{2}\right)$.

If we hold $\rho_{\gamma },\;\rho_{r},\;\rho_{F}$ fixed, then by taking θ/N→0, the second order term in the generator will vanish and we expect a deterministic limit, for which $\partial_{t}\left\langle f,\eta_{t}\right\rangle$ is equal to (4.5). In other words, the limit is a weak solution to the deterministic equation

(4.6) $$\partial_{t}\varphi_{t}(x)=r\left(x,\varphi_{t}\right)ℬ\left(\gamma \left(\cdot ,\varphi_{t}\right)\varphi_{t}(\cdot )\right)(x)+F\left(x,\varphi_{t}\right)\varphi_{t}(x)$$

in the sense of Definition 2.12, where $\varphi_{t}$ is the density of $\eta_{t}$, if it has a density. On the other hand, if N=αθ for some α>0, the second order term remains, and we expect a “generalised superprocess” limit. The limiting quadratic variation is exactly as seen in Theorem 2.10.

#### One-step convergence:

In order to pass directly to a classical PDE limit in Theorem 2.20 we impose the stronger condition that $\theta /N\epsilon^{d}\to 0$ and also require that $\theta \epsilon^{2}\to \infty$. Recall that in this case, we take $\rho_{F}^{\epsilon }$ to be a symmetric Gaussian density with variance $\epsilon^{2}$. The condition $\theta \epsilon^{2}\to \infty$ ensures that $\epsilon^{2}$ is large enough relative to 1/θ that the regularity gained by smoothing our population density by convolution with $\rho_{\epsilon }$ is preserved under the dynamics dictated by $q_{\theta }$. To understand the first condition, note that we are aiming to obtain a deterministic expression for the limiting population density. It is helpful to think about a classical Wright-Fisher model (with no spatial structure and just two types, say). We know then that if the timescale θ is on the same order as population size N, we see stochastic fluctuations in the frequencies of the two types in the limit as N→∞; to obtain a deterministic limit, we look over timescales that are short relative to population size. In our setting, the total population size is replaced by the local population size, as measured by convolution with $\rho_{\epsilon }$, which we expect to be of order $N\epsilon^{d}$, and so in order to ensure a deterministic limit we take $\theta /\left(N\epsilon^{d}\right)\to 0.$

### 4.2 Motion of ancestral lineages

Although our proof of Theorem 2.23 uses an explicit representation in terms of the lookdown process, the result can be understood through informal calculations. Suppose that we have traced a lineage back to an individual at location y at time t. Looking further back through time, at the time of the birth of that individual, the lineage will jump to the location of the parent of the individual. Now, the rate at which new individuals are born to parents at x and establish at y is

$$\theta N\eta_{t}^{N}\left(dx\right)\gamma \left(x,\eta_{t}^{N}\right)q_{\theta }\left(x,dy\right)r\left(y,\eta_{t}^{N}\right).$$

Suppose that $\eta^{N}$ did have a density (in the prelimit it does not), say $\eta_{t}^{N}(dx)=\varphi_{t}^{N}(x)dx$. Informally, since the number of individuals near y is $N\varphi_{t}^{N}(y)dy$, the probability that a randomly chosen individual near y is a new offspring from a parent at x in [t,t+dt) is

(4.7) $$\frac{\theta \varphi_{t}^{N}(x)\gamma \left(x,\eta_{t}^{N}\right)r\left(y,\eta_{t}^{N}\right)}{\varphi_{t}^{N}(y)}\frac{q_{\theta }(x,dy)}{dy}dxdt.$$

Leaving aside questions of whether a lineage can be treated as a randomly chosen individual, we define a continuous-time jump process whose transition rates, conditional on ${\left(\varphi_{t}^{N}\right)}_{t=0}^{T}$, are given by (4.7). Because we are tracing the lineage backwards in time we make the substitution s=T-t and write ${\left(L_{s}^{N}\right)}_{s=0}^{T}$ for the location of a lineage that moves according to these jump rates. Then, abusing notation to write $q_{\theta }(x,y)$ for the density of $q_{\theta }(x,dy)$,

(4.8) $$E\left[f\left(L_{s+ds}^{N}\right)-f(y)|L_{s}^{N}=y\right]\;\;=ds\;\theta \int (f(x)-f(y))\frac{\varphi_{T-s}^{N}(x)\gamma \left(x,\eta_{T-s}^{N}\right)r\left(y,\eta_{T-s}^{N}\right)}{\varphi_{T-s}^{N}(y)}q_{\theta }(x,y)dx.$$

(Note that this integral is with respect to x.) Referring back to Remark 2.6, a quick calculation shows that as N→∞,

$$\theta \int \;(f(x)-f(y))g(x)q_{\theta }(x,y)dx\;\;=\theta \int \{(f(x)g(x)-f(y)g(y))-f(y)(g(x)-g(y))\}q_{\theta }(x,y)dx\;\;\to {ℬ}^{*}\left(fg\right)\left(y\right)-f\left(y\right){ℬ}^{*}g\left(y\right).$$

Applying this to (4.8) with $g=\varphi_{T-s}\gamma$, this suggests that the generator of the limiting process is

(4.9) $${ℒ}_{s}f=\frac{r}{\varphi_{T-s}}\left\{{ℬ}^{*}\left(\gamma \varphi_{T-s}f\right)-f{ℬ}^{*}\left(\gamma \varphi_{T-s}\right)\right\}.$$

This agrees with Theorem 2.23.

## 5 The lookdown process

Our characterisation of the motion of lines of descent (from which we establish that of ancestral lineages) when we pass to the scaling limit in our model will be justified via a lookdown construction. In this section we present such a construction for the general population model of Definition 2.4. It will be in the spirit of Kurtz and Rodrigues [2011]. The general set-up is as follows. Each individual will be labelled with a “level”, a number in [0,N]. We will still encode the process embellished by these levels as a point measure: if the *i*^th^ individual’s spatial location is $x_{i}$ and level is $u_{i}$, then we will write

$$\xi^{N}=\sum_{i}\delta_{x_{i},u_{i}},$$

which is a measure on $R^{d}\times [0,N]$. Note that each individual contributes mass 1 to the measure, not 1/N as above. If we assign mass 1/N to each individual and ignore the levels we will recover our population model. Moreover, at any time, the levels of individuals in a given spatial region will be exchangeable and conditionally uniform on [0,N]: in particular, choosing the k individuals with the lowest levels in that region is equivalent to taking a uniform random sample of size k from the population in the region. However, this exchangeability is only as regards the *past*: an individual’s level encodes information about their future reproductive output, since individuals with lower levels tend to live longer, and have more offspring. For more explanation of the set-up and how this is possible, see Kurtz and Rodrigues [2011] and Etheridge and Kurtz [2019] (and note that our N corresponds to the λ of those papers). The power of this approach is that we can pass to a limit under the same scalings as described in Theorem 2.10, and the limiting “spatial-level” process will still be a point measure, and so we explicitly retain the notion of individuals and lineages in the infinite-population limit.

### 5.1 Lookdown representation of the model of Definition 2.4

For the remainder of this section, when there is no risk of ambiguity we shall suppress the superscript N on the processes η and ξ.

In this subsection, we’ll define the process ${\left(\xi_{t}\right)}_{t\geq 0}$ in terms of the dynamics of labelled particles, and write down its generator. The dynamics depend on the spatial locations of particles, and in this section $\eta_{t}$ is the corresponding spatial measure, i.e.,

$$\eta_{t}(\cdot )=\frac{1}{N}\xi_{t}(\cdot \times [0,N]).$$

A nontrivial consequence of the way we define $\xi_{t}$ will be that this process has the same distribution as the process ${\left(\eta_{t}\right)}_{t\geq 0}$ of Definition 2.4. (This provides our justification for using the same notation for both.)

Following Etheridge and Kurtz [2019], we build the generator step by step from its component parts. Suppose that the initial population is composed of O(N) particles with levels uniformly distributed on [0,N], and that the current state of the population is ξ, with spatial projection η.

An individual at spatial location x with level u produces one juvenile offspring at rate

$$2\theta \left(1-\frac{u}{N}\right)\gamma \left(x,\eta \right),$$

which disperses to a location relative to x drawn from the kernel $q_{\theta }(x,\cdot )$. Averaging over the uniform distribution of the level u, we recover the birth rate θγ(x,η). This juvenile – suppose its location is y – either survives, with probability r(y,η), or immediately dies. (As before, “maturity” is instantaneous.) If it survives, a new level $u_{1}$ is sampled independently and uniformly from [u,N], and the parent and the offspring are assigned in random order to the levels $\left\{u,u_{1}\right\}$. This random assignment of levels to parent and offspring will ensure that assignment of individuals to levels remains exchangeable.

Evidently this mechanism increases the proportion of individuals with higher levels. To restore the property that the distribution of levels is conditionally uniform given η, we impose that the level v of an individual at location x evolves according to the differential equation

$$\dot{v}=-\theta \frac{v}{N}(N-v)\gamma (x,\eta )\int_{R^{d}}\;r(y,\eta )q_{\theta }(x,dy).$$

Since v∈[0,N], this moves levels down; see Etheridge and Kurtz [2019], Section 3.4 for a detailed explanation.

Levels never cross below 0, while particles whose levels move above N are regarded as dead (and are removed from the population). Therefore, in order to incorporate death, the level of the individual at location x with level u moves upwards at an additional rate $\theta \mu_{\theta }(x,\eta )u$. Since levels are uniform, it is easy to check that if $\mu_{\theta }$ were constant, this would imply an exponential lifetime for each individual; see Etheridge and Kurtz [2019], Section 3.1 for more general justification.

Putting these together, the level u of an individual at x evolves according to:

(5.1) $$\dot{u}=-\theta \frac{u}{N}\left(N-u\right)\gamma \left(x,\eta \right)\int_{R^{d}}\;r\left(y,\eta \right)q_{\theta }\left(x,dy\right)+\theta \mu_{\theta }\left(x,\eta \right)u.$$

We shall write

$$b_{\theta }\left(x,\eta \right):=\theta \left(\gamma \left(x,\eta \right)\int_{R^{d}}r\left(y,\eta \right)q_{\theta }\left(x,dy\right)-\mu_{\theta }\left(x,\eta \right)\right),$$

which captures the local net difference between reproduction and death, and

(5.2) $$c_{\theta }(x,\eta ):=\frac{\theta }{N}\gamma (x,\eta )\int_{R^{d}}r(y,\eta )q_{\theta }(x,dy),$$

which captures the local rate of production of successful offspring. Recall from equation (2.2) that $F(x,\eta )=\theta \left(r(x,\eta )\gamma (x,\eta )-\mu_{\theta }(x,\eta )\right)$, and so

(5.3) $$b_{\theta }(x,\eta )=\theta \gamma (x,\eta )\int_{R^{d}}(r(y,\eta )-r(x,\eta ))q_{\theta }(x,dy)+F(x,\eta ).$$

Under Assumptions 2.8, as θ→∞ and $c_{\theta }(x,\eta )$ will tend to αγ(x,η)r(x,η)

(5.4) $$b_{\theta }(x,\eta )\to \gamma (x,\eta )ℬr(x,\eta )+F(x,\eta ).$$

We can then rewrite the differential equation governing the dynamics of the level of each individual as

(5.5) $$\dot{u}\;=\theta \gamma (x,\eta )\int_{R^{d}}\;r(y,\eta )q_{\theta }(x,dy)\left\{-\frac{u}{N}(N-u)+u\right\}-b_{\theta }(x,\eta )u\;\;=c_{\theta }\left(x,\eta \right)u^{2}-b_{\theta }\left(x,\eta \right)u.$$

Now, we can write down the generator for ${\left(\xi_{t}\right)}_{t\geq 0}$, the lookdown process. In what follows, we will write sums (and, products) over “(x,u)∈ξ” to mean a sum over the (location, level) pairs of each individual in the population. Test functions for ξ will take the form

(5.6) $$f(\xi )=\prod_{(x,u)\in \xi }g(x,u)=\exp(\int \log\;g(x,u)\xi (dx,du)),$$

where g(x,u) is differentiable in u and smooth in x. We will also assume that 0≤g(x,u)≤1 for all u∈[0,N], and g(x,u)≡1 for u≥N. In the expressions that follow, we shall often see one or more factor of 1/g(x,u); it should be understood that if g(x,u)=0, then it simply cancels the corresponding factor in f(ξ).

First consider the terms in the generator that come from birth events. When a birth successfully establishes, a new level is generated above the parent’s level, and this new level is assigned to either the offspring or the parent. Since the probability of each is 1/2, the contribution of birth to the generator maps f(ξ) to

(5.7) $$f(\xi )\sum_{(x,u)\in \xi }2\frac{\theta }{N}\gamma (x,\eta )\int_{u}^{N}\int_{{\mathbb{R}}^{d}}\left(\frac{1}{2}\left\{g\left(y,u_{1}\right)+\frac{g(y,u)g\left(x,u_{1}\right)}{g(x,u)}\right\}-1\right)r(y,\eta )q_{\theta }(x,dy)du_{1}$$

(5.8) $$=f(\xi )\sum_{(x,u)\in \xi }2\gamma (x,\eta )\{\frac{1}{2N}\int_{u}^{N}g\left(x,u_{1}\right)du_{1}\frac{\theta \int_{{\mathbb{R}}^{d}}(g(y,u)-g(x,u))r(y,\eta )q_{\theta }(x,dy)}{g(x,u)}\;\;\;+\frac{\theta }{N}\int_{u}^{N}\int_{{\mathbb{R}}^{d}}\left(\frac{g\left(y,u_{1}\right)+g\left(x,u_{1}\right)}{2}-1\right)r(y,\eta )q_{\theta }(x,dy)du_{1}\}.$$

In (5.7), $u_{1}$ is the new level and y is the offspring’s location, and so the two terms in the integral correspond to the two situations: in the first, we have added an individual at $\left(y,u_{1}\right)$, while in the second, we replace an individual at (x,u) by one at $\left(x,u_{1}\right)$ and another at (y,u). We’ve rewritten it in the form (5.8) because each of the two pieces naturally converges to a separate term in the limit.

The remaining term in the generator is due to the motion of particles’ levels. Reading off from (5.5), it takes the form

(5.9) $$f\left(\xi \right)\sum_{\left(x,u\right)\in \xi }\left(c_{\theta }\left(x,\eta \right)u^{2}-b_{\theta }\left(x,\eta \right)u\right)\frac{\partial_{u}g\left(x,u\right)}{g\left(x,u\right)}.$$

We can now define the spatial-level process explicitly as a solution to a martingale problem, whose generator is just the sum of (5.8) and (5.9). We need some notation. Write $𝒞=𝒞\left(R^{d}\times [0,\infty )\right)$ for the counting measures on $R^{d}\times [0,\infty )$ and ${𝒞}_{N}$ for the subset consisting of counting measures on $R^{d}\times [0,N]$.

#### Definition 5.1 (Martingale Problem Characterisation)

For given positive values of N and θ, define the generator $A^{N}$ by

(5.10) $$A^{N}f(\xi )\;\;\;\;\;=f(\xi )\sum_{(x,u)\in \xi }2\gamma (x,\eta )\{\frac{1}{2N}\int_{u}^{N}g\left(x,u_{1}\right)du_{1}\frac{\theta \int_{{\mathbb{R}}^{d}}(g(y,u)-g(x,u))r(y,\eta )q_{\theta }(x,dy)}{g(x,u)}\;\;\;\;\;\;\;\;\;\;\;\;\;\;\;\;\;\;\;\;\;\;\;\;\;\;\;\;\;\;\;\;\;\;\;\;\;\;\;\;\;\;\;\;\;\;\;\;\;\;\;\;\;\;\;\;\;\;\;\;\;\;\;\;\;\;\;\;\;+\frac{\theta }{N}\int_{u}^{N}\int_{{\mathbb{R}}^{d}}\left(\frac{g\left(y,u_{1}\right)+g\left(x,u_{1}\right)}{2}-1\right)r(y,\eta )q_{\theta }(x,dy)du_{1}\}\;\;\;\;\;\;\;\;\;\;\;\;\;\;\;\;\;\;\;\;\;\;\;\;\;\;\;\;\;\;\;\;\;\;\;\;\;\;\;\;\;\;\;\;+f(\xi )\sum_{(x,u)\in \xi }\left(c_{\theta }(x,\eta )u^{2}-b_{\theta }(x,\eta )u\right)\frac{\partial_{u}g(x,u)}{g(x,u)},$$

*where* $f(\xi )=\prod_{(x,u)\in \xi }\;g(x,u)$ *is as defined in* (5.6), *and* η(⋅)=ξ(⋅×[0,N])/N *as before*. *Given* $\xi_{0}\in {𝒞}_{N}$, *we say that a* ${𝒟}_{\left[0,\infty \right)}\left({𝒞}_{N}\right)$-*valued process* ${\left(\xi_{t}\right)}_{t\geq 0}$ *is a solution to the* $\left(A^{N},\xi_{0}\right)$ *martingale problem if* $f\left(\xi_{t}\right)-f\left(\xi_{0}\right)-\int_{0}^{t}\;A^{N}f\left(\xi_{s}\right)ds$ *is a martingale (with respect to the natural filtration) for all test functions* f *as defined above*.

The martingale problem for finite N has a unique solution. Next we state the limiting martingale problem, for which we do not necessarily have uniqueness. As before, the parameter α will correspond to ${lim}_{N\to \infty }\;\theta (N)/N$. Whereas for finite N, conditional on the population process $\eta_{t}^{N}$, the levels of particles are independent and uniformly distributed on [0,N], in the infinite population limit, conditional on $\eta_{t}$, the process $\xi_{t}$ is Poisson distributed on $R^{d}\times [0,\infty )$ with mean measure $\eta_{t}\times \lambda$, where λ is Lebesgue measure.

#### Definition 5.2 (Martingale Problem Characterisation, scaling limit)

*Fix* α∈[0,∞), *and define test functions* f *by* $f(\xi )=\prod_{(x,u)\in \xi }\;g(x,u)$ *with* g differentiable in u, smooth in x, satisfying 0≤g(x,u)≤1 and such that there exists a $u_{0}$ with g(x,u)=1 *for all* $u>u_{0}$. *Then, define the operator* A *on such test functions by*

(5.11) $$\begin{array}{l} Af(\xi )=f(\xi )\sum_{(x,u)\in \xi }\gamma (x,\eta )\frac{ℬ(g(\cdot ,u)r(\cdot ,\eta ))(x)-g(x,u)ℬr(x,\eta )}{g(x,u)}\\ \;\;\;\;\;\;\;\;\;\;\;\;\;\;\;\;\;\;\;\;+f(\xi )\sum_{(x,u)\in \xi }2\alpha \gamma (x,\eta )r(x,\eta )\int_{u}^{\infty }\left(g\left(x,u_{1}\right)-1\right)du_{1}\\ \;\;\;\;\;\;\;\;\;\;\;\;\;\;\;\;\;\;\;\;+f(\xi )\sum_{(x,u)\in \xi }\left(\alpha \gamma (x,\eta )r(x,\eta )u^{2}-\{\gamma (x,\eta )ℬr(x,\eta )+F(x,\eta )\}u\right)\frac{\partial_{u}g(x,u)}{g(x,u)}. \end{array}$$

*We say that a* ${𝒟}_{\left[0,\infty \right)}(𝒞)$-*valued process* ${\left(\xi_{t}\right)}_{t\geq 0}$ *is a solution to the* $\left(A,\xi_{0}\right)$ *martingale problem if it has initial distribution* $\xi_{0}$ *and* $f\left(\xi_{t}\right)-f\left(\xi_{0}\right)-\int_{0}^{t}\;Af\left(\xi_{s}\right)$ *ds is a martingale (with respect to the natural filtration) for all test functions* f *as defined above*.

The lookdown processes have been carefully constructed so that observations about the past spatial positions of individuals in the population do not give us any information about the assignment of individuals to levels. In other words, the dynamics of the lookdown process preserve the conditionally uniform (or in the limit, conditionally Poisson) structure – if started with uniform levels, levels are uniform at all future times. Moreover, if we average over levels in the expression for the generator (equation (5.10) or (5.11)) we recover the generator for the population process. Once this is verified (along with some boundedness conditions) the Markov Mapping Theorem (Theorem A.1; also see Etheridge and Kurtz [2019]) tells us that by “removing labels” from the lookdown process ξ we recover the population process η.

To make this precise, define the spatial projection maps $\kappa^{N}:ℳ\left(R^{d}\times [0,N]\right)\to ℳ\left(R^{d}\right)$ by $\kappa^{N}\left(\xi^{N}\right)(\cdot )=\xi^{N}(\cdot \times [0,N])/N$, and $\kappa :ℳ\left(R^{d}\times [0,\infty )\right)\to ℳ\left(R^{d}\right)$ by $\kappa (\xi )(\cdot )={lim}_{u_{0}\to \infty }\;\xi (\cdot \times \left.\left[0,u_{0}\right]\right)/u_{0}$. We will also need an inverse notion: for a measure $\xi^{N}$ on $R^{d}\times [0,\;N]$ and a σ-field ℱ, we say that $\xi^{N}$ *is conditionally uniform given* ℱ if $\kappa^{N}(\xi )$ is ℱ-measurable and for all compactly supported f,

(5.12) $$E\left[e^{-\langle f,\xi \rangle }|ℱ\right]=e^{-\langle H_{f}^{N},\kappa^{N}(\xi )\rangle },$$

where

$$H_{f}^{N}(x)=-N\;log\frac{1}{N}\int_{0}^{N}\;e^{-f(x,u)}du.$$

In other words, the [0,N] components of ξ are independent, uniformly distributed on [0,N], and independent of $\kappa^{N}(\xi )$. Similarly, for a measure ξ on $R^{d}\times [0,\infty )$ we say that ξ *is a conditionally Poisson random measure given* ℱ if κ(ξ) is ℱ-measurable and for all compactly supported f,

(5.13) $$E\left[e^{-\langle f,\xi \rangle }|ℱ\right]=e^{-\langle \int_{0}^{\infty }(1-e^{-f(x,u)})du,\kappa (\xi )(dx)\rangle }.$$

In other words, ξ is conditionally Poisson with Cox measure κ(ξ)×λ, where λ is Lesbegue measure.

#### Proposition 5.3

If ${\tilde{\eta }}^{N}$ is a solution of the martingale problem of Definition 2.4 with initial distribution $\eta_{0}^{N}$ then there exists a solution $\xi^{N}$ of the $\left(A^{N},\xi_{0}^{N}\right)$-martingale problem of Definition 5.1 such that $\eta^{N}=\kappa^{N}\circ \xi^{N}$ has the same distribution on $D_{{ℳ}_{F}\left(R^{d}\right)}[0,\infty )$ as ${\tilde{\eta }}^{N}$. Furthermore, for each $t,\;\xi_{t}^{N}$ is conditionally uniform given ${ℱ}_{t}^{\eta^{N}}$ in the sense of (5.12).

Similarly, if $\tilde{\eta }$ is a solution of the limiting martingale problem of Theorem 2.10 with initial distribution $\eta_{0}$ then there exists a solution ξ of the martingale problem of of Definition 5.2 such that η=κ∘ξ has the same distribution on $D_{{ℳ}_{F}\left(R^{d}\right)}[0,\infty )$ as $\tilde{\eta }$. Furthermore, $\xi_{t}$ is conditionally Poisson given ${ℱ}_{t}^{\eta }$ in the sense of (5.13).

Now we can present the main convergence theorem that is analogous to Theorem 2.10 for the population process.

#### Theorem 5.4

*Let* $\left(\xi_{t}^{N}\right)$ *satisfy* Definition 5.1 *and assume that as* N→∞,θ→∞ *in such a way that* θ/N→α. *Let* $\eta_{0}^{N}=\kappa \left(\xi_{0}^{N}\right)$ *and suppose also that* $\eta_{0}^{N}\to \eta_{0}$ *in* ${ℳ}_{F}\left(R^{d}\right)$, *and that for each* $N,\;\xi_{0}^{N}$ *is conditionally uniform given* $\eta_{0}^{N}$ *in the sense of* (5.12). *Then*, ${\left(\xi_{t}^{N}\right)}_{t\geq 0}$ *has a subsequence which converges in distribution as* N→∞ *to a measure-valued process* ${\left(\xi_{t}\right)}_{t\geq 0}$ *with* $\xi_{t}$ *conditionally Poisson given* $\eta_{t}=\kappa \left(\xi_{t}\right)$ *for each* t *in the sense of* (5.13), *that is a solution to the martingale problem of* Definition 5.2.

Both results are proved in Section 8.

### 5.2 Explicit construction of lines of descent

The main interest in using a lookdown construction for our population processes is that it allows us to retain information about the relatedness of individuals as we pass to the infinite population limit. In order to exploit this, in this section we write down stochastic equations for the locations and levels of individuals in the prelimiting lookdown model. We will then be able to pass to the scaling limit. This provides an explicit description of the solution to the limiting martingale problem of Definition 5.2 which will enable us to identify all individuals in the current population that are descendants of a given ancestor at time zero. In theory at least, this allows us to recover all the information about genealogies relating individuals sampled from the present day population. This idea draws on the notion of “tracers”, popular in statistical physics and used in population genetics by a number of authors including Hallatschek and Nelson [2008], Durrett and Fan [2016], and Biswas et al. [2021].

We will construct the process using a Ulam-Harris indexing scheme. First, we assign each individual alive at time 0 a unique label from N. Suppose an individual with label a and level u reproduces, and as a result there are two individuals, one with level u and one with a new level $u_{1}>u$. The parent individual, previously labeled a, might be assigned either level. We will track chains of descendant individuals forwards through time by following levels, rather than individuals, and will call this a *line of descent*. So, after reproduction, we give a new label to *only* the individual that is given the new level $u_{1}$, retaining the label a for the individual with the old level u. In this way, at each birth event, a unique label is assigned to the resulting individual with the higher level, and the label of an individual may change throughout its lifetime.

Concretely, then: for each label a in $ℐ={⋃}_{k\geq 1}\;N^{k}$, let $\Pi_{a}$ be an independent Poisson process on $\left[0,\infty {)}^{2}\times R^{d}\times \{0,1\right\}$. The mean measure of each $\Pi_{a}$ is a product of Lebesgue measure on $[0,\infty {)}^{2}$, the density of the standard Gaussian on $R^{d}$, and $\left(\delta_{0}+\delta_{1}\right)/2$ on {0, 1}. It will also be convenient to suppose that for each label a we have an enumeration of the points in $\Pi_{a}$, so we may refer to “the *j*^th^ point in $\Pi_{a}$“, although the precise order of this enumeration is irrelevant. If (τ,v,z,κ) is the *j*^th^ point in $\Pi_{a}$, then τ will determine a possible birth time, v will determine the level of the offspring, z will determine the spatial displacement of the offspring relative to the parent, κ will be used to determine whether parent or offspring is assigned the new level, and the new label produced will be a⊕j, i.e., the label a with j appended (so, if $a=\left(a_{1},\ldots ,a_{k}\right)$ then $a⊕j=\left(a_{1},\ldots ,a_{k},j\right)$). Each label a has a birth time $\tau_{a}$, when it is first assigned, and a (possibly infinite) death time $\sigma_{a}$, when its level first hits N. For any $\tau_{a}\leq t\leq \sigma_{a}$ we denote by $X_{a}(t)$ and $U_{a}(t)$ the spatial location and level of the individual carrying label a at time t, respectively. Furthermore, define

$$\eta_{t}^{N}=\frac{1}{N}\sum_{a:\tau_{a}\leq t<\sigma_{a}}\delta_{X_{a}(t)}\;\text{and}\;\;\xi_{t}^{N}=\sum_{a:\tau_{a}\leq t<\sigma_{a}}\delta_{\left(X_{a}(t),U_{a}(t)\right)}.$$

Now, since we have defined labels so that the level does not jump, $U_{a}$ satisfies (5.5) for $\tau_{a}\leq t\leq \sigma_{a}$, i.e.,

(5.14) $$U_{a}(t)\;=U_{a}\left(\tau_{a}\right)\;\;+\int_{\tau_{a}}^{t}(c_{\theta }\left(X_{a}(s),\eta_{s}\right)U_{a}(s{)}^{2}-b_{\theta }\left(X_{a}(s),\eta_{s}\right)U_{a}(s))ds,$$

and, of course, $\sigma_{a}=inf\left\{t\geq \tau_{a}:U_{a}(t)>N\right\}.$

Potential reproduction events occur at times τ for each point $(\tau ,v,z,\kappa )\in \Pi_{a}$ with $\tau_{a}\leq \tau <\sigma_{a}$. (We say “potential” since if the level of the resulting offspring is greater than N, the event does not happen.) If this is the *j*^th^ point in $\Pi_{a}$, the potential new label is a⊕j, the birth time is $\tau_{a⊕j}=\tau$, and the spatial displacement of the potential offspring is y(X(τ-),z), where

$$y\left(x,z\right):=\frac{1}{\theta }\vec{b}\left(x\right)+\frac{1}{\sqrt{\theta }}K\left(x\right)z,$$

and $K\left(x\right)K^{T}\left(x\right)=C\left(x\right).$

Next we must choose the new level created at the birth event. We would like an individual with level u and at spatial position x to produce offspring at y at instantaneous rate

(5.15) $$2\left(1-\frac{u}{N}\right)\theta \gamma \left(x,\eta \right)r\left(x+y,\eta \right).$$

To do this we will associate the point $(\tau ,v,z,\kappa )\in \Pi_{a}$ with level u+vℓ, where ℓ is chosen so that the rate of appearance of points in $\Pi_{a}$ with level below N, that is points with vℓ<N-u, is given by (5.15). Since the mean measure of $\Pi_{a}$ is Lebesgue measure in the t and v directions, we must take

(5.16) $$\ell (x,y,\eta )=\frac{N-u}{2(1-u/N)\theta \gamma (x,\eta )r(x+y,\eta )}=\frac{1}{2N^{-1}\theta \gamma (x,\eta )r(x+y,\eta )},$$

and, using this, the (potential) new level is

$$U_{a⊕j}(\tau )=U_{a}(\tau )+v\ell \left(X_{a}(\tau -),y\left(X_{a}(\tau -),z\right),\eta_{\tau -}\right).$$

If $U_{a⊕j}(\tau )<N$, the new individual labeled a⊕j is produced, and κ determines which label, a or a⊕j, is associated with the new location, so

$$X_{a⊕j}\left(\tau \right)=X_{a}\left(\tau -\right)+\left(1-\kappa \right)y\left(X_{a}\left(\tau -\right),z\right).$$

On the other hand if $U_{a⊕j}(\tau )\geq N$, then $X_{a}$ is unchanged and $X_{a⊕j}$ is undefined, so

(5.17) $$X_{a}(\tau )=X_{a}(\tau -)+\kappa y\left(X_{a}(\tau -),z\right)1_{U_{a⊕j}(\tau )<N}.$$

Recall that the parental individual always retains their spatial location, so that κ=0 corresponds to the parent being assigned a new level, and our line of descent switching to the offspring. Combining these observations, $X_{a}$, for $\tau_{a}\leq t<\sigma_{a}$, solves the equation

$$X_{a}\left(t\right)=X_{a}\left(\tau_{a}\right)+\int_{\left[\tau_{a},t\right)\times \left[0,\infty \right)\times R\times \left[0,1\right]}y\left(X_{a}\left(\tau -\right),z\right)\kappa 1_{U_{a}\left(\tau \right)+v\ell \left(X_{a}\left(\tau -\right),y\left(X_{a}\left(\tau -\right),z\right),\eta_{\tau -}\right)<N}d\Pi_{a}\left(\tau ,v,z,\kappa \right).$$

Although we have described the evolution of a line of descent only for a given label (i.e., for $\tau_{a}\leq t<\sigma_{a}$), we can extend the definition to times $0\leq t<\sigma_{a}$ by setting $X_{a}(t)$ equal to $X_{[a{]}_{t}}(t)$, where $[a{]}_{t}$ is the label of the ancestor of label a alive at time t, and similarly for $U_{a}(t)$. It is then straightforward, albeit tedious, to write down the time evolution of $\left(X_{a}(t),U_{a}(t)\right)$ for all time back to t=0 in terms of the driving Poisson processes.

#### Remark 5.5

Although we have a single construction that couples the processes across all N, unlike in Kurtz and Rodrigues [2011] the actual trajectories, $X_{a}(\cdot )$, do not necessarily coincide for different values of N, since they are affected by the whole population process. However, this does suggest approximating the genealogies in the infinite density limit by simulating up until a sufficiently high level that we have a good approximation to the population process.

### 5.3 Limiting processes for lines of descent

The previous section constructed the lookdown process using the same underlying Poisson processes ${\left\{\Pi_{a}\right\}}_{a\in ℐ}$ for different values of N. As a result, if the spatial projections η converge, then individual lines of descent converge pointwise as N→∞. To see this, first note that if the Poisson processes are fixed then the set of events with which a given label a∈ℐ is associated is also fixed – this is the sequence $\left(\tau_{k},v_{k},z_{k},\kappa_{k}\right)$ associated with the label a. To conclude that the lines of descent converge, first, we clearly need that the spatial projections η converge. Supposing that they do, consider how a line of descent $\left(X_{a}(t),U_{a}(t)\right)$ evolves. It throws off a new line of descent at a higher level when there is a point (τ,v,z,κ) in $\Pi_{a}$ with $\tau >\tau_{a}$ and

(5.18) $$v<2\frac{\left(N-U_{a}(\tau )\right)}{N}\theta \gamma \left(X_{a}(\tau -),\eta_{\tau -}\right)r\left(X_{a}(\tau -)+y\left(X_{a}(\tau -),z\right),\eta_{\tau -}\right).$$

Since the mean measure of the v coordinate is Lebesgue measure, θ/N→α, and $q_{\theta }(x,dy)\to \;\delta_{x}(dy)$, this corresponds in the limit to new lines of descent being thrown off according to a Poisson process with intensity

$$2\alpha \gamma \left(X_{a}(t),\eta_{t}\right)r\left(X_{a}(t),\eta_{t}\right)dt\times du.$$

Now consider the location of the line of descent: at each birth event, with probability one half the line of descent jumps to $X_{a}(t)+y$. Taking g to be a suitable test function on $R^{d}$, and rewriting (5.18), when the level is u and the state of the population is η, the generator of the spatial motion of the line of descent applied to g(x) is

$$(1-\left.\frac{u}{N}\right)\gamma (x,\eta )\theta \int_{R^{d}}\;r(x+y,\eta )(g(x+y)-g(x))q_{\theta }(x,dy)\;\;=\left(1-\frac{u}{N}\right)\gamma (x,\eta )\{\theta \int_{R^{d}}\;(r(x+y,\eta )g(x+y)-r(x,\eta )g(x))q_{\theta }(x,dy)\;\;-\theta \int_{R^{d}}\;(r(x+y,\eta )-r(x,\eta ))g(x)q_{\theta }(x,dy)\}\;\;\to \gamma \left(x,\eta \right)\left(ℬ\left(rg\right)\left(x\right)-g\left(x\right)ℬ\left(r\right)\left(x\right)\right),\;\text{as}\;N,\theta \to \infty .$$

Notice that the factors of 2 have cancelled, and that the result is independent of u. Also recall that r(x,η) depends on η only through $\rho_{r}*\eta (x)$, which is guaranteed to be smooth, so that ℬ(r) and ℬ(gr) are well-defined.

We write out the differential operator above in more detail. Recall that $ℬg(x)=\sum_{i}{\vec{b}}_{i}\partial_{i}g(x)+\sum_{ij}\;C_{ij}\partial_{ij}g(x)$, and for the moment write r(x) for r(x,η), $\vec{b}(x)=\vec{b}$, and C(x)=C so that

(5.19) $$ℬ(rg)(x)-g(x)ℬ(r)(x)=r(x)\sum_{i}{\vec{b}}_{i}\partial_{i}g(x)+2\sum_{ij}\partial_{i}r(x)C_{ij}\partial_{j}g(x)+r(x)\sum_{ij}C_{ij}\partial_{ij}g(x)\;\;\;\;\;\;\;\;\;\;\;\;\;\;\;\;\;\;\;\;\;\;\;\;\;\;\;\;\;\;\;\;\;\;\;\;\;\;\;\;\;\;\;=r(x)\left\{\left(\vec{b}+2C\nabla \log r(x))\cdot \nabla g(x)+\sum_{ij}C_{ij}\partial_{ij}g(x\right)\right\}.$$

The only thing that remains is to describe how the levels change, but this is immediate from applying limit (5.4) to equation (5.5).

We summarize the results in a proposition.

#### Proposition 5.6 (Line of descent construction)

Define J(x,η) and β(x,η) by

$$r(x,\eta )\gamma (x,\eta )C(x)=J(x,\eta )J(x,\eta {)}^{T}$$

$$\beta (x,\eta )=r(x,\eta )\gamma (x,\eta )(\vec{b}(x)+2C(x)\nabla \;log\;r(x,\eta )).$$

*Associate with each label* $a\in ℐ=\cup_{k\geq 1}N^{k}$ an independent d-dimensional Brownian motion $W_{a}$ *and an independent Poisson process* $R_{a}$ *on* $[0,\infty {)}^{2}$ *with Lebesgue mean measure, and with points ordered in some way. Given* $\eta_{0}\in {ℳ}_{F}\left(R^{d}\right)$, *let* $\left(x_{i},u_{i}\right)$ *be the points of a Poisson process on* $R^{d}\times [0,\infty )$ *with mean measure* $\eta_{0}\times \lambda$ *(the product of* $\eta_{0}$ *and Lebesgue measure). For each* i, *begin a line of descent with label* i, *location* $X_{i}(0)=x_{i}$, *level* $U_{i}(0)=u_{i}$, *and birth time* $\tau_{i}=0$.

Write $\tau_{a}$ for the birth time of the label a and $\sigma_{a}={lim}_{u_{0}\to \infty }\;inf\left\{t\geq 0:U_{a}(t)>u_{0}\right\}$ *the time the level hits* ∞. S*uppose that the spatial locations and level of each line of descent a solve, for* $\tau_{a}\leq t<\sigma_{a}$,

(5.20) $$X_{a}(t)=X_{a}\left(\tau_{a}\right)+\;\int_{\tau_{a}}^{t}\beta \left(X_{a}(s),\eta_{s}\right)ds+\int_{\tau_{a}}^{t}J\left(X_{a}(s),\eta_{s}\right)dW_{a}(s)\;U_{a}(t)=U_{a}\left(\tau_{a}\right)+\;\int_{\tau_{a}}^{t}\left(\alpha \gamma \left(X_{a}(s),\eta_{s}\right)r\left(X_{a}(s),\eta_{s}\right)U_{a}(s{)}^{2}\right.\;\;\left.-\left\{\gamma \left(X_{a}(s),\eta_{s}\right)ℬr\left(X_{a}(s),\eta_{s}\right)+F\left(X_{a}(s),\eta_{s}\right)\right\}U_{a}(s)\right)ds.$$

*Each point in each* $R_{a}$ denotes a potential birth time for a: if the *j*^th^ *point in* $R_{a}$ *is* (τ,v), *with* $\tau_{a}\leq \tau <\sigma_{a}$, *then a new line of descent with label* a⊕j *is produced, with birth time* $\tau_{a⊕j}=\tau$, *location* $X_{a⊕j}(\tau )=X_{a}(\tau )$, *and level*

$$U_{a⊕j}(\tau )=U_{a}(\tau )+\frac{v}{2\alpha \gamma \left(X_{a}(\tau ),\eta_{\tau }\right)r\left(X_{a}(\tau ),\eta_{\tau }\right)},$$

*if this is finite. For any solution to the equations above, the processes defined by*

$$\eta_{t}=\underset{u_{0}\to \infty }{lim}\;\frac{1}{u_{0}}\sum_{a:\tau_{a}\leq t<\sigma_{a};U_{a}(t)<u_{0}}\delta_{X_{a}(t)}\;and\;\xi_{t}=\sum_{a:\tau_{a}\leq t<\sigma_{a}}\delta_{\left(X_{a}(t),U_{a}(t)\right)}$$

are solutions to the martingale problems of Theorems 2.10 and 5.4, respectively.

In particular, note that if α=0, no new lines of descent are produced. More precisely, comparing with (5.16), they are produced, but “at infinity”, and their trace is seen in the spatial motion of the line of descent which results from the production of these lineages.

#### Proof [of Proposition 5.6]

The fact that a solution to the system of equations (5.20) is a solution to the martingale problem of Theorem 5.4 is an application of Itô’s theorem. Furthermore, in Proposition 5.3 we showed that the conditional Poisson property of $\xi_{0}$ is preserved (i.e., holds for $\xi_{t}$ for all), and so ${\left(\eta_{t}\right)}_{t\geq 0}$ is well-defined, and furthermore that $\eta_{t}$ is a solution to the martingale problem of Theorem 2.10. □

Proofs of the remainder of these results are in Section 8.

### Remark 5.7

The process we consider is similar to the state-dependent branching processes of Kurtz and Rodrigues [2011], so one might expect that the proofs there would carry over with little change. However, there is an important difference: Recall that the level $U_{a}(t)$ of a line of descent evolves as

(5.21) $$\dot{u}=c_{\theta }(x,\eta )u^{2}-b_{\theta }(x,\eta )u,$$

*where* $b_{\theta }(x,\eta )$ *and* $c_{\theta }(x,\eta )$ *are defined in* (5.3) *and* (5.2) respectively. Note that $c_{\theta }(x,\eta )\geq 0$, while $b_{\theta }(x,\eta )$ may take either sign. Assumptions 2.8 imply that $c_{\theta }(x,\eta )$ is bounded, while $b_{\theta }(x,\eta )$, because of F(x,η), is bounded above but not necessarily below. In Kurtz and Rodrigues [2011], $b_{\theta }$ was bounded above and $c_{\theta }$ was bounded away from *zero, so they noted that if* $U_{a}(t)\geq b_{\theta }/c_{\theta }$ for some label a, that line of descent would only move upwards from that time onwards. Furthermore, coefficients did not depend on the state of the process (i.e., on η thus allowing the processes to be jointly and simultaneously constructed for all values of N, with a pointwise embedding of ${\left(\xi_{t}^{N}\right)}_{t>0}$ *within* ${\left(\xi^{M}\right)}_{t>0}$ *for* $b_{\theta }/c_{\theta }<N<M$. *In other words, individuals with levels above* $N>b_{\theta }/c_{\theta }$ *at time* $t_{0}$ *do not affect* ${\left(\xi_{t}^{N}\right)}_{t\geq t_{0}}$, *thus allowing a comparison of the number of lines of descent below level* $u_{0}$ *to a branching process. Although we have provided a joint construction of* $\xi^{N}$ *for all* N *in* Section 5.2, *it does not have this monotonicity: for one thing*, $b_{\theta }$ *and* $c_{\theta }$ *depend on the population process* η *and so all individuals can affect all other ones (even those with lower levels). Furthermore, in the deterministic case* θ/N, *and hence c, converges to zero, and so lines of descent with arbitrarily high level may drift back downwards. Indeed, this must be the case if the population persists, since in the deterministic case there is no branching*.

## 6 Proofs of convergence for nonlocal models

In this section we present formal proofs of the first two of our three scaling limits. In Subsection 6.2 we prove Theorem 2.10, to obtain (both stochastic and deterministic) limits in which interactions between individuals in the population are nonlocal. In Subsection 6.3 we show how, in two important examples in which the nonlocal limit is respectively a deterministic solution to a non-local equation of reaction-diffusion type and a deterministic solution to a nonlocal porous medium equation with an additional logistic growth term, one can pass to a further limit to obtain a classical PDE.

### 6.1 Preliminaries

Below we will have frequent use for the quantity

(6.1) $$B_{f}^{\theta }(x,\eta )=\theta \int_{R^{d}}(f(y)r(y,\eta )-f(x)r(x,\eta ))q_{\theta }(x,dy).$$

First, we prove Lemma 2.9.

#### Proof [of Lemma 2.9]

Here, we need to prove that $\left|\gamma (x,\eta )B_{f}^{\theta }(x,\eta )\right|$ is bounded, uniformly over x and η. First suppose that Condition 1 of Lemma 2.9 is satisfied. We write

$$r(y,\eta )f(y)-r(x,\eta )f(x)=r(y,\eta )(f(y)-f(x))+(r(y,\eta )-r(x,\eta ))f(x)\;\;=r(y,\eta )\left(\sum_{i}\;(y-x{)}_{i}\partial_{x_{i}}f(x)+\sum_{ij}\;(y-x{)}_{i}(y-x{)}_{j}\partial_{x_{i}x_{j}}f\left(z_{1}\right)\right)\;\;\;+f(x)\left(\sum_{i}\;(y-x{)}_{i}\partial_{x_{i}}r(x,\eta )+\sum_{ij}\;(y-x{)}_{i}(y-x{)}_{j}\partial_{x_{i}x_{j}}r\left(z_{2},\eta \right)\right)\;\;=\left(r(x,\eta )+\sum_{j}\;(y-x{)}_{j}\partial_{x_{j}}r\left(z_{3},\eta \right)\right)\left(\sum_{i}\;(y-x{)}_{i}\partial_{x_{i}}f(x)\right)\;\;\;+r(y,\eta )\sum_{ij}\;(y-x{)}_{i}(y-x{)}_{j}\partial_{x_{i}x_{j}}f\left(z_{1}\right)\;\;\;+f(x)\left(\sum_{i}\;(y-x{)}_{i}\partial_{x_{i}}r(x,\eta )+\sum_{ij}\;(y-x{)}_{i}(y-x{)}_{j}\partial_{x_{i}x_{j}}r\left(z_{2},\eta \right)\right),$$

for some $z_{i}=\kappa_{i}x+\left(1-\kappa_{i}\right)y$. Integrating this against q(x,dy), we get that

$$|\theta \int \;(r(y,\eta )f(y)-r(x,\eta )f(x))q_{\theta }(x,dy)|\;\;\leq \;|\sum_{i}\;\left(r(x,\eta )\partial_{x_{i}}f(x)+f(x)\partial_{x_{i}}r(x,\eta )\right)\theta \int (y-x{)}_{i}q_{\theta }(x,dy)|\;\;+\left|f(x)\theta \int \sum_{ij}\;\partial_{x_{i}x_{j}}r\left(z_{2},\eta \right)(y-x{)}_{i}(y-x{)}_{j}q_{\theta }(x,dy)\right|\;\;+\left|\theta \int \sum_{ij}\;(y-x{)}_{i}(y-x{)}_{j}\left(\partial_{x_{i}}f(x)\partial_{x_{j}}r\left(z_{3},\eta \right)+r(y,\eta )\partial_{x_{i}x_{j}}f\left(z_{1}\right)\right)q_{\theta }(x,dy)\right|$$

Since $q_{\theta }(x,dy)$ is the density of a Gaussian with mean $\vec{b}(x)/\theta$ and covariance C(x)/θ, and both $\vec{b}(x)$ and C(x) are uniformly bounded, so that $\theta \int (y-x{)}_{i}q_{\theta }(x,dy)$ is bounded as well. Furthermore, a change of variables that diagonalizes C(x) shows for any $g:R^{d}\to R^{d+d}$, that if $C_{g}={sup}_{y}\;{sup}_{∥z∥=1}\;\sum_{ij}\;g(y{)}_{ij}z_{i}z_{j}$ and $\lambda_{*}={sup}_{y}\;{sup}_{∥z∥=1}\;\sum_{ij}\;C(y{)}_{ij}z_{i}z_{j}$ then

$$\theta \int \sum_{ij}g(y{)}_{ij}(y-x{)}_{i}(y-x{)}_{j}q_{\theta }(x,dy)\leq C_{g}\lambda_{*}.$$

Condition 1 gives uniform bounds on the derivatives of $r(x,\eta )=r\left(x,\rho_{r}*\eta (x)\right)$ in this expression and so, provided f also has uniformly bounded first and second derivatives, we have a bound of the form

$$\left|B_{f}^{\theta }\right|\leq K_{1}+K_{2}|f(x)|,$$

for suitable constants $K_{1},\;K_{2}$ that depend only on the derivatives of f.

Now suppose instead that Condition 2 of Lemma 2.9 is satisfied. First note that

(6.2) $$\left|B_{f}^{\theta }\right|=\left|\theta \int_{R^{n}}\left\{f(y)r\left(y,\rho_{r}*\eta (y)\right)-f(x)r\left(x,\rho_{r}*\eta (x)\right)\right\}q_{\theta }(x,dy)\right|\;\;\leq |\theta \;\int_{R^{n}}\left\{f(y)r\left(y,\rho_{r}*\eta (y)\right)-f(x)r\left(x,\rho_{r}*\eta (y)\right)\right\}q_{\theta }(x,dy)|\;\;+\left|\theta \int_{R^{n}}\left\{f(x)r\left(x,\rho_{r}*\eta (y)\right)-f(x)r\left(x,\rho_{r}*\eta (x)\right)\right\}q_{\theta }(x,dy)\right|.$$

(Note the extra term introduced here, $r\left(x,\rho_{r}*\eta (y)\right.$), has the two arguments to r “at different locations”, contrary to the usual pattern.)

Writing $K_{3}={sup}_{x,m}\;{max}_{i}\;\left|\partial_{x_{i}}f(x)r(x,m)\right|$ and $K_{4}={sup}_{x,m}\;{max}_{i,j}\;\left|\partial_{x_{i}x_{j}}f(x)r(x,m)\right|$, the first term is bounded exactly as above. For the second,

$$r\left(x,\rho_{r}*\eta (y)\right)-r\left(x,\rho_{r}*\eta (x)\right)\;\;=\left(\rho_{r}*\eta (y)-\rho_{r}*\eta (x)\right)r^{\prime }\left(x,\rho_{r}*\eta (x)\right)+\frac{1}{2}{\left(\rho_{r}*\eta (y)-\rho_{r}*\eta (x)\right)}^{2}r^{\prime \prime }(x,\overset{‾}{m}),$$

where $\overset{‾}{m}=\kappa^{\prime }\rho_{r}*\eta (x)+\left(1-\kappa^{\prime }\right)\rho_{r}*\eta (y)$ for some $0\leq \kappa^{\prime }\leq 1$, and we have used $r^{\prime }$ and $r^{\prime \prime }$ to denote the first and second derivatives of r(x,m) with respect to the second argument. So, writing $K_{5}={∥r^{\prime }∥}_{\infty }$ and $K_{6}={∥r^{\prime \prime }∥}_{\infty }$, the second term in (6.2) is bounded by f(x) multiplied by

$$K_{5}|\theta \int_{R^{d}}\;\left(\rho_{r}*\eta (y)-\rho_{r}*\eta (x)\right)q_{\theta }(x,dy)|+K_{6}\left|\theta \int_{R^{d}}\;{\left(\rho_{r}*\eta (y)-\rho_{r}*\eta (x)\right)}^{2}q_{\theta }(x,dy)\right|.$$

Under Condition 2 of Assumptions 2.8, this is bounded by a constant times $\rho_{\gamma }*\eta (x)+(\rho_{\gamma }*\eta (x){)}^{2}$ and ${sup}_{x}\;m^{2}\gamma (x,m)$ is bounded, so therefore $\left|\gamma (x,\eta )B_{f}^{\theta }(x,\eta )\right|\leq K_{7}+K_{8}f(x)$, where $K_{7}$ comes from $K_{3},\;K_{4}$, and the supremum of γ, while $K_{8}$ comes from $K_{5},\;K_{6}$, and the supremum of $m^{2}\gamma (x,m)$. □

### 6.2 Proof of Theorem 2.10: convergence for the nonlocal process

In this section we prove Theorem 2.10. This would be implied by convergence of the lookdown process (see Kurtz and Rodrigues [2011] and Etheridge and Kurtz [2019]); however in our setting, because the parameters in the lookdown process depend on the empirical distribution, we actually use tightness of the sequence of population processes in the proofs of tightness for the corresponding lookdown processes.

#### Proof [Proof of Theorem 2.10.]

The proof follows a familiar pattern. First we extend $R^{d}$ to its one-point compactification ${\bar{R}}^{d}$ and establish, in Lemma 6.2, compact containment of the sequence of scaled population processes in ${ℳ}_{F}({\bar{R}}^{d})$ (for which, since we have compactified $R^{d}$, it suffices to consider the sequence of total masses); armed with this, tightness of the population processes in ${𝒟}_{\left[0,\infty \right)}({ℳ}_{F}){\bar{R}}^{d}))$ follows from tightness of the real-valued processes ${\left(H\left(\eta_{t}\right)\right)}_{t\geq 0}$ for a sufficiently large class of test functions H, which we establish through an application of the Aldous-Rebolledo criterion in Lemma 6.3. These ingredients are gathered together in Proposition 6.4 to deduce tightness of the scaled population processes in the larger space ${𝒟}_{\left[0,\infty \right)}({ℳ}_{F}({\bar{R}}^{d}))$.

We then characterise limit points as solutions to a martingale problem in Lemma 6.6; finally in Lemma 6.7 we check that in the process of passing to the limit, no mass ‘escaped to infinity’, so that in fact the limit points take values in ${𝒟}_{\left[0,\infty \right)}\left({ℳ}_{F}\left(R^{d}\right)\right)$. □

As advertised, we work with the one-point compactification of $R^{d}$ and consider ${\left(\eta_{t}^{N}\right)}_{t\geq 0}$ as a sequence of ${ℳ}_{F}({\bar{R}}^{d})$-valued processes. Since, for each K>0, {η:⟨1,η⟩≤K} is a compact set in ${ℳ}_{F}({\bar{R}}^{d})$, we shall focus on controlling ${\left(\left\langle 1,\eta_{t}^{N}\right\rangle \right)}_{t\geq 0}$. The key is that Assumptions 2.8 are precisely chosen to guarantee boundedness of the net per-capita reproduction rate.

#### Lemma 6.1

Under Assumptions 2.8, for all $f\in C_{b}^{2}\left(R^{d}\right)$ with uniformly bounded first and second derivatives, and all T>0, there exists a C=C(f,T)<∞, independent of N, such that

(6.3) $$E\left[\left\langle f,\eta_{t}^{N}\right\rangle \right]\leq CE\left[\left\langle 1,\eta_{0}^{N}\right\rangle \right]$$

*for all* N≥1.

#### Proof

Consider the semimartingale decomposition from equation (2.3):

(6.4) $$\left\langle f,\eta_{t}^{N}\right\rangle =\left\langle f,\eta_{0}^{N}\right\rangle +\int_{0}^{t}\;\int_{R^{d}}\left\{\gamma \left(x,\eta_{s}^{N}\right)B_{f}^{\theta }\left(x,\eta_{s}^{N}\right)+f\left(x\right)F\left(x,\eta_{s}^{N}\right)\right\}\eta_{s}^{N}\left(dx\right)ds+M_{t}^{N}\left(f\right),$$

where $M_{t}^{N}(f)$ is a martingale and $B_{f}^{\theta }$ is defined in (6.1). First note that Condition 6 of Assumptions 2.8 stipulates that $\left|\gamma B_{f}^{\theta }\right|$ is uniformly bounded by a contant times 1+f, and so recalling that F is bounded above, we conclude that under Assumptions 2.8 $\gamma (x,\eta )B_{f}^{\theta }(x,\eta )+f(x)F(x,\eta )\leq C_{f}(1+f(x))$ for some $C_{f}.$

Now, taking expectations in (6.4),

(6.5) $$E\left[\left\langle f,\eta_{t}^{N}\right\rangle \right]\leq E\left[\left\langle f,\eta_{0}^{N}\right\rangle \right]+C_{f}\int_{0}^{t}\;E\left[\left\langle 1+f,\eta_{s}^{N}\right\rangle \right]dt.$$

The bound (6.3) then follows by first applying Gronwall’s inequality in the case f=1, which yields

$$E\left[\left\langle 1,\eta_{t}^{N}\right\rangle \right]\leq e^{Ct}E\left[\left\langle 1,\eta_{0}^{N}\right\rangle \right],$$

with C independent of N, and then substituting the resulting bound on $E\left[\left\langle 1,\eta_{s}^{N}\right\rangle \right]$ into the expression above. □

With a bound on per-capita net growth rate in hand, bounds on the expectation of the supremum of the total population size over a finite time interval also follow easily.

#### Lemma 6.2 (Compact containment for the population process)

Under the assumptions of Theorem 2.10, for each T>0, there exists some constant $C_{T}$, independent of N, such that

(6.6) $$E\left[\underset{0\leq t\leq T}{sup}\;\left\langle 1,\eta_{t}^{N}\right\rangle \right]\leq C_{T}E\left[\left\langle 1,\eta_{0}\right\rangle \right].$$

In particular, for any δ>0, there exists $K_{\delta }>0$ such that

(6.7) $$\underset{N\to \infty }{lim\;sup}\;P\left\{\underset{s\in [0,T]}{sup}\;\left\langle 1,\eta_{s}^{N}\right\rangle >K_{\delta }\right\}\leq \frac{C_{T}}{K_{\delta }}<\delta .$$

#### Proof

First note that by Lemma 6.1, $E\left[\left\langle 1,\eta_{t}^{N}\right\rangle \right]\leq E\left[\left\langle 1,\eta_{0}^{N}\right\rangle \right]e^{Ct}$ for some C (independent of N). Now, let $M_{t}^{N*}(f)={sup}_{0\leq s\leq t}\;M_{t}^{N}(f)$, and as before let ${\left\langle M^{N}(f)\right\rangle }_{t}$ be the angle bracket process of $M_{t}^{N}(f)$. The Burkholder-Davis-Gundy inequality says that there is a K for which $E[M_{t}^{N*}(1)]\leq KE[\sqrt{{\left[M^{N}(1)\right]}_{t}}]$, where ${\left[M^{N}(1)\right]}_{t}$ is the quadratic variation of $M^{N}(1)$. Furthermore, as discussed by Hernández-Hernández and Jacka [2022], the expectation of the quadratic variation of a local martingale is bounded by a (universal) constant multiple of the expectation of its angle bracket process [Barlow et al., 1986, Item (4.b’), Table 4.1, p. 162]. Now, since $\sqrt{x}\leq 1+x$, in the notation of Lemma 6.1, there is a $C^{\prime }$ such that

$$E[M_{t}^{N*}(1)]\;\leq C^{\prime }\left(1+E\left[{\left\langle M^{N}(1)\right\rangle }_{t}\right]\right)\;\;=C^{\prime }\left(1+\frac{\theta }{N}E\left[\int_{0}^{t}\left\langle \left\{\gamma \left(x,\eta_{s}^{N}\right)\int_{R^{d}}\;r\left(y,\eta_{s}^{N}\right)q_{\theta }(x,dy)+\mu_{\theta }\left(x,\eta_{s}^{N}\right)\},\eta_{s}^{N}(dx\right)\right\rangle ds\right]\right)\;\;=C^{\prime }\left(1+E\left[\int_{0}^{t}\left\langle \left\{\frac{2\theta }{N}\gamma \left(x,\eta_{s}^{N}\right)r\left(x,\eta_{s}^{N}\right)\right.\right.\right.\right.\;\;\left.\left.\left.\left.+\frac{\gamma \left(x,\eta_{s}^{N}\right)}{N}B_{1}^{\theta }\left(x,\eta_{s}^{N}\right)-\frac{1}{N}F\left(x,\eta_{s}^{N}\right)\right\},\eta_{s}^{N}(dx)\right\rangle ds\right]\right).$$

We have not assumed that F is bounded below, but to see that the term involving -F does not cause us problems, we rearrange equation (6.4) with f=1 to see that

(6.8) $$E\left[\int_{0}^{t}\left\langle -F\left(x,\eta_{s}^{N}\right),\eta_{s}^{N}(dx)\right\rangle ds\right]=E\left[\left\langle 1,\eta_{0}^{N}\right\rangle \right]-E\left[\left\langle 1,\eta_{t}^{N}\right\rangle \right]\;\;+E\left[\int_{0}^{t}\left\langle \gamma \left(x,\eta_{s}^{N}\right)B_{1}^{\theta }\left(x,\eta_{s}^{N}\right),\eta_{s}^{N}(dx)\right\rangle ds\right],$$

which is bounded since γ(x,η) and $B_{1}^{\theta }(x,\eta )$ are both bounded and $\left\langle 1,\eta_{t}\right\rangle \geq 0$. Since θ/N→α<∞, combining constants, we obtain that for some $C^{\prime \prime }$,

$$E[M_{t}^{N*}(1)]\leq C^{\prime }+C^{\prime \prime }E\left[\left\langle 1,\eta_{0}^{N}\right\rangle \right]e^{tC}.$$

Taking suprema and expectations on both sides of equation (6.4), then again using the fact that $\gamma (x,\eta )B_{1}^{\theta }(x,\eta )+F(x,\eta )\leq C$,

$$E\left[\underset{0\leq s\leq T}{sup}\;\left\langle 1,\eta_{s}^{N}\right\rangle \right]\;\leq E\left[\left\langle 1,\eta_{0}^{N}\right\rangle \right]+E\left[\underset{0\leq t\leq T}{sup}\;\int_{0}^{t}\left\langle \left\{\gamma \left(x,\eta_{s}^{N}\right)B_{1}^{\theta }\left(x,\eta_{s}^{N}\right)+F\left(x,\eta_{s}^{N}\right)\right\},\eta_{s}^{N}(dx)\right\rangle ds\right]\;\;+E[M_{t}^{N*}(1)]\;\;\leq E\left[\left\langle 1,\eta_{0}^{N}\right\rangle \right]+CE\left[\int_{0}^{T}\underset{0\leq s\leq t}{sup}\;\left\langle 1,\eta_{s}^{N}\right\rangle dt\right]+C^{\prime }+C^{\prime \prime }E\left[\left\langle 1,\eta_{0}^{N}\right\rangle \right]e^{tC}.$$

Once again applying Gronwall’s inequality,

$$E\left[\underset{0\leq s\leq T}{sup}\;\left\langle 1,\eta_{s}^{N}\right\rangle \right]\leq C^{\prime \prime \prime }\left(1+E\left[\left\langle 1,\eta_{0}^{N}\right\rangle \right]\right)e^{2TC}.$$

For any T, the quantity on the right is bounded above by a constant C(T) independent of N. As a result, for any K>0,

$$\underset{N\to \infty }{lim\;sup}\;P\left[\underset{0\leq s\leq T}{sup}\;\left\langle 1,\eta_{s}^{N}\right\rangle \geq K\right]\leq \frac{C(T)}{K}.$$

□

Our next task is to show tightness of ${\left(\left\langle f,\eta_{t}^{N}\right\rangle \right)}_{t\geq 0}$ for $f\in C_{b}^{\infty }({\bar{R}}^{d})$.

#### Lemma 6.3 (Tightness of ${\left(\left\langle f,\eta_{t}^{N}\right\rangle \right)}_{t>0})$

*For each* $f\in C_{b}^{\infty }({\bar{R}}^{d})$, *the collection of processes* ${\left(\left\langle f,\eta_{t}^{N}\right\rangle \right)}_{t\geq 0}$ *for* N=1,2,… *is tight as a sequence of càdlàg, real-valued processes.*

#### Proof

The Aldous-Rebolledo criterion (Theorem B.2) applied to the semimartingale representation of $\left\langle f,\eta_{t}^{N}\right\rangle$ of equation (6.4), tells us that it suffices to show that for each T>0, (a) for each fixed 0≤t≤T, the sequence ${\left\{\left\langle f,\eta_{t}^{N}\right\rangle \right\}}_{N\geq 1}$ is tight, and (b) for any sequence of stopping times $\tau_{N}$ bounded by T, and for each ν>0, there exist δ>0 and $N_{0}>0$ such that

(6.9) $$\;\underset{N>N_{0}}{sup}\;\underset{t\in [0,\delta ]}{sup}\;P\left\{\left|\int_{\tau }^{\tau +t}\int_{R^{d}}\;\left\{\gamma \left(x,\eta_{s}^{N}\right)B_{f}^{\theta }\left(x,\eta_{s}^{N}\right)+f(x)F\left(x,\eta_{s}^{N}\right)\right\}\eta_{s}^{N}(dx)ds\right|>\nu \right\}<\nu ,$$

(6.10) $$\text{and}\underset{N>N_{0}}{sup}\;\underset{t\in [0,\delta ]}{sup}\;P\left\{\left|{\left[M^{N}(f)\right]}_{\tau +t}-{\left[M^{N}(f)\right]}_{\tau }\right|>\nu \right\}<\nu .$$

Tightness of $\left\langle f,\eta_{t}^{N}\right\rangle$ for fixed t follows from Lemma 6.1 and Markov’s inequality, so we focus on the remaining conditions.

The proof of Lemma 6.1 provides a uniform bound on $\gamma B_{f}^{\theta }$, but we only know that F is bounded above. However, by assumption, for each fixed value of $m,\;{sup}_{k\leq m}\;|F(x,k)|$ is uniformly bounded as a function of x. Noting that $\rho_{F}*\eta \leq \langle 1,\eta \rangle {∥\rho_{F}∥}_{\infty }$, we can use Lemma 6.2 to choose $N_{0}$ and K such that if $N>N_{0}$, then

$$P\left\{\underset{0\leq s\leq T}{sup}\;\left\langle 1,\eta_{s}^{N}\right\rangle \geq K\right\}<\nu /2,$$

we now choose $\delta_{1}$ so that

$$\delta_{1}∥f{∥}_{\infty }sup\{\underset{x}{sup}\;\left|F\left(x,k\right)\right|:k\leq K{∥\rho_{F}∥}_{\infty }\}<\nu /4,\;\underset{x,\eta }{sup}\;\gamma (x,\eta )B_{f}^{\theta }(\eta )\delta_{1}<\nu /4,$$

so that (6.9) is satisfied with $\delta =\delta_{1}$.

Similarly,

$$\left|{\left[M^{N}(f)\right]}_{\tau +t}-{\left[M^{N}(f)\right]}_{\tau }\right|\;\;=\left|\int_{\tau }^{\tau +t}\frac{\theta }{N}\int_{R^{d}}\;\left\{\gamma \left(x,\eta_{s}^{N}\right)\int_{R^{d}}\;f^{2}(y)r\left(y,\eta_{s}^{N}\right)q_{\theta }(x,dy)+\mu_{\theta }\left(x,\eta_{s}^{N}\right)f^{2}(x)\right\}\eta_{s}^{N}(dx)ds\right|\;\;=\left|\int_{\tau }^{\tau +t}\frac{\theta }{N}\int_{R^{d}}\;\left\{\gamma \left(x,\eta_{s}^{N}\right)\left(2f^{2}(x)r\left(x,\eta_{s}^{N}\right)+B_{f^{2}}^{\theta }\left(x,\eta_{s}^{N}\right)\right)-f^{2}(x)\frac{F\left(x,\eta_{s}^{N}\right)}{\theta }\right\}\eta_{s}^{N}(dx)ds\right|,$$

and so using the fact that θ/N→α<∞, an argument entirely analogous to that for (6.9) yields a $\delta_{2}$ for which (6.10) is satsified. Taking $\delta =min\left\{\delta_{1},\delta_{2}\right\}$, the result follows. □

We collect the implications of the last two lemmas into a proposition.

#### Proposition 6.4 (Tightness of ${\left(\eta_{t}^{N}\right)}_{t\geq 0}$)

The collection of measure-valued processes $\left\{{\left(\eta_{t}^{N}\right)}_{t\geq 0}:\}N\geq 1\right\}$ is tight in ${𝒟}_{\left[0,\infty \right)}({ℳ}_{F}({\bar{R}}^{d}))$.

#### Proof

Theorem 3.9.1 in Ethier and Kurtz [1986] says that if the collection of E-valued processes satisfies a compact containment condition (for any ϵ>0 and T>0, there is a compact set such that the processes stay within that set up to time T with probability at least 1-ϵ), then the collection is relatively compact (which is equivalent to tightness since we are working on a Polish space) if and only if $\left\{{\left(f\left(\eta_{t}^{N}\right)\right)}_{t\geq 0}:N\geq 1\right\}$ is relatively compact for all f in a dense subset of $C_{b}(E)$ under the topology of uniform convergence in compact sets.

Since {ν:⟨1,ν⟩≤K} is compact in ${ℳ}_{F}({\bar{R}}^{d})$, Lemma 6.2 gives compact containment. Lemma 6.3 shows that the real-valued processes $\left\langle f,\eta_{t}^{N}\right\rangle$ are relatively compact for all $f\in {𝒞}_{b}^{\infty }({\bar{R}}^{d})$. Since by the Stone-Weierstrass theorem, the algebra of finite sums and products of terms of this form is dense in the space of bounded continuous functions on ${ℳ}_{F}({\bar{R}}^{d})$, and tightness of $\left\langle f,\eta_{t}^{N}\right\rangle$ extends to sums and products of this form by Lemma B.3, we have relative compactness in ${𝒟}_{\left[0,\infty \right)}({ℳ}_{F}({\bar{R}}^{d}))$. □

We wish to characterise the limit points of ${\left\{{\left(\eta_{t}^{N}\right)}_{t>0}\right\}}_{N\geq 1}$ as solutions to a martingale problem with generator ${𝒫}^{\infty }$ which we now identify. Most of the work was done in Section 4. First, we record an equivalent formulation of the martingale problems, which were essentially laid out in Subsection 4.1.

#### Lemma 6.5

*For* $G\in {𝒞}^{\infty }(R)$ *with* ${∥G^{\prime \prime \prime }∥}_{\infty }<\infty$, *and* $f\in {𝒞}_{b}^{\infty }({\bar{R}}^{d})$, *define the function* $G_{f}$ *by* $G_{f}(\eta ):=G(\langle f,\eta \rangle )$. *Let* ${𝒫}^{N}$ *be the generator given by*

(6.11) $${𝒫}^{N}G_{f}(\eta ):=\theta N\langle \gamma (x,\eta )\int (G(\langle f,\eta \rangle +f(z)/N)-G(\langle f,\eta \rangle ))r(z,\eta )q_{\theta }(x,dz)\;\;+(G(\langle f,\eta \rangle -f(x)/N)-G(\langle f,\eta \rangle ))\mu_{\theta }(x,\eta ),\eta (dx)\rangle .$$

*The process* ${\left(\eta_{t}^{N}\right)}_{t\geq 0}$ *of* Definition 2.4 *is the unique solution to the* $\left({𝒫}^{N},\eta_{0}\right)$-*martingale problem, i.e., if*

$$M_{t}:=G_{f}\left(\eta_{t}^{N}\right)-G_{f}\left(\eta_{0}^{N}\right)-\int_{0}^{t}\;{𝒫}^{N}G_{f}\left(\eta_{s}^{N}\right)ds$$

is a martingale (with respect to the natural σ-field).

Furthermore, let ${𝒫}^{\infty }$ be the generator given by

(6.12) $${𝒫}^{\infty }G_{f}(\eta ):=G^{\prime }(\langle f,\eta \rangle )\langle \gamma (x,\eta )ℬ(f(\cdot )r(\cdot ,\eta ))(x)+f(x)F(x,\eta ),\eta (dx)\rangle \;\;+\alpha G^{\prime \prime }(\langle f,\eta \rangle )\left\langle \gamma (x,\eta )r(x,\eta )f^{2}(x),\eta (dx)\right\rangle .$$

*A process* ${\left(\eta_{t}^{\infty }\right)}_{t\geq 0}$ *satisifes the martingale characterization of equations (2.7) and (2.8) if and only if it is a solution to the* $\left({𝒫}^{\infty },\eta_{0}^{\infty }\right)$-*martingale problem, i.e., if for all such test functions*

$$M_{t}:=G_{f}\left(\eta_{t}^{\infty }\right)-G_{f}\left(\eta_{0}^{\infty }\right)-\int_{0}^{t}\;{𝒫}^{\infty }G_{f}\left(\eta_{s}^{\infty }\right)ds$$

is a martingale (with respect to the natural σ-field).

#### Lemma 6.6 (Characterisation of limit points)

*Suppose that* ${\left(\eta_{0}^{N}\right)}_{N\geq 1}$ *converges weakly to* $\eta_{0}$ *as* N→∞. *Then any limit point of* ${\left\{{\left(\eta_{t}^{N}\right)}_{t\geq 0}\right\}}_{N\geq 1}$ *in* ${𝒟}_{\left[0,\infty \right)}({ℳ}_{F}({\bar{R}}^{d}))$ *is a solution to the martingale problem for* $\left({𝒫}^{\infty },\eta_{0}\right)$.

#### Proof

We use Theorem 4.8.2 in Ethier and Kurtz [1986]. First observe that the set of functions $\left\{G_{f}(\eta ):=G(\langle f,\eta \rangle ),G\in {𝒞}^{\infty }(R),{∥G^{\prime \prime \prime }∥}_{\infty }<\infty ,f\in {𝒞}_{b}^{\infty }({\bar{R}}^{d})\right\}$ is separating on ${ℳ}_{F}({\bar{R}}^{d})$. Therefore, it suffices to show that for any t>0 and τ>0 that

(6.13) $$\underset{N\to \infty }{\lim}E\left[\left(G_{f}\left(\eta_{t+\tau }^{N}\right)-G_{f}\left(\eta_{t}^{N}\right)-\int_{t}^{t+\tau }{𝒫}^{\infty }G_{f}\left(\eta_{s}^{N}\right)ds\right)\prod_{i=1}^{k}h_{i}\left(\eta_{t_{i}}^{N}\right)\right]=0$$

for all $k\geq 0,\;0\leq t_{1}<t_{2}<\ldots ,t_{k}\leq t<t+\tau$, and bounded continuous functions $h_{1},\ldots ,h_{k}$ on ${ℳ}_{F}({\bar{R}}^{d})$. Since ${\left(\eta_{t}^{N}\right)}_{t\geq 0}$ is Markov, the tower property gives that, for each N,

(6.14) $$E\left[\left(G_{f}\left(\eta_{t+\tau }^{N}\right)-G_{f}\left(\eta_{t}^{N}\right)-\int_{t}^{t+\tau }{𝒫}^{N}G_{f}\left(\eta_{s}^{N}\right)ds\right)\prod_{i=1}^{k}h_{i}\left(\eta_{t_{i}}^{N}\right)\right]=0.$$

Therefore, it suffices to show that

(6.15) $$\underset{N\to \infty }{\lim}E\left[\int_{t}^{t+\tau }\left|P^{N}G_{f}\left(\eta_{s}^{N}\right)-P^{\infty }G_{f}\left(\eta_{s}^{N}\right)\right|ds\prod_{i=1}^{k}h_{i}\left(\eta_{t_{i}}^{N}\right)\right]=0,$$

and, again using the tower property, since the functions $h_{i}$ are bounded, this will follow if

(6.16) $$\underset{N\to \infty }{lim}\;E\left[\int_{t}^{t+\tau }\left|{𝒫}^{N}G_{f}\left(\eta_{s}^{N}\right)-{𝒫}^{\infty }G_{f}\left(\eta_{s}^{N}\right)\right|ds|{ℱ}_{t}\right]=0$$

(where ${\left\{{ℱ}_{t}\right\}}_{t\geq 0}$ is the natural σ-field).

We rewrite ${𝒫}^{N}G_{f}\left(\eta_{s}^{N}\right)$ using a Taylor series expansion up to third order for G(⟨f,η⟩±f(y)/N) around G(⟨f,η⟩). As in Section 4 (except that now we are more explicit about the error term), we find

(6.17) $${𝒫}^{N}G_{f}(\eta ):=G^{\prime }(\langle f,\eta \rangle )\int_{R^{d}}\;\theta \langle \gamma (x,\eta )\int_{R^{d}}\;f(y)r(y,\eta )q_{\theta }(x,dy),-f(x)\mu_{\theta }(x,\eta ),\eta (dx)\rangle \;\;+\frac{1}{2}\frac{\theta }{N}G^{\prime \prime }(\langle f,\eta \rangle )\int_{R^{d}}\;\langle \gamma (x,\eta )\int_{R^{d}}\;f^{2}(y)r(y,\eta )q_{\theta }(x,dy)+f^{2}(x)\mu_{\theta }(x,\eta ),\eta (dx)\rangle \;\;+\frac{1}{6}\frac{\theta }{N^{2}}\langle G^{\prime \prime \prime }(w)\gamma (x,\eta )\int_{R^{d}}\;f^{3}(y)r(y,\eta )q_{\theta }(x,dy)-G^{\prime \prime \prime }(v)f^{3}(x)\mu_{\theta }(x,\eta ),\eta (dx)\rangle$$

for some $w,\;v\in \left[\langle f,\eta \rangle -∥f{∥}_{\infty }/N,\langle f,\eta \rangle +∥f{∥}_{\infty }/N\right]$.

Combining with equation (4.5), and the fact that $\mu_{\theta }(x,\eta )\to r(x,\eta )\gamma (x,\eta )$ as θ→∞, we have pointwise convergence:

(6.18) $$\underset{N\to \infty }{lim}\;\left|{𝒫}^{N}G(\langle f,\eta \rangle )-{𝒫}^{\infty }G(\langle f,\eta \rangle )\right|=0.$$

To conclude convergence of the expectation, we would like to apply the Dominated Convergence Theorem in (6.15). Recall that f and G and their derivatives are bounded, and so γ(x,η) is bounded independent of θ. Since $\theta /N^{2}\to 0$, rearranging as in (4.3) and using the convergence of (4.4), we deduce that we can dominate $\left|{𝒫}^{N}G_{f}\left(\eta_{s}^{N}\right)-{𝒫}^{\infty }G_{f}\left(\eta_{s}^{N}\right)\right|$ by a constant multiple of $\left\langle 1+|F(x)|,\eta_{s}(dx)\right\rangle$. Since F is bounded above, there is a constant K such that |F|≤K-F so that, exactly as in equation (6.8), we can check that

$$E\left[\int_{t}^{t+\tau }\left\langle \left|F\left(x,\eta_{s}^{N}\right)\right|,\eta_{s}^{N}(dx)\right\rangle ds|{ℱ}_{t}\right]<\infty ,$$

which concludes our proof. □

The last step in the proof of Theorem 2.10 is to check that any limit point ${\left(\eta_{t}\right)}_{t\geq 0}$ of ${\left\{{\left(\eta_{t}^{N}\right)}_{t\geq 0}\right\}}_{N\geq 1}$ actually takes its values in ${ℳ}_{F}\left(R^{d}\right)$, that is, “no mass has escaped to infinity”.

#### Lemma 6.7

*Under the assumptions of* Theorem 2.10, *let* ${\left(\eta_{t}\right)}_{t\geq 0}$ *be a limit point of* ${\left\{{\left(\eta_{t}^{N}\right)}_{t\geq 0}\right\}}_{N\geq 1}$. *For any* δ>0,

$$P\left[\eta_{t}(\{∥x∥>R\})>\delta \right]\to 0\;as\;R\to \infty .$$

#### Proof [Sketch]

Take $f_{0}(x)$ as in the statement of Theorem 2.10, i.e., $f_{0}$ is nonnegative, grows to infinity as x→∞, has uniformly bounded first and second derivatives, and has $\left\langle f_{0},\eta_{0}^{N}\right\rangle$ uniformly bounded in N. We take a sequence of test functions $f_{n}$ that increase to the function $f_{0}$ and having uniformly bounded first and second derivatives, so that there is a (single) C from Condition 6 of Assumptions 2.8 such that $\gamma (x,\eta )B_{f_{n}}^{\theta }(x,\eta )\leq C\left(1+f_{n}(x)\right)$ for all x,η, and $f_{n}$. Then, just as we arrived at equation (6.5),

$$E\left[\left\langle f_{n}(x),\eta_{t}^{N}(dx)\right\rangle \right]\leq E\left[\left\langle f_{n}(x),\eta_{0}^{N}(dx)\right\rangle \right]+C\int_{0}^{t}\;E\left[\left\langle f_{n}(x),\eta_{s}^{N}(dx)\right\rangle \right]ds,$$

with the same constant for all n and all N. Gronwall’s inequality then implies that $E\left[\left\langle f_{n},\eta_{t}^{N}\right\rangle \right]\leq C^{\prime }$ for some $C^{\prime }$ independent of n,N, and t∈[0,T]. By first taking N→∞ and then n→∞, we find that $E\left[\left\langle f_{0},\eta_{t}(dx)\right\rangle \right]\leq C^{\prime }$ for t∈[0,T], and since $f_{0}\to \infty$ as |x|→∞, an application of Markov’s inequality tells us that for any δ>0,

$$\mathbb{P}\left\{\eta_{t}(\{x:\|x\|>R\})>\delta \right\}\leq \frac{E\left[\langle f_{0},\eta_{t}\rangle \right]}{\inf_{\left\{x:\|x\|\geq R\right\}}f_{0}(x)}\to 0\;\;\;\;\;\text{as}\;R\to \infty .$$

□

### 6.3 Convergence of some nonlocal equations to classical PDEs

It is natural to conjecture that when the limit of the rescaled population process that we obtained in the previous section solves a nonlocal PDE, if we further scale the kernels $\rho_{r},\;\rho_{\gamma }$, and $\rho_{F}$ by setting $\rho^{\epsilon }(\cdot )=\rho (\cdot /\epsilon )/\epsilon$, as ϵ→0, the corresponding solutions should converge to a limiting population density that solves the corresponding “classical” PDE. We verify this in two examples; in the first the nonlocal equation is a reaction-diffusion equation with the “nonlocality” only appearing in the reaction term; in the second the nonlocal PDE is a special case of a nonlinear porous medium equation. These, in particular, capture the examples that we explored in Section 3.2.

#### 6.3.1 Reaction–diffusion equation limits

In this subsection we prove Proposition 2.15. *The conditions of the proposition are in force throughout this subsection*. The proof rests on a Feynman-Kac representation. We write ${\left(Z_{t}\right)}_{t\geq 0}$ for a diffusion with generator ${ℬ}^{*}$ and denote its transition density by $f_{t}(x,y)$. The first step is a regularity result for this density.

##### Lemma 6.8

*Fix* T>0. *There exists a constant* K=K(T)>0 such that, for any $x,\;y\in R^{d}$ *and* t∈[0,T],

(6.19) $$\int \left|f_{t}(x,z)-f_{t}(y,z)\right|dz\leq \frac{∥x-y∥}{\sqrt{t}}K.$$

##### Proof

We first use the Intermediate Value Theorem to obtain the bound

$$\int \left|f_{t}(x,z)-f_{t}(y,z)\right|dz\leq \int ∥x-y∥∥\nabla f_{t}(w,z)∥dz$$

where ∇ acts on the first coordinate only and w is in the line segment [x,y] joining x to y. Under our assumptions on b and C, equation (1.3) of Sheu [1991], gives existence of constants λ=λ(T)>0 and K such that,

$$∥\nabla f_{t}(w,z)∥\leq \frac{K}{\sqrt{t}}p_{\lambda t}(w,z),$$

where $p_{s}(x,y)$ is the Brownian transition density. Hence,

$$\int \left|f_{t}(x,z)-f_{t}(y,z)\right|dz\leq K\frac{∥x-y∥}{\sqrt{t}}\int p_{\lambda t}(w,z)dz=K\frac{∥x-y∥}{\sqrt{t}}.$$

□

##### Lemma 6.9

*Fix* T>0. Let $x,\;y\in R^{d},\;t\in [0,T]$, *and denote by* ${\left(Z_{t}^{y}\right)}_{t\geq 0}$ *and* ${\left(Z_{t}^{x}\right)}_{t\geq 0}$ *independent copies of the diffusion* ${\left(Z_{t}\right)}_{t\geq 0}$ *starting from* y *and* x *respectively. There exists a constant* K=K(T)>0 *such that,*

$$E\left[∥Z_{t}^{y}-Z_{t}^{x}∥\right]\leq K(\sqrt{t}+∥y-x∥).$$

##### Proof

First we write,

$$E\left[∥Z_{t}^{y}-Z_{t}^{x}∥\right]=\iint \;∥u-v∥f_{t}(y,u)f_{t}(x,v)dudv.$$

Under our regularity assumptions on C,b, using equation (1.2) of Sheu [1991], there exist constants K,λ=λ(T)>0 for which,

$$f_{t}\left(y,u\right)\leq Kp_{\lambda t}\left(y,u\right).$$

It then follows that,

(6.20) $$E\left[∥Z_{t}^{y}-Z_{t}^{x}∥\right]\leq \iint \;∥u-v∥K^{2}p_{\lambda t}(y,u)p_{\lambda t}(x,v)dvdu=K^{2}E\left[∥B_{\lambda t}^{y}-B_{\lambda t}^{x}∥\right],$$

where ${\left(B_{t}^{y}\right)}_{t\geq 0}$ and ${\left(B_{t}^{x}\right)}_{t\geq 0}$ are independent Brownian motions starting at y and x respectively. Using the triangle inequality, and writing ${\left(B_{t}^{0}\right)}_{t\geq 0}$ for a Brownian motion started from the origin,

(6.21) $$E\left[∥B_{\lambda t}^{y}-B_{\lambda t}^{x}∥\right]\leq ∥y-x∥+E\left[∥B_{2\lambda t}^{0}∥\right]\leq ∥y-x∥+C\sqrt{t}.$$

Substituting (6.21) in (6.20) gives the result. □

We use the representations of the solutions to equations (2.12) and (2.11) respectively:

(6.22) $$\varphi_{t}\left(x\right)=E_{x}\left[\varphi_{0}\left(Z_{t}\right)+\int_{0}^{t}\varphi_{s}\left(Z_{s}\right)F\left(\varphi_{s}\left(Z_{s}\right)\right)ds\right],$$

(6.23) $$\varphi_{t}^{\epsilon }(x)=E_{x}\left[\varphi_{0}\left(Z_{t}\right)+\int_{0}^{t}\varphi_{s}^{\epsilon }\left(Z_{s}\right)F\left(\rho_{F}^{\epsilon }*\varphi_{s}^{\epsilon }\left(Z_{s}\right)\right)ds\right],$$

from which

(6.24) $$\varphi_{t}\left(x\right)-\varphi_{t}^{\epsilon }\left(x\right)=E_{x}\left[\int_{0}^{t}\left(\varphi_{s}\left(Z_{s}\right)F\left(\varphi_{s}\left(Z_{s}\right)\right)-\varphi_{s}^{\epsilon }\left(Z_{s}\right)F\left(\rho_{\epsilon }*\varphi_{s}^{\epsilon }\left(Z_{s}\right)\right)\right)ds\right],$$

where $E_{x}$ denotes expectation for Z with $Z_{0}=x$. The key to our proof of Proposition 2.15 will be to replace $F\left(\varphi_{s}\left(Z_{s}\right)\right)$ by $F\left(\rho_{F}^{\epsilon }*\varphi_{s}\left(Z_{s}\right)\right)$ in this expression. We achieve this through three lemmas.

First we need a uniform bound on φ and $\varphi^{\epsilon }$.

##### Lemma 6.10

*For any* T>0 *there exists* $M=M\left(T,∥\varphi_{0}∥\right)>0$ *such that, for all* 0≤t≤T:

$$max\left\{{∥\varphi_{t}(\cdot )∥}_{\infty },{∥\varphi_{t}^{\epsilon }(\cdot )∥}_{\infty }\right\}<M.$$

##### Proof

Using that $\varphi_{0}$ and F are bounded above, from the representation (6.22), we have

$$\varphi_{t}(x)\leq {∥\varphi_{0}∥}_{\infty }+KE\left[\int_{0}^{t}\varphi_{s}\left(Z_{s}\right)ds\right].$$

In particular,

$${∥\varphi_{t}(\cdot )∥}_{\infty }\leq {∥\varphi_{0}∥}_{\infty }+K\int_{0}^{t}\;{∥\varphi_{s}(\cdot )∥}_{\infty }ds,$$

so, by Gronwall’s inequality,

$${∥\varphi_{t}(\cdot )∥}_{\infty }\leq {∥\varphi_{0}∥}_{\infty }exp(KT).$$

Similarly, ${∥\varphi_{t}^{\epsilon }(\cdot )∥}_{\infty }\leq {∥\varphi_{0}∥}_{\infty }exp(KT)$. □

We also need a continuity estimate for φ.

##### Lemma 6.11

*Let* T>0. *There exists a constant* $K=K\left(T,{∥\varphi_{0}∥}_{\infty }\right)>0$ *and* $\delta_{0}=\delta_{0}\left(T,{∥\varphi_{0}∥}_{\infty }\right)>0$ *such that for all* $0<\delta <\delta_{0}$ *and* 0≤t≤T,

$$∥x-y∥<\delta^{3}\Rightarrow \left|\varphi_{t}(x)-\varphi_{t}(y)\right|<K\delta .$$

##### Proof

First we need some notation. Fix T>0 and write M for the corresponding constant from Lemma 6.10. Let $∥F{∥}_{M}={sup}_{m\in [0,M]}\;|F(m)|$. We reserve $\hat{K}$ for the constant on the right hand side of equation (6.19) and $\tilde{K}$ for the constant in Lemma 6.9, and write $K_{\varphi_{0}}$ for the Lipschitz constant of $\varphi_{0}$. Set

$$\delta_{0}=min\left(\frac{1}{∥F{∥}_{M}^{2}},\frac{1}{Me\left(2∥F{∥}_{M}+\hat{K}\right)},\frac{1}{\tilde{K}K_{\varphi_{0}}+2∥F{∥}_{M}M},1\right).$$

In what follows we take $0<\delta <\delta_{0}$.

We first prove that the result holds if $t<\delta^{2}$. As before let $Z_{t}^{x}$ and $Z_{t}^{y}$ be independent copies of the diffusion $Z_{t}$ starting at x and y respectively. From our representation (6.22) and Lemma 6.10, we can write:

$$\left|\varphi_{t}(x)-\varphi_{t}(y)\right|\;\leq \left|E_{x}\left[\varphi_{0}\left(Z_{t}\right)\right]-E_{y}\left[\varphi_{0}\left(Z_{t}\right)\right]\right|+2∥F{∥}_{M}Mt\;\;\leq E\left[∥\varphi_{0}\left(Z_{t}^{x}\right)-\varphi_{0}\left(Z_{t}^{y}\right)|\right]+2∥F{∥}_{M}Mt\;\;\leq K_{\varphi_{0}}E\left[∥Z_{t}^{x}-Z_{t}^{y}∥\right]+2∥F{∥}_{M}Mt\;\;\leq \tilde{K}K_{\varphi_{0}}(\sqrt{t}+∥y-x∥)+2∥F{∥}_{M}Mt\;\;\leq \tilde{K}K_{\varphi_{0}}\left(\delta +\delta^{3}\right)+2∥F{∥}_{M}M\delta^{2}\leq (\tilde{K}K_{\varphi_{0}}+1)\delta ,$$

where we have used Lemma 6.9 in the fourth inequality and the definition of $\delta_{0}$ in the last inequality.

Suppose now that $\delta^{2}<t$. We will follow the pattern in Lemma 2.2 of Penington [2017]. First, note that by the Feynman-Kac formula we have an alternative representation for $\varphi_{t}(x)$: for any $t^{\prime }<t$,

$$\varphi_{t}\left(x\right)=E_{x}\left[\varphi_{t-t^{\prime }}\left(Z_{t^{\prime }}\right)\exp\left(\int_{0}^{t^{\prime }}F\left(\varphi_{t-s}\left(Z_{s}\right)\right)ds\right)\right].$$

Therefore, setting $t^{\prime }=\delta^{2}$ and using Lemma 6.10, for all z,

$$e^{-\delta^{2}∥F{∥}_{M}}E_{z}\left[\varphi_{t-\delta^{2}}\left(Z_{\delta^{2}}\right)\right]\leq \varphi_{t}\left(z\right)\leq e^{\delta^{2}∥F{∥}_{M}}E_{z}\left[\varphi_{t-\delta^{2}}\left(Z_{\delta^{2}}\right)\right].$$

We can then deduce that

(6.25) $$\varphi_{t}(x)-\varphi_{t}(y)\;\leq e^{\delta^{2}∥F{∥}_{M}}E_{x}\left[\varphi_{t-\delta^{2}}\left(Z_{\delta^{2}}\right)\right]-e^{-\delta^{2}∥F{∥}_{M}}E_{y}\left[\varphi_{t-\delta^{2}}\left(Z_{\delta^{2}}\right)\right]\;\;=e^{\delta^{2}∥F{∥}_{M}}\left(E_{x}\left[\varphi_{t-\delta^{2}}\left(Z_{\delta^{2}}\right)\right]-E_{y}\left[\varphi_{t-\delta^{2}}\left(Z_{\delta^{2}}\right)\right]\right)\;\;+(e^{\delta^{2}∥F{∥}_{M}}-e^{-\delta^{2}∥F{∥}_{M}})E_{y}\left[\varphi_{t-\delta^{2}}\left(Z_{\delta^{2}}\right)\right]\;\;\leq e^{\delta^{2}∥F{∥}_{M}}\left(E_{x}\left[\varphi_{t-\delta^{2}}\left(Z_{\delta^{2}}\right)\right]-E_{y}\left[\varphi_{t-\delta^{2}}\left(Z_{\delta^{2}}\right)\right]\right)+M(e^{\delta^{2}∥F{∥}_{M}}-e^{-\delta^{2}∥F{∥}_{M}}).$$

To bound the differences of the expected values in the last equation note that, by using again Lemma 6.10,

$$E_{x}\left[\varphi_{t-\delta^{2}}\left(Z_{\delta^{2}}\right)\right]-E_{y}\left[\varphi_{t-\delta^{2}}\left(Z_{\delta^{2}}\right)\right]\;\;=\int \varphi_{t-\delta^{2}}(z)\left(f_{\delta^{2}}(x,z)-f_{\delta^{2}}(y,z)\right)dz\;\;\leq M\int \left|f_{\delta^{2}}(x,z)-f_{\delta^{2}}(y,z)\right|dz\;\;\leq M\hat{K}\frac{∥x-y∥}{\delta }\leq M\hat{K}\delta^{2},$$

where we have used Lemma 6.8 and that $∥x-y∥<\delta^{3}$. Substituting in (6.25),

$$\varphi_{t}(x)-\varphi_{t}(y)\;\leq e^{\delta^{2}∥F{∥}_{M}}\left(M\hat{K}\delta^{2}+M-Me^{-2\delta^{2}∥F{∥}_{M}}\right)\;\;\leq e^{\delta^{2}∥F{∥}_{M}}\left(M\hat{K}\delta^{2}+2M\delta^{2}∥F{∥}_{M}\right)\;\;\leq e\left(M\hat{K}+2M∥F{∥}_{M}\right)\delta^{2}\leq \delta ,$$

where the last two inequalities follow from the definition of δ. Interchanging x and y yields the same bound for $\varphi_{t}(y)-\varphi_{t}(x)$, and the result follows. □

We proceed to control the difference between F(φ) and $F\left(\rho_{F}^{\epsilon }*\varphi \right)$. Note first that since $\rho_{F}\in L^{1}$,

$$I(\epsilon ):=\int_{\left\{∥y∥>\epsilon^{3/4}\right\}}\rho_{F}^{\epsilon }(y)dy=\int_{\left\{∥y∥>\epsilon^{-1/4}\right\}}\rho_{F}(y)dy\to 0\;\;\text{as}\;\epsilon \to 0.$$

##### Lemma 6.12

*Let* T>0. *There exists a constant* $C=C\left(T,{∥\varphi_{0}∥}_{\infty }\right)>0$ *such that, for all* 0≤t≤T, *for all* ϵ *small enough*,

(6.26) $${∥\varphi_{t}(\cdot )-\rho_{F}^{\epsilon }*\varphi_{t}(\cdot )∥}_{\infty }\leq C\left(I(\epsilon )+\epsilon^{1/4}\right).$$

Furthermore, there is a constant $\tilde{C}\left(T,{∥\varphi_{0}∥}_{\infty }\right)=\tilde{C}$ such that, for all 0≤t≤T,

(6.27) $${∥F\left(\varphi_{t}(\cdot )\right)-F\left(\rho_{F}^{\epsilon }*\varphi_{t}(\cdot )\right)∥}_{\infty }\leq \tilde{C}\left(I(\epsilon )+\epsilon^{1/4}\right).$$

##### Proof

Let $\epsilon <\delta_{0}^{4}$, with $\delta_{0}$ from Lemma 6.11. Then,

$$\left|\varphi_{t}(x)-\rho_{F}^{\epsilon }*\varphi_{t}(x)\right|\;\leq \int_{∥x-y∥>\epsilon^{3/4}}\;\rho_{F}^{\epsilon }(x-y)\left|\varphi_{t}(y)-\varphi_{t}(x)\right|dy\;\;+\int_{∥x-y∥\leq \epsilon^{3/4}}\;\rho_{F}^{\epsilon }(x-y)\left|\varphi_{t}(y)-\varphi_{t}(x)\right|dy\;\;\leq 2M\int_{∥x-y∥>\epsilon^{3/4}}\;\rho_{F}^{\epsilon }(x-y)dy+\int_{∥x-y∥\leq \epsilon^{3/4}}\;\rho_{F}^{\epsilon }(x-y)K\epsilon^{1/4}dy\;\;\leq 2MI(\epsilon )+K\epsilon^{1/4},$$

where we used the estimates of Lemma 6.10 and Lemma 6.11. This proves (6.26). For (6.27), let $L_{M}$ be the (uniform) Lipschitz constant of F on [0,M], with M still taken from Lemma 6.10. Then,

$${∥F\left(\varphi_{t}(\cdot )\right)-F\left(\rho_{F}^{\epsilon }*\varphi_{t}(\cdot )\right)∥}_{\infty }\left.\leq L_{M}∥\varphi_{t}(\cdot )\right)-\left(\rho_{F}^{\epsilon }*\varphi_{t}(\cdot )\right){∥}_{\infty }\;\;\leq L_{M}\left(2MI(\epsilon )+K\epsilon^{1/4}\right),$$

which proves (6.27). □

##### Proof [of Proposition 2.15]

Let ϵ be small enough that Lemma 6.12 holds. We use the notation $\hat{\delta }(\epsilon )$ for the quantity on the right hand side of (6.27). Then from the representation (6.24) and Lemma 6.12 we can write,

$$\left|\varphi_{t}(x)-\varphi_{t}^{\epsilon }(x)\right|\;\;\leq E_{x}\left[\int_{0}^{t}\;\left|\varphi_{s}\left(Z_{s}\right)F\left(\rho_{F}^{\epsilon }*\varphi_{s}\left(Z_{s}\right)\right)-\varphi_{s}^{\epsilon }\left(Z_{s}\right)F\left(\rho_{F}^{\epsilon }*\varphi_{s}^{\epsilon }\left(Z_{s}\right)\right)\right|ds\right]+Mt\hat{\delta }(\epsilon )\;\;\leq E_{x}\left[\int_{0}^{t}\;\left|F\left(\rho_{F}^{\epsilon }*\varphi_{s}^{\epsilon }\left(Z_{s}\right)\right)\right|\cdot \left|\varphi_{s}^{\epsilon }\left(Z_{s}\right)-\varphi_{s}\left(Z_{s}\right)\right|ds\right]\;\;+E_{x}\left[\int_{0}^{t}\;\left|\varphi_{s}\left(Z_{s}\right)\right|\left|F\left(\rho_{F}^{\epsilon }*\varphi_{s}^{\epsilon }\left(Z_{s}\right)\right)-F\left(\rho_{F}^{\epsilon }*\varphi_{s}\left(Z_{s}\right)\right)\right|ds\right]+Mt\hat{\delta }(\epsilon )\;\;\leq ∥F{∥}_{M}\int_{0}^{t}\;{∥\varphi_{s}^{\epsilon }(\cdot )-\varphi_{s}(\cdot )∥}_{\infty }ds+ML_{M}\int_{0}^{t}\;{∥\rho_{F}^{\epsilon }*\varphi_{s}^{\epsilon }(\cdot )-\rho_{F}^{\epsilon }*\varphi_{s}(\cdot )∥}_{\infty }ds+Mt\hat{\delta }(\epsilon )\;\;\leq \left(∥F{∥}_{M}+ML_{M}\right)\int_{0}^{t}\;{∥\varphi_{s}^{\epsilon }(\cdot )-\varphi_{s}(\cdot )∥}_{\infty }ds+Mt\hat{\delta }(\epsilon ),$$

where the second inequality is the triangle inequality, and the third is Lemma 6.10. An application of Gronwall’s inequality then yields,

$${∥\varphi_{t}^{\epsilon }(\cdot )-\varphi_{t}(\cdot )∥}_{\infty }\leq Mt\hat{\delta }(\epsilon )exp\left(t\left(∥F{∥}_{M}+ML_{M}\right)\right)\;\;\leq MT\hat{\delta }\left(\epsilon \right)\exp\left(T\left(∥F{∥}_{M}+ML_{M}\right)\right),$$

giving the result, since $\hat{\delta }\to 0$ as ϵ→0. □

#### 6.3.2 Porous Medium Equation

In this subsection we prove Proposition 2.18. To ease notation, we present the proof in d=1 (although we retain the notation ∇). However, to recall the dependence on ϵ we write $\rho^{\epsilon }$ for $\rho_{\gamma }$. It should be clear that it extends almost without change to higher dimensions.

Recall that we are concerned with non-negative solutions to the equation (2.13):

$$\partial_{t}\psi_{t}^{\epsilon }\left(x\right)=\Delta \left(\psi_{t}^{\epsilon }\rho^{\epsilon }*\psi_{t}^{\epsilon }\right)\left(x\right)+\psi_{t}^{\epsilon }\left(x\right)\left(1-\rho^{\epsilon }*\psi_{t}^{\epsilon }\left(x\right)\right).$$

and we assume that $\rho =\zeta *\overset{ˇ}{\zeta }$ with ζ a rapidly decreasing function and $\overset{ˇ}{\zeta }(x)=\zeta (-x)$. The example we have in mind is ζ (and therefore ρ) being the density of a mean zero Gaussian random variable. We shall prove that under the assumptions of Proposition 2.18, as ϵ→0, we have convergence to the solution to the porous medium equation with logistic growth, equation (1.2):

$$\partial_{t}\psi_{t}\left(x\right)=\Delta \left(\psi_{t}^{2}\right)\left(x\right)+\psi_{t}\left(x\right)\left(1-\psi_{t}\left(x\right)\right).$$

We work on the time interval [0,T]. We will require a lower bound on $\int \psi_{t}^{\epsilon }(x)log\psi_{t}^{\epsilon }(x)dx$ which we record as a lemma.

##### Lemma 6.13

*Suppose that there exists* λ∈(0,1) *and* C<∞, *both independent of* ϵ, *such that* $\int exp(\lambda |x|)\psi_{0}^{\epsilon }(x)dx<C$. *Then there exists a constant* K<∞, *independent of* ϵ, *such that* $\int \psi_{t}^{\epsilon }(x)log\psi_{t}^{\epsilon }(x)dx>-K$ *for all* t∈[0,T].

##### Proof

First observe that, since xlogx is bounded below, $\int_{-1}^{1}\;\psi_{t}^{\epsilon }(x)log\psi_{t}^{\epsilon }(x)dx$ is bounded below, and recall that $\psi_{t}^{\epsilon }(x)\geq 0$.

Now consider

(6.28) $$\frac{d}{dt}\int exp(\lambda x)\psi_{t}^{\epsilon }(x)dx=\int exp(\lambda x)\Delta \left(\psi_{t}^{\epsilon }\rho^{\epsilon }*\psi_{t}^{\epsilon }\right)(x)dx\;\;+\int exp(\lambda x)\psi_{t}^{\epsilon }(x)\left(1-\rho^{\epsilon }*\psi_{t}^{\epsilon }(x)\right)dx\;\;=\int \left(\lambda^{2}-1\right)exp(\lambda x)\psi_{t}^{\epsilon }(x)\rho_{\epsilon }*\psi_{t}^{\epsilon }(x)dx+\int exp(\lambda x)\psi_{t}^{\epsilon }(x)dx\;\;\leq \int \exp\left(\lambda x\right)\psi_{t}^{\epsilon }\left(x\right)dx,$$

and so, by Gronwall’s inequality, $\int exp(\lambda x)\psi_{t}^{\epsilon }(x)dx$ is uniformly bounded on [0,T]. In particular, combining with the Mean Value Theorem, we find

$$\int_{x}^{x+1}\psi_{t}^{\epsilon }(y)dy\leq Cexp(-\lambda x),$$

where the constant C is independent of x≥1. A fortiori,

(6.29) $$\int_{x}^{x+1}\psi_{t}^{\epsilon }(y)1_{\psi_{t}^{\epsilon }(y)\leq 1}dy\leq Cexp(-\lambda x).$$

Now the function $\psi \mapsto 1_{0\leq \psi \leq 1}\psi |log\psi |$ is concave, and so using Jensen’s inequality and (6.29),

$$\int_{x}^{x+1}\psi_{t}^{\epsilon }(y)\left|log\psi_{t}^{\epsilon }(y)\right|1_{\psi_{t}^{\epsilon }(y)\leq 1}dy\leq C^{'}xexp(-\lambda x).$$

Evidently a symmetric argument applies for x≤-1. Summing over x, and using that $\psi log\psi \geq -\psi |log\psi |1_{\psi \leq 1}$, we find

$$\int \psi_{t}^{\epsilon }(x)\log\psi_{t}^{\epsilon }(x)dx\geq -C^{\prime \prime }\sum_{x=1}^{\infty }x\exp(-\lambda x)>-K>-\infty ,$$

as required. □

##### Proof [Proof of Proposition 2.18]

First observe that

$$\int \psi_{t}^{\epsilon }(x)\rho^{\epsilon }*\psi_{t}^{\epsilon }(x)dx\;=\int \int \int \psi_{t}^{\epsilon }(x)\psi_{t}^{\epsilon }(x-y)\zeta^{\epsilon }(y-z){\overset{ˇ}{\zeta }}^{\epsilon }(z)dzdydx\;\;=\int \int \int \psi_{t}^{\epsilon }\left(\tilde{x}-\tilde{z}\right)\psi_{t}^{\epsilon }\left(\tilde{x}-\tilde{y}\right)\zeta^{\epsilon }\left(\tilde{y}\right)\zeta^{\epsilon }\left(\tilde{z}\right)d\tilde{z}d\tilde{y}d\tilde{x}=\int {\left(\zeta^{\epsilon }*\psi_{t}^{\epsilon }\left(x\right)\right)}^{2}dx,$$

where we have set $\tilde{x}=x-z,\;\tilde{y}=y-z,\;\tilde{z}=-z$.

Now note that

$$\frac{d}{dt}\int \psi_{t}^{\epsilon }(x)dx\;=\int \Delta \left(\psi_{t}^{\epsilon }\rho^{\epsilon }*\psi_{t}^{\epsilon }\right)(x)dx+\int \psi_{t}^{\epsilon }(x)\left(1-\rho^{\epsilon }*\psi_{t}^{\epsilon }(x)\right)dx\;\;=\int \psi_{t}^{\epsilon }\left(x\right)dx-\int {\left(\zeta^{\epsilon }*\psi_{t}^{\epsilon }\left(x\right)\right)}^{2}dx.$$

Thus, Gronwall’s inequality implies that $\int \psi_{t}^{\epsilon }(x)dx$ is uniformly bounded above in ϵ and t∈[0,T]. Note that this also then gives a uniform bound on the rate of change of $\int \psi_{t}^{\epsilon }(x)dx$, and since we are working on [0,T] this will be enough to give continuity in time of the $L^{1}$ norm of the limit when we pass to a convergent subsequence.

Now consider

(6.30) $$\frac{d}{dt}\int \psi_{t}^{\epsilon }log\psi_{t}^{\epsilon }dx=\;\int \left(1+log\psi_{t}^{\epsilon }\right)\left[\Delta \left(\psi_{t}^{\epsilon }\rho^{\epsilon }*\psi_{t}^{\epsilon }\right)+\psi_{t}^{\epsilon }\left(1-\rho^{\epsilon }*\psi_{t}^{\epsilon }\right)\right]dx\;\;=\int \left(1+log\psi_{t}^{\epsilon }\right)\left[\nabla \left(\psi_{t}^{\epsilon }\nabla \left(\rho^{\epsilon }*\psi_{t}^{\epsilon }\right)+\nabla \psi_{t}^{\epsilon }\rho^{\epsilon }*\psi_{t}^{\epsilon }\right)+\psi_{t}^{\epsilon }\left(1-\rho^{\epsilon }*\psi_{t}^{\epsilon }\right)\right]dx\;\;=\;\int \left[-\frac{\nabla \psi_{t}^{\epsilon }}{\psi_{t}^{\epsilon }}\left(\psi_{t}^{\epsilon }\nabla \left(\rho^{\epsilon }*\psi_{t}^{\epsilon }\right)+\nabla \psi_{t}^{\epsilon }\rho^{\epsilon }*\psi_{t}^{\epsilon }\right)+\left(1+log\psi_{t}^{\epsilon }\right)\psi_{t}^{\epsilon }\left(1-\rho^{\epsilon }*\psi_{t}^{\epsilon }\right)\right]dx\;\;=\;-\int {\left(\nabla \left(\zeta^{\epsilon }*\psi_{t}^{\epsilon }\right)\right)}^{2}dx-\int {\left(\nabla \psi_{t}^{\epsilon }\right)}^{2}\frac{\rho^{\epsilon }*\psi_{t}^{\epsilon }}{\psi_{t}^{\epsilon }}dx\;\;+\int \left[\psi_{t}^{\epsilon }+\psi_{t}^{\epsilon }log\psi_{t}^{\epsilon }\left(1-\rho^{\epsilon }*\psi_{t}^{\epsilon }\right)-\psi_{t}^{\epsilon }\rho^{\epsilon }*\psi_{t}^{\epsilon }\right]dx\;\;=\;-\int {\left(\nabla \left(\zeta^{\epsilon }*\psi_{t}^{\epsilon }\right)\right)}^{2}dx-\int {\left(\nabla \psi_{t}^{\epsilon }\right)}^{2}\frac{\rho^{\epsilon }*\psi_{t}^{\epsilon }}{\psi_{t}^{\epsilon }}dx-\int {\left(\zeta^{\epsilon }*\psi_{t}^{\epsilon }\right)}^{2}dx\;\;+\int \left[\psi_{t}^{\epsilon }+\psi_{t}^{\epsilon }log\psi_{t}^{\epsilon }\left(1-\rho^{\epsilon }*\psi_{t}^{\epsilon }\right)\right]dx.$$

The first three terms are negative; and we already saw that the $L^{1}$ norm of $\psi_{t}^{\epsilon }$ is uniformly bounded. Moreover, since $\psi_{t}^{\epsilon }log\psi_{t}^{\epsilon }$ is uniformly bounded below and $\int \rho^{\epsilon }(x)dx=1$,

$$-\int \psi_{t}^{\epsilon }\log\psi_{t}^{\epsilon }\rho^{\epsilon }*\psi_{t}^{\epsilon }dx\leq C\int \rho^{\epsilon }*\psi_{t}^{\epsilon }dx=C\int \psi_{t}^{\epsilon }dx.$$

From this and (6.30), we see immediately that $\int \psi_{t}^{\epsilon }log\psi_{t}^{\epsilon }dx$ is uniformly bounded above in ϵ and t∈[0,T]. Combining with Lemma 6.13, we deduce that we have a uniform bound on $\int \psi_{s}^{\epsilon }(x)\left|log\psi_{s}^{\epsilon }(x)\right|dx$. From (6.30), this in turn means that both $\int_{0}^{t}\;\int {\left(\zeta^{\epsilon }*\psi_{s}^{\epsilon }(x)\right)}^{2}dxds$ and $\int_{0}^{t}\;\int {\left(\nabla \left(\zeta^{\epsilon }*\psi_{s}^{\epsilon }(x)\right)\right)}^{2}dxds$ are uniformly bounded in ϵ and t∈[0,T].

We shall next show that $\zeta^{\epsilon }*\psi_{t}^{\epsilon }$ solves (1.2) up to a remainder of order ϵ. First observe that

(6.31) $$\int \Delta \left(\left(\rho^{\epsilon }*\psi_{t}^{\epsilon }\right)\psi_{t}^{\epsilon }\right)\phi dx=-\int \nabla \left(\rho^{\epsilon }*\psi_{t}^{\epsilon }\right)\psi_{t}^{\epsilon }\nabla \phi dx-\int \rho^{\epsilon }*\psi_{t}^{\epsilon }\nabla \psi_{t}^{\epsilon }\nabla \phi dx.$$

We would like to show that this is close to $\int {\left(\zeta^{\epsilon }*\psi_{t}^{\epsilon }\right)}^{2}\Delta \phi dx$. For the first term

(6.32) $$\int \nabla \left(\rho^{\epsilon }*\psi_{t}^{\epsilon }\right)\psi_{t}^{\epsilon }\nabla \phi dx=\;\int \int \int \nabla \psi_{t}^{\epsilon }(x-y)\zeta^{\epsilon }(y-z){\overset{ˇ}{\zeta }}^{\epsilon }(z)\psi_{t}^{\epsilon }(x)\nabla \phi (x)dzdydx\;\;=\;\int \int \int \nabla \psi_{t}^{\epsilon }(\tilde{x}-\tilde{y})\zeta^{\epsilon }(\tilde{y})\zeta^{\epsilon }(\tilde{z})\psi_{t}^{\epsilon }(\tilde{x}-\tilde{z})\nabla \phi (\tilde{x}-\tilde{z})d\tilde{z}d\tilde{y}d\tilde{x}\;\;=\;\int \left(\nabla \zeta^{\epsilon }*\psi_{t}^{\epsilon }\right)\left(\zeta^{\epsilon }*\left(\psi_{t}^{\epsilon }\nabla \phi \right)\right)dx\;\;=\frac{1}{2}\int \nabla \left({\left(\zeta^{\epsilon }*\psi_{t}^{\epsilon }\right)}^{2}\right)\nabla \phi dx\;\;+\int \nabla \left(\zeta^{\epsilon }*\psi_{t}^{\epsilon }\right)\left[\zeta^{\epsilon }*\left(\psi_{t}^{\epsilon }\nabla \phi \right)-\nabla \phi \left(\zeta^{\epsilon }*\psi_{t}^{\epsilon }\right)\right]dx,$$

where, as before, we have substituted $\tilde{x}=x-z,\;\tilde{y}=y-z,\;\tilde{z}=-z$. To control the term (6.32) we use the Intermediate Value Theorem to see that

$$\left|\int \left[\psi_{t}^{\epsilon }(x-y)\nabla \phi (x-y)\zeta^{\epsilon }(y)-\nabla \phi (x)\psi_{t}^{\epsilon }(x-y)\zeta^{\epsilon }(y)\right]dy\right|\;\;\leq C∥\Delta \phi {∥}_{\infty }\int \psi_{t}^{\epsilon }\left(x-y\right)y\zeta^{\epsilon }\left(y\right)dy.$$

Since ζ∈𝒮(R), the integral in this expression is 𝒪(ϵ).

Similarly, for the second term in (6.31),

(6.33) $$\int \left(\rho^{\epsilon }*\psi_{t}^{\epsilon }\right)\nabla \psi_{t}^{\epsilon }\nabla \phi dx=\;\int \int \int \psi_{t}^{\epsilon }(x-y)\zeta^{\epsilon }(y-z){\overset{ˇ}{\zeta }}^{\epsilon }(z)\nabla \psi_{t}^{\epsilon }(x)\nabla \phi (x)dzdydx\;\;=\;\int \int \int \psi_{t}^{\epsilon }(\tilde{x}-\tilde{y})\zeta^{\epsilon }(\tilde{y})\zeta^{\epsilon }(\tilde{z})\nabla \psi_{t}^{\epsilon }(\tilde{x}-\tilde{z})\nabla \phi (\tilde{x}-\tilde{z})d\tilde{z}d\tilde{y}d\tilde{x}\;\;=\;\int \left(\zeta^{\epsilon }*\psi_{t}^{\epsilon }\right)\left(\zeta^{\epsilon }*\left(\nabla \psi_{t}^{\epsilon }\nabla \phi \right)\right)dx\;\;=\frac{1}{2}\int \nabla \left({\left(\zeta^{\epsilon }*\psi_{t}^{\epsilon }\right)}^{2}\right)\nabla \phi dx\;\;+\int \left(\zeta^{\epsilon }*\psi_{t}^{\epsilon }\right)\left[\zeta^{\epsilon }*\left(\nabla \psi_{t}^{\epsilon }\nabla \phi \right)-\nabla \phi \left(\nabla \zeta^{\epsilon }*\psi_{t}^{\epsilon }\right)\right]dx,$$

and (6.33) is controlled in the same way as (6.32): using the Intermediate Value Theorem,

$$|\int \left[\nabla \psi_{t}^{\epsilon }(x-y)\nabla \phi (x-y)\zeta^{\epsilon }(y)-\nabla \phi (x)\nabla \psi_{t}^{\epsilon }(x-y)\zeta^{\epsilon }(y)\right]dy|\;\;\leq C∥\Delta \phi {∥}_{\infty }|\int \nabla \psi_{t}^{\epsilon }\left(x-y\right)y\zeta^{\epsilon }\left(y\right)dy|,$$

which again is 𝒪(ϵ).

We now have the ingredients that we need. The calculations above yield both a uniform (in ϵ) bound on $\zeta^{\epsilon }*\psi_{t}^{\epsilon }$ in $L^{1}\cap L^{2}([0,T]\times R)$, and that

(6.34) $$\int \psi_{t}^{\epsilon }(x)\phi (x)dx-\int \psi_{0}^{\epsilon }(x)\phi (x)dx\;=\int_{0}^{t}\;\int {\left(\zeta^{\epsilon }*\psi_{s}^{\epsilon }(x)\right)}^{2}\Delta \phi (x)dx\;\;+\int_{0}^{t}\;\int \zeta^{\epsilon }*\psi_{s}^{\epsilon }(x)\left(1-\zeta^{\epsilon }*\psi_{s}^{\epsilon }(x)\right)\phi (x)dx+𝒪(\epsilon )$$

(for sufficiently regular ϕ). Since $\int \psi_{t}^{\epsilon }(x)\phi (x)dx-\int \zeta^{\epsilon }*\psi_{t}^{\epsilon }(x)\phi (x)dx$ is order ϵ, if we replace $\psi^{\epsilon }$ by $\zeta^{\epsilon }*\psi^{\epsilon }$ on the left hand side, then (6.34) says that $\zeta^{\epsilon }*\psi^{\epsilon }$ solves (2.14) weakly up to order ϵ. Therefore, $\zeta^{\epsilon }*\psi^{\epsilon }$ converges weakly to ψ in $L^{1}$, where ψ is the (unique) solution to equation (2.14) and, so, therefore, does $\psi^{\epsilon }$. In fact, strong convergence, that is $\int |\psi^{\epsilon }-\psi |\phi dx\to 0$, follows from the uniform integrability of $\psi^{\epsilon }$ that we can deduce from the uniform control of $\int \psi^{\epsilon }\left|log\psi^{\epsilon }\right|dx$ that we proved above. □

## 7 Simultaneous scaling with interaction distance

In this section we prove Theorem 2.20, which proves convergence in the case that the width of the interaction kernel $\rho_{F}$ simultaneously scales along with the parameters θ and N, in the special case in which $r\equiv 1\equiv \gamma ,\;q_{\theta }(x,dy)$ is isotropic with zero mean, the kernel $\rho_{F}$ is Gaussian, and the scaling limit is a reaction-diffusion equation.

*To simplify notation, in this section we shall write*

$$\rho_{\epsilon }*\eta (x)=\rho_{F}^{\epsilon }*\eta (x)=\left\langle p_{\epsilon^{2}}(x,y),\eta (dy)\right\rangle ,$$

where $p_{t}(x,y)$ denotes the heat semigroup. The assumptions of Theorem 2.20 will be in force throughout, in particular,

(7.1) $$\epsilon^{2}\theta \to \infty ,\;\text{and}\;\frac{\theta }{N\epsilon^{d}}\to 0.$$

*That* N,θ→∞ *and* ϵ→0 *simultaneously will be implicit, so for example if we write* ${lim}_{\epsilon \to 0}\;$, it should be understood that θ,N→∞ *in such a way that (7.1) is satisfied. Moreover, where there is no risk of confusion, except where it is helpful for emphasis, we suppress dependence of* η *on* N.

The first part of the proof mirrors that of Theorem 2.10: in Subsection 7.1 we establish bounds on the moments of $\rho_{\epsilon }*\eta_{t}(x)$ that are sufficient to imply tightness and then apply standard results on convergence of Markov processes from Ethier and Kurtz [1986]. The challenge comes in identifying the limit points. This is much more intricate than the case in which we do not scale the interaction kernel, as weak convergence will no longer be sufficient to guarantee the form of the nonlinear terms in the limiting equation. Identification of the limit will rest on regularity inherited from continuity estimates for a random walk with Gaussian jumps which we prove in Subsection 7.2, before identifying the limit points in Subsection 7.3.

### 7.1 Moment bounds for $\rho_{\epsilon }*\eta$

Let us write ${ℒ}^{\theta }f(x):=\theta \int (f(y)-f(x))q_{\theta }(x,y)dy$ where $q_{\theta }$ is a Gaussian kernel of mean 0 and variance 1/θ. We note that ${ℒ}^{\theta }$ is the generator of a continuous (time and space) random walk, which makes jumps of mean 0 and variance 1/θ at rate θ. In what follows we write $\psi_{t}^{\epsilon ,x}(y)$ for the solution of

(7.2) $$\partial_{t}\psi_{t}^{\epsilon ,x}={ℒ}^{\theta }\psi_{t}^{\epsilon ,x},$$

with initial condition $\psi_{0}^{\epsilon ,x}\left(y\right)=\rho_{\epsilon }\left(y-x\right)=p_{\epsilon^{2}}\left(x,y\right).$

To see why $\psi_{t}^{\epsilon ,x}$ is useful, first note that for any time-dependent function $\phi_{t}(x)$ with time derivative ${\dot{\phi }}_{t}\left(x\right)=\partial_{t}\phi_{t}\left(x\right),$

(7.3) $$\left\langle \phi_{t}(x),\eta_{t}(dx)\right\rangle =\left\langle \phi_{0}(x),\eta_{0}(dx)\right\rangle +M_{t}(\phi )+\int_{0}^{t}\;\langle {ℒ}^{\theta }\phi_{s}(x)+{\dot{\phi }}_{s}(x),\eta_{s}(dx)\rangle ds\;\;+\int_{0}^{t}\;\left\langle \phi_{s}(x)F\left(x,\eta_{s}\right),\eta_{s}(dx)\right\rangle ds,$$

where $M_{t}(\phi )$ is a martingale (with respect to the natural filtration) with angle bracket process given by (2.4) with f replaced by $\phi_{s}(\cdot )$. So, taking $\phi_{s}(\cdot )=\psi_{t-s}^{\epsilon ,x}(\cdot )$ for 0≤s≤t,

(7.4) $$\rho_{\epsilon }*\eta_{t}(x)\;=\left\langle \psi_{0}^{\epsilon ,x}(y),\eta_{t}(dy)\right\rangle \;\;=\left\langle \psi_{t}^{\epsilon ,x}(y),\eta_{0}(dy)\right\rangle +\int_{0}^{t}\;\left\langle \psi_{t-s}^{\epsilon ,x}(y)F\left(\rho_{\epsilon }*\eta_{s}(y)\right),\eta_{s}(dy)\right\rangle ds+M_{t}\left(x\right),$$

where $M_{t}(x)$ has mean zero and a second moment we can easily write down.

#### Lemma 7.1

*Fix* t>0, *let* $(\Pi (s){)}_{s\geq 0}$ *be a rate one Poisson process, and let* T(t)=Π(θt)/θ. *Then*

$$\psi_{t}^{\epsilon ,x}(y)=E\left[p_{\epsilon^{2}+T(t)}(x,y)\right],$$

and, moreover, since under our assumptions $\theta \epsilon^{2}$ is bounded below, there is a C independent of ϵ or t such that

$${∥\psi_{t}^{\epsilon ,x}∥}_{\infty }\leq \frac{C}{{\left(\epsilon^{2}+t\right)}^{d/2}}.$$

#### Proof

The first claim is immediate from the definition of the random walk with generator ${ℒ}^{\theta }$.

For the second claim, first define τ(t)=T(t)-t. Since if $\tau (t)\geq -\left(\epsilon^{2}+t\right)/2$, then $1/\left(\epsilon^{2}+T(t)\right)\leq 2/\left(\epsilon^{2}+t\right)$, while $\epsilon^{2}+T(t)\geq \epsilon^{2}$ always, partitioning over $\left\{\tau (t)\geq -\left(\epsilon^{2}+t\right)/2\right\}$ and its complement,

(7.5) $${∥\psi^{\epsilon ,x}∥}_{\infty }\;=E\left[\frac{1}{{\left(2\pi \left(\epsilon^{2}+T(t)\right)\right)}^{d/2}}\right]\;\;\leq \frac{C}{{\left(\epsilon^{2}+t\right)}^{d/2}}+\frac{C}{\epsilon^{d}}P\left\{\tau \left(t\right)<-\left(\epsilon^{2}+t\right)/2\right\}.$$

Now, observe that since $E\left[e^{-\Pi (\theta t)}\right]=exp\left(-\theta t\left(1-e^{-1}\right)\right)$, by Markov’s inequality,

(7.6) $$P\left\{\tau (t)<-\frac{\epsilon^{2}+t}{2}\right\}\;=P\left\{e^{-\Pi (\theta t)}>e^{-\theta \left(t-\epsilon^{2}\right)/2}\right\}\;\;\leq \frac{E[exp(-\Pi (\theta t))]}{exp\left(-\theta \left(t-\epsilon^{2}\right)/2\right)}\;\;=\frac{exp\left(-\theta t\left(1-e^{-1}\right)\right)}{exp\left(-\theta \left(t-\epsilon^{2}\right)/2\right)}\;\;=\exp\left\{-\chi \theta t-\frac{\theta \epsilon^{2}}{2}\right\},$$

where $\chi =1/2-e^{-1}>0$. The second term in (7.5) is therefore bounded by

$$C{\left(1+\frac{t}{\epsilon^{2}}\right)}^{d/2}e^{-\chi \theta t}\frac{1}{{\left(\epsilon^{2}+t\right)}^{d/2}}e^{-\epsilon^{2}\theta /2}.$$

Now observe that the derivative (with respect to t) of $e^{-\chi \theta t}{\left(1+t/\epsilon^{2}\right)}^{d/2}$ is

$$\left(\frac{d}{2\epsilon^{2}}-\left(1+\frac{t}{\epsilon^{2}}\right)\chi \theta \right){\left(1+\frac{t}{\epsilon^{2}}\right)}^{d/2-1}e^{-\chi \theta t},$$

which is negative if $\theta \left(\epsilon^{2}+t\right)>d/2\chi$. At the maximum, $\left(1+t/\epsilon^{2}\right)=d/\left(2\chi \theta \epsilon^{2}\right)$, and so this quantity is bounded uniformly over not only t but also ϵ (since we’ve assumed that $\theta \epsilon^{2}$ is bounded below). Therefore, we have the bound

(7.7) $$\frac{1}{\epsilon^{d}}P\left\{\tau (t)<-\left(\epsilon^{2}+t\right)/2\right\}\leq \frac{C}{{\left(\epsilon^{2}+t\right)}^{d/2}}e^{-\epsilon^{2}\theta /2}.$$

Substituting this into (7.5) yields the result. □

#### Lemma 7.2

*Let* ${\left\{{ℱ}_{t}\right\}}_{t\geq 0}$ *denote the natural filtration. Under the assumptions of* Theorem 2.20, *for each* T∈[0,∞), *and* k∈N, *there exist constants* C=C(k,T) *and* $\tilde{C}=\tilde{C}(k,T)$, *independent of* ϵ, *such that for all* $x\in R^{d}$ *and all* u,t∈[0,T] *with* u<t,

(7.8) $$E\left[{\left(\rho_{\epsilon }*\eta_{t}(x)\right)}^{k}|{ℱ}_{u}\right]\leq C{\left\langle \psi_{t-u}^{\epsilon ,x}(z),\eta_{u}(dz)\right\rangle }^{k}+C\frac{\theta }{N\epsilon^{d}}\left\langle \psi_{t-u}^{\epsilon ,x}(z),\eta_{u}(dz)\right\rangle ;$$

*and*

(7.9) $$E\left[\int_{u}^{t}{\left\langle \psi_{t-s}^{\epsilon ,x}(z),\eta_{s}(dz)\right\rangle }^{k-1}\left\langle \psi_{t-s}^{\epsilon ,x}(z)\right|\right.\left.F\left(\rho_{\epsilon }*\eta_{s}(z)\right)\left|,\eta_{s}(dz)\right\rangle ds|{ℱ}_{u}\right]\;\;\leq \tilde{C}{\left\langle \psi_{t-u}^{\epsilon ,x}\left(z\right),\eta_{u}\left(dz\right)\right\rangle }^{k}+\tilde{C}\frac{\theta }{N\epsilon^{d}}\left\langle \psi_{t-u}^{\epsilon ,x}\left(z\right),\eta_{u}\left(dz\right)\right\rangle ;$$

where the function $\psi_{t}^{\epsilon ,x}(\cdot )$ was defined in (7.2). In particular, under the assumptions of Theorem 2.20, the expected values of the quantities on the right hand side of (7.8) and (7.9) are both integrable with respect to Lebesgue measure.

#### Proof

To simplify our expressions, we shall consider the case u=0, but the proof goes through unchanged for other values of u.

We proceed by induction. Taking expectations in (7.4), using that F is bounded above, and applying Gronwall’s inequality to $\left\langle \psi_{t-s}^{\epsilon ,x},\eta_{s}\right\rangle$ we obtain $E\left[\left\langle \psi_{0}^{\epsilon ,x},\eta_{t}\right\rangle \right]\leq CE\left[\left\langle \psi_{t}^{\epsilon ,x},\eta_{0}\right\rangle \right]$, which implies (7.8) in the case k=1. Moreover, rearranging (7.4) we find

(7.10) $$-\int_{0}^{t}\left\langle \psi_{t-s}^{\epsilon ,x}\left(y\right)F\left(\rho_{\epsilon }*\eta_{s}\left(y\right),\eta_{s}\left(dy\right)\right\rangle ds=\left\langle \psi_{t}^{\epsilon ,x}\left(y\right),\eta_{0}\left(dy\right)\right\rangle -\left\langle \psi_{0}^{\epsilon ,x}\left(y\right),\eta_{t}\left(dy\right)\right\rangle +M_{t}\left(x\right)\right.,$$

and taking expectations again, since $\left\langle \psi_{0}^{\epsilon ,x},\eta_{t}\right\rangle >0$, and $M_{0}(x)=0$, this yields

$$E\left[-\int_{0}^{t}\left\langle \psi_{t-s}^{\epsilon ,x}(y)F\left(\rho_{\epsilon }*\eta_{s}(y)\right),\eta_{s}(dy)\right\rangle ds|{ℱ}_{0}\right]\leq \left\langle \psi_{t}^{\epsilon ,x}\left(y\right),\eta_{0}\left(dy\right)\right\rangle .$$

Since F is bounded above, there exists a constant K such that |F|≤K-F and so combined with the bound on $E\left[\left\langle \psi_{0}^{\epsilon ,x}(y),\eta_{t}(dy)\right\rangle \right]$ just obtained, this in turn yields

$$E\left[\int_{0}^{t}\left\langle \psi_{t-s}^{\epsilon ,x}(y)\left|F\left(\rho_{\epsilon }*\eta_{s}(y)\right)\right|,\eta_{s}(dy)\right\rangle ds|{ℱ}_{0}\right]\leq \tilde{C}\left\langle \psi_{t}^{\epsilon ,x}\left(y\right),\eta_{0}\left(dy\right)\right\rangle ,$$

which is (7.9) in the case k=1.

Now suppose that we have established (7.8) and (7.9) for all exponents j<k. First we apply the generator ${𝒫}^{N}$ of our scaled population process to functions of the form $\langle f,\eta \rangle^{k}$. Recalling that each jump of the process involves the birth or death of a single individual, and so increments ⟨f,η⟩ by ±f/N at the location of that individual and that r≡γ≡1, we find

(7.11) $$\begin{array}{l} {𝒫}^{N}\left({\langle f,\eta \rangle }^{k}\right)=\langle \int \theta N\sum_{j=1}^{k}\left(\begin{array}{l} k\\ j \end{array}\right)\frac{f{\left(y\right)}^{j}}{N^{j}}{\langle f,\eta \rangle }^{k-j}q_{\theta }(x,dy),\eta (dx)\rangle \\ \;\;\;\;\;\;\;\;\;\;\;\;\;\;\;\;\;\;\;\;\;\;\;\;\;\;\;\;\;\;\;+\langle \theta N\left(1-\frac{F\left(\rho_{\epsilon }*\eta (x)\right)}{\theta }\right)\sum_{j=1}^{k}\left(\begin{array}{c} k\\ j \end{array}\right){\left(-1\right)}^{j}\frac{f{\left(x\right)}^{j}}{N^{j}}{\langle f,\eta \rangle }^{k-j},\eta (dx)\rangle . \end{array}$$

Mimicking what we did above, we set $f(\cdot )=\psi_{t}^{\epsilon ,x}(\cdot )$ and write

(7.12) $$E\left[{\left\langle \psi_{0}^{\epsilon ,x},\eta_{t}\right\rangle }^{k}|{ℱ}_{0}\right]={\left\langle \psi_{t}^{\epsilon ,x}(y),\eta_{0}(dy)\right\rangle }^{k}+E\left[\int_{0}^{t}\;{𝒫}^{N}\left({\left\langle \psi_{t-s}^{\epsilon ,x}(y),\eta_{s}(dy)\right\rangle }^{k}\right)ds\right.\;\;\left.\;-\int_{0}^{t}\;\left\langle k{\dot{\psi }}_{t-s}^{\epsilon ,x}(y),\eta_{s}(dy)\right\rangle {\left\langle \psi_{t-s}^{\epsilon ,x}(y),\eta_{s}(dy)\right\rangle }^{k-1}ds|{ℱ}_{0}\right].$$

Since ${\dot{\psi }}_{s}^{\epsilon ,x}={ℒ}_{\theta }\psi_{s}^{\epsilon ,x}$, the j=1 terms from ${𝒫}^{N}\left({\left\langle \psi_{t-s}^{\epsilon ,x}(y),\eta_{s}(dy)\right\rangle }^{k}\right)$ combines with the last term in (7.12) to yield

$$\int_{0}^{t}k{\left\langle \psi_{t-s}^{\epsilon ,x},\eta \right\rangle }^{k-1}\left\langle F\left(\rho_{\epsilon }*\eta_{s}(y)\right)\psi_{t-s}^{\epsilon ,x}(y),\eta_{s}(dy)\right\rangle ds.$$

As for the remaining terms, using (from Lemma 7.1) that ${sup}_{s}\;{∥\psi_{s}^{\epsilon ,x}(\cdot )∥}_{\infty }=C/\epsilon^{d},\;N\epsilon^{d}>1$, and our inductive hypothesis, we find

$$\begin{array}{ll} & E\left[\left\langle \int_{0}^{t}\;\theta N\sum_{j=2}^{k}\;\left(\begin{array}{c} k\\ j \end{array}\right)\int \frac{\psi_{t-s}^{\epsilon ,x}(z{)}^{j}}{N^{j}}{\left\langle \psi_{t-s}^{\epsilon ,x},\eta_{s}\right\rangle }^{k-j}q_{\theta }(y,dz),\eta_{s}(dy)\right\rangle ds\right.\\ & \left.\;+\left\langle \int_{0}^{t}\;\theta N\sum_{j=2}^{k}\;\left(\begin{array}{c} k\\ j \end{array}\right)\frac{\psi_{t-s}^{\epsilon ,x}(y{)}^{j}}{N^{j}}{\left\langle \psi_{t-s}^{\epsilon ,x},\eta_{s}\right\rangle }^{k-j}(-1{)}^{j}\left(1-\frac{F\left(\rho_{\epsilon }*\eta_{s}(y)\right)}{\theta }\right),\eta_{s}(dy)\right\rangle ds|{ℱ}_{0}\right]\\ & \;\leq CE\left[\left\langle \int_{0}^{t}\sum_{j=2}^{k}\frac{\theta }{N\epsilon^{d}}\left(\frac{1}{{\left(N\epsilon^{d}\right)}^{j-2}}\right)\left\langle \psi_{t-s}^{\epsilon ,x}(y)\left(2+\frac{\left|F\left(\rho_{\epsilon }*\eta_{s}(y)\right)\right|}{\theta }\right),\eta_{s}(dy)\right\rangle {\left\langle \psi_{t-s}^{\epsilon ,x},\eta_{s}\right\rangle }^{k-j}ds\right|{ℱ}_{0}\right]\\ & \;\leq C^{'}\frac{\theta }{N\epsilon^{d}}\sum_{j=1}^{k-1}{\left\langle \psi_{t}^{\epsilon ,x}\left(y\right),\eta_{0}\left(dy\right)\right\rangle }^{j}\leq C^{''}\frac{\theta }{N\epsilon^{d}}\left({\left\langle \psi_{t}^{\epsilon ,x}\left(y\right),\eta_{0}\left(dy\right)\right\rangle }^{k}+\left\langle \psi_{t}^{\epsilon ,x}\left(y\right),\eta_{0}\left(dy\right)\right\rangle \right). \end{array}$$

Combining this with (7.11) and (7.12), using once again the fact that F is bounded above, we find

$$E\left[{\left\langle \psi_{0}^{\epsilon ,x},\eta_{t}\right\rangle }^{k}|{ℱ}_{0}\right]\leq {\left\langle \psi_{t}^{\epsilon ,x}(y),\eta_{0}(dy)\right\rangle }^{k}+\tilde{C}E\left[\int_{0}^{t}{\left\langle \psi_{t-s}^{\epsilon ,x}(y),\eta_{s}(dy)\right\rangle }^{k}ds|{ℱ}_{0}\right]\;\;+C^{''}\frac{\theta }{N\epsilon^{d}}\left({\left\langle \psi_{t}^{\epsilon ,x}(y),\eta_{0}(dy)\right\rangle }^{k}+\left\langle \psi_{t}^{\epsilon ,x}(y),\eta_{0}(dy)\right\rangle \right),$$

and (7.8) follows from Gronwall’s inequality. Rearranging exactly as in the case k=1, we recover (7.9) and the inductive step is complete. □

We shall also need the following consequence of the bounds that we obtained in Lemma 7.2:

#### Corollary 7.3

*Under the assumptions of* Theorem 2.20, *for each* k≥1,T>0, *there is a* C(k,T) *such that*

(7.13) $$E\left[\left\langle {\left(\rho_{\epsilon }*\eta_{t}\right)}^{k},\eta_{t}\right\rangle \right]<C\left(k,T\right)<\infty ,\;for\;all\;t\in \left[0,T\right].$$

#### Proof [Sketch]

First observe that if A∈(0,1), then

(7.14) $$p_{A\epsilon^{2}}(x,y)=\frac{1}{A^{d/2}}p_{\epsilon^{2}}(x,y)exp\left(-\frac{∥x-y{∥}^{2}}{2\epsilon^{2}}\left(\frac{1}{A}-1\right)\right)\leq \frac{1}{A^{d/2}}p_{\epsilon^{2}}(x,y).\;$$

Now consider

$$E\left[\left\langle \rho_{\epsilon }*\eta_{t}(x),\eta_{t}(dx)\right\rangle \right]\;=E\left[\int \int p_{\epsilon^{2}}(x,z)\eta_{t}(dz)\eta_{t}(dx)\right]\;\;=E\left[\int \int \int p_{\epsilon^{2}/2}(x,y)p_{\epsilon^{2}/2}(y,z)dy\eta_{t}(dz)\eta_{t}(dx)\right]\;\;=E\left[\int {\left(p_{\epsilon^{2}/2}*\eta_{t}(y)\right)}^{2}dy\right]\;\;\leq C\int E\left[{\left(\rho_{\epsilon }*\eta_{t}\left(x\right)\right)}^{2}\right]dx,$$

where we used (7.14) in the last line. Using Lemma 7.2 and our assumptions on $\eta_{0}$, this quantity is finite.

To illustrate the inductive step, now consider

(7.15) $$E\left[\left\langle \rho_{\epsilon }*\eta_{t}(x{)}^{2},\eta_{t}(dx)\right\rangle \right]=E\left[\int \int \int p_{\epsilon^{2}}\left(x,z_{1}\right)p_{\epsilon^{2}}\left(x,z_{2}\right)\eta_{t}\left(dz_{1}\right)\eta_{t}\left(dz_{2}\right)\eta_{t}(dx)\right]\;\;=E\left[\int \cdots \int p_{\epsilon^{2}/2}\left(x,y_{1}\right)p_{\epsilon^{2}/2}\left(x,y_{2}\right)p_{\epsilon^{2}/2}\left(y_{1},z_{1}\right)p_{\epsilon^{2}/2}\left(y_{2},z_{2}\right)\eta_{t}\left(dz_{1}\right)\eta_{t}\left(dz_{2}\right)dy_{1}dy_{2}\eta_{t}(dx)\right].$$

We use the identity

$$p_{\epsilon^{2}/2}\left(x,y_{1}\right)p_{\epsilon^{2}/2}\left(x,y_{2}\right)=p_{\epsilon^{2}}\left(y_{1},y_{2}\right)p_{\epsilon^{2}/4}\left(x,\frac{y_{1}+y_{2}}{2}\right)$$

to rewrite (7.15) as

$$E\left[\int \int p_{\epsilon^{2}/2}*\eta_{t}\left(y_{1}\right)p_{\epsilon^{2}/2}*\eta_{t}\left(y_{2}\right)p_{\epsilon^{2}/4}*\eta_{t}\left(\frac{y_{1}+y_{2}}{2}\right)p_{\epsilon^{2}}\left(y_{1},y_{2}\right)dy_{1}dy_{2}\right]\;\;\leq E\left[\int \int \left\{{\left(p_{\epsilon^{2}/2}*\eta_{t}\left(y_{1}\right)\right)}^{3}+{\left(p_{\epsilon^{2}/2}*\eta_{t}\left(y_{2}\right)\right)}^{3}+{\left(p_{\epsilon^{2}/4}*\eta_{t}\left(\frac{y_{1}+y_{2}}{2}\right)\right)}^{3}\right\}p_{\epsilon^{2}}\left(y_{1},y_{2}\right)dy_{1}dy_{2}\right],$$

where we have used that for any non-negative real numbers $\beta_{1},\;\beta_{2},\;\beta_{3},$ $\beta_{1}\beta_{2}\beta_{3}\leq \beta_{1}^{3}+\beta_{2}^{3}+\beta_{3}^{3}$. For the first two terms in the sum we integrate with respect to $y_{2}$ and $y_{1}$ respectively to reduce to an expression of the form considered in Lemma 7.2. For the final term, the change of variables $z_{1}=y_{1}+y_{2},\;z_{2}=y_{1}-y_{2}$ in the integral similarly allows us to integrate out the heat kernel, and we conclude that the result holds for k=2.

We can proceed in the same way for larger values of k, using repeatedly that

$$p_{t_{1}}\left(x,y_{1}\right)p_{t_{2}}\left(x,y_{2}\right)=p_{\frac{t_{1}t_{2}}{t_{1}+t_{2}}}\left(x,\frac{t_{2}y_{1}+t_{1}y_{2}}{t_{1}+t_{2}}\right)p_{t_{1}+t_{2}}\left(y_{1},y_{2}\right)$$

to write

$$\prod_{j=1}^{k}p_{\tau }\left(y,y_{j}\right)=\prod_{j=2}^{k}p_{\frac{j\tau }{j-1}}\left(y_{j},Y_{j-1}\right)p_{\frac{\tau }{k}}\left(y,Y_{k}\right)$$

where

$$Y_{1}=y_{1},\;Y_{j}=\frac{j-1}{j}Y_{j-1}+\frac{1}{j}y_{j},\;\text{for}\;j\geq 2.$$

Writing $p_{\epsilon^{2}}\left(x,z_{j}\right)=\int p_{\epsilon^{2}/2}\left(x,y_{j}\right)p_{\epsilon^{2}/2}\left(y_{j},z_{j}\right)dy_{j}$ and using the above with $\tau =\epsilon^{2}/2$, this yields

$$\langle {\left(\rho_{\epsilon }*\eta_{t}(x)\right)}^{k},\eta_{t}(dx)\rangle =\int \cdots \int \prod_{j=2}^{k}p_{\epsilon^{2}j/2(j-1)}\left(y_{j},Y_{j-1}\right)\prod_{i=1}^{k}p_{\epsilon^{2}/2}*\eta_{t}\left(y_{i}\right)p_{\epsilon^{2}/2k}*\eta_{t}\left(Y_{k}\right)dy_{1}\ldots dy_{k}\;\leq \;\int \cdots \int \prod_{j=2}^{k}p_{\epsilon^{2}j/2(j-1)}\left(y_{j},Y_{j-1}\right)\left\{\sum_{i=1}^{k}{\left(p_{\epsilon^{2}/2}*\eta_{t}\left(y_{i}\right)\right)}^{k+1}+{\left(p_{\epsilon^{2}/2k}*\eta_{t}\left(Y_{k}\right)\right)}^{k+1}\right\}dy_{1}\ldots dy_{k},$$

and once again we can change variables in the integrals and use (7.14) to bound this by a constant multiple of $\int E\left[{\left(\rho_{\epsilon }*\eta_{t}(x)\right)}^{k+1}\right]dx$, and the inductive step is complete. □

#### Corollary 7.4 (Tightness of $\left\{{\left(\rho_{\epsilon }*\eta_{t}^{N}(x)dx\right)}_{t\geq 0}\right\}$)

*Under the assumptions of Theorem 2.20, the sequence of measure valued processes* ${\left\{\rho_{\epsilon }*\eta_{t}^{N}(x)dx\right\}}_{t\geq 0}$ *(taking values in* ${𝒟}_{\left[0,T\right]}\left({ℳ}_{F}\left(R^{d}\right)\right)$*) is tight*.

#### Proof

First observe that the proof, from Lemma 6.2, that $E\left[{sup}_{0\leq t\leq T}\left\langle 1,\eta_{t}^{N}\right\rangle \right]$ is bounded goes through unchanged, and since $\left\langle 1,\rho_{\epsilon }*\eta_{t}^{N}(x)dx\right\rangle =\left\langle 1,\eta_{t}^{N}\right\rangle$, compact containment follows.

As in the nonlocal case, it suffices to prove that for T>0, and any $f\in C_{b}^{\infty }\left(R^{d}\right)$ with bounded second derivatives and ∫|f(x)|dx<∞, the sequence of real-valued processes ${\left\{{\left(\int f(x)\rho_{\epsilon }*\eta_{t}^{N}(x)dx\right)}_{t\geq 0}\right\}}_{N\geq 1}$ is tight. Let us temporarily write $X_{f}^{N}(t)$ for $\int f(x)\rho_{\epsilon }*\eta_{t}^{N}(x)dx$ and set

$$w^{'}\left(X_{f}^{N},\delta ,T\right)=\underset{\left\{t_{i}\right\}}{inf}\;\underset{i}{max}\;\underset{s,t\in \left[t_{i-1},t_{i}\right)}{sup}\;\left|X_{f}^{N}(t)-X_{f}^{N}(s)\right|,$$

where $\left\{t_{i}\right\}$ ranges over all partitions of the form $0=t_{0}<t_{1}<\cdots <t_{n-1}<T\leq t_{n}$ with ${min}_{1\leq i\leq n}\left(t_{i}-t_{i-1}\right)>\delta$ and n≥1. Using Corollary 3.7.4 of Ethier and Kurtz [1986], to prove tightness of the sequence of real-valued processes $X_{f}^{N}$ it suffices to check compact containment of the sequence ${\left\{\int f(x)\rho_{\epsilon }*\eta_{t}^{N}(x)dx\right\}}_{N\geq 1}$ at any rational time t and that for every ν>0 and T>0, there exists δ>0 such that

$$\underset{N\to \infty }{lim\;sup}\;P\left[w^{'}\left(X_{f}^{N},\delta ,T\right)>\nu \right]<\nu .$$

Evidently this will follow if we can show that this condition is satisfied when we replace the minimum over all partitions with mesh at least δ in the definition of $w^{'}$, by the partition into intervals of length exactly δ.

We have

(7.16) $$\left|\left\langle \rho_{\epsilon }*f,\eta_{t}^{N}\right\rangle -\left\langle \rho_{\epsilon }*f,\eta_{s}^{N}\right\rangle \right|\leq \left|\int_{s}^{t}\left\langle \theta \int \left(\rho_{\epsilon }*f(y)-\rho_{\epsilon }*f(x)\right)q_{\theta }(x,dy),\eta_{u}^{N}(dx)\right\rangle du\right|\;\;+\int_{s}^{t}\;\left\langle \left|F\left(\rho_{\epsilon }*\eta_{u}^{N}(x)\right)\right|\rho_{\epsilon }*|f|(x),\eta_{u}^{N}(dx)\right\rangle du+2\underset{0\leq u\leq T}{sup}\;\left|{\hat{M}}^{N}(f{)}_{u}\right|,$$

where ${\hat{M}}^{N}(f)$ is the martingale of (6.4) with the test function f replaced by $\rho_{\epsilon }*f$. We control each of the three terms on the right hand side separately.

By the Intermediate Value Theorem, using $T_{t}$ to denote the heat semigroup, there exists s∈(0,1/θ) such that

$$\left|\theta \int \left(\rho_{\epsilon }*f(y)-\rho_{\epsilon }*f(x)\right)q_{\theta }(x,dy)\right|=\left|\theta \left(T_{\epsilon^{2}+1/\theta }f(x)-T_{\epsilon^{2}}f(x)\right)\right|\;\;=\left|\partial_{s}T_{\epsilon^{2}+s}f(x)\right|=\left|T_{\epsilon^{2}+s}\Delta f(x)\right|\leq ∥\Delta f{∥}_{\infty }.$$

The first term in (7.16) is therefore bounded by

$$∥\Delta f{∥}_{\infty }|t-s|\underset{0\leq u\leq T}{sup}\;\left\langle 1,\eta_{u}^{N}\right\rangle .$$

We follow the approach of Lemma 6.2. Consulting (2.4), the angle bracket process of ${\hat{M}}^{N}(f)$ satisfies $E[{\left\langle {\hat{M}}_{f}^{N}\right\rangle }_{T}]\leq C(\theta /N)\int_{0}^{T}E\left[\left\langle 1,\eta_{s}\right\rangle \right]ds\leq C^{'}\theta /N$ for some constants C and $C^{'}$. Now, using the Burkholder-Davis-Gundy inequality and Barlow et al. [1986], $E[{sup}_{0\leq u\leq T}{\left|{\hat{M}}^{N}(f{)}_{u}\right|}^{2}]\leq C^{''}E[{\left\langle {\hat{M}}^{N}(f)\right\rangle }_{u}]$, and so using Markov’s inequality,

(7.17) $$\underset{N\to \infty }{lim\;sup}\;P\left[2\underset{0\leq u\leq T}{sup}\;|{\hat{M}}^{N}(f{)}_{u}|>\frac{\nu }{3}\right]\leq \underset{N\to \infty }{lim\;sup}\;\frac{36}{\nu^{2}}C^{''}E\left[{\left\langle {\hat{M}}^{N}\left(f\right)\right\rangle }_{T}\right]\leq \underset{N\to \infty }{lim\;sup}\;\frac{36}{\nu^{2}}\frac{C^{'}C^{''}\theta }{N\epsilon^{d}}=0.$$

Now consider

(7.18) $$E\left[{\left(\int_{s}^{t}\left\langle \rho_{\epsilon }*|f|(x)\left|F\left(\rho_{\epsilon }*\eta_{u}^{N}(x)\right)\right|,\eta_{u}^{N}(dx)\right\rangle du\right)}^{2}\right]\;=2E\left[\int_{s}^{t}\left\langle \rho_{\epsilon }*|f|(x)\left|F\left(\rho_{\epsilon }*\eta_{u}^{N}(x)\right)\right|,\eta_{u}^{N}(dx)\right\rangle \int_{u}^{t}\left\langle \rho_{\epsilon }*|f|(x)\left|F\left(\rho_{\epsilon }*\eta_{r}^{N}(x)\right)\right|,\eta_{r}^{N}(dx)\right\rangle drdu\right].$$

Since F is polynomial, we use the approach of Corollary 7.3, the tower property, and Lemma 7.2, to bound this in terms of sums of terms of the form

$$E\left[\int_{s}^{t}\;(t-u)\int \rho_{\epsilon }*|f|(x)\rho_{\epsilon }*\eta_{u}^{N}(x{)}^{j}dx\int \rho_{\epsilon }*|f|(y)\rho_{\epsilon }*\eta_{u}^{N}(y{)}^{k}dydu\right].$$

Now observe that, again using Lemma 7.2, since for nonnegative a and $b,\;a^{j}b^{k}\leq a^{j+k}+b^{j+k}$,

$$E\left[\int \int \rho_{\epsilon }*|f|(x)\rho_{\epsilon }*\eta_{u}^{N}(x{)}^{j}\rho_{\epsilon }*|f|(y)\rho_{\epsilon }*\eta_{u}^{N}(y{)}^{k}dxdy\right]\;\;\leq E\left[\int \int ∥f{∥}_{\infty }\rho_{\epsilon }*\eta_{u}^{N}(x{)}^{j+k}\rho_{\epsilon }*|f|(y)dxdy+\int \int \rho_{\epsilon }*|f|(x)∥f{∥}_{\infty }\rho_{\epsilon }*\eta_{u}^{N}(y{)}^{j+k}dxdy\right]\;\;\leq C\int |f|(x)dx.$$

Thus the quantity (7.18) is bounded by $C(t-s{)}^{2}$ for a new constant C which we can take to be independent of s,t and ϵ. Markov’s inequality then gives

$$P\left[∥f{∥}_{\infty }\int_{s}^{t}\left\langle \left|F\left(\rho_{\epsilon }*\eta_{u}^{N}(x)\right)\right|,\eta_{u}^{N}(dx)\right\rangle du\geq \frac{\nu }{3}\right]\leq C\frac{(t-s{)}^{2}}{\nu^{2}}.$$

A union bound gives that

(7.19) $$P\left[\underset{i}{max}∥f{∥}_{\infty }\int_{t_{i-1}}^{t_{i}}\left\langle \left|F\left(\rho_{\epsilon }*\eta_{u}^{N}(x)\right)\right|,\eta_{u}^{N}(dx)\right\rangle du\geq \frac{\nu }{3}\right]\leq C\frac{T\delta }{\nu^{2}}.$$

Now using Markov’s inequality, we can choose K so that

$$P\left[∥\Delta f{∥}_{\infty }\underset{0\leq t\leq T}{sup}\;\left\langle 1,\eta_{t}^{N}\right\rangle >K\right]<\frac{\nu }{3},$$

and so choosing δ so that Kδ<ν/3 in this expression and $C\delta <\nu^{3}/3T$ in (7.19), combining with (7.17), the result follows. □

### 7.2 Continuity estimates for $\rho_{\epsilon }*\eta$

To identify the limit point of any convergent subsequence of $\left\{\rho_{\epsilon }*\eta^{N}(x)\right\}$, we will require some control on the spatial continuity of the functions $\rho_{\epsilon }*\eta^{N}(x)$. This will be inherited from the regularity of the transition density of the Gaussian random walk with generator ${ℒ}^{\theta }$, which in turn follows from its representation as that of a Brownian motion evaluated at the random time T(t) defined in Lemma 7.1. Our approach will be to approximate $\psi_{t}^{\epsilon ,x}(\cdot )$ by $p_{\epsilon^{2}+t}(x,\cdot )$, and to control the error that this introduces we need to control T(t)-t.

#### Lemma 7.5

In the notation of Lemma 7.1, for any A>1,

$$P\left\{T(t)-t>A\left(\epsilon^{2}+t\right)\right\}\leq exp\left(-\frac{\theta A}{4}\left(\epsilon^{2}+t\right)\right).$$

#### Proof

This is just a Chernoff bound. With Π a rate one Poisson process as in Lemma 7.1, for any A>1,

$$P\left\{T(t)-t>A\left(\epsilon^{2}+t\right)\right\}\;=P\left\{\Pi (\theta t)>\theta \left(t+A\left(\epsilon^{2}+t\right)\right)\right\}\;\;\leq \frac{E[exp(\alpha \Pi (\theta t))]}{exp\left(\alpha \theta \left(t+A\left(\epsilon^{2}+t\right)\right)\right)}\;\;=exp\left(\theta t\left(e^{\alpha }-1\right)-\alpha \theta \left(t+A\left(\epsilon^{2}+t\right)\right)\right)\;\;\leq exp\left(\theta t\left(e^{\alpha }-\alpha -1-\frac{A\alpha }{2}\right)-\frac{A\alpha }{2}\theta \left(\epsilon^{2}+t\right)\right).$$

Now set α=1/2. Since $A>1,\;e^{\alpha }-\alpha -1-A\alpha /2<0$ and the result follows. □

As advertised, we wish to control the difference between $\psi_{t}^{\epsilon ,x}(y)$ and $p_{\epsilon^{2}+t}(x,y)$.

#### Lemma 7.6

In the notation of Lemma 7.1, there exists a C<∞ such that

(7.20) $$\left|\psi_{t}^{\epsilon ,x}(y)-p_{\epsilon^{2}+t}(x,y)\right|\leq \frac{C}{{\left(\epsilon^{2}\theta \right)}^{1/2}}p_{6\left(\epsilon^{2}+t\right)}(x,y)+\frac{C}{{\left(\epsilon^{2}+t\right)}^{d/2}}exp\left(-\epsilon^{2}\theta /2\right).$$

#### Proof

Still using the notation of Lemma 7.1, we partition into three events according to the value of τ(t). Let $A_{1}=\left\{\tau (t)<-\left(\epsilon^{2}+t\right)/2\right\},\;A_{2}=\left\{\tau (t)>2\left(\epsilon^{2}+t\right)\right\}$, and $A_{3}$ the remaining event, $\left\{-\left(\epsilon^{2}+t\right)/2\leq \tau (t)\leq 2\left(\epsilon^{2}+t\right)\right\}$. Then,

$$\left|\psi_{t}^{\epsilon ,x}(y)-p_{\epsilon^{2}+t}(x,y)\right|\;=\left|E\left[p_{\epsilon^{2}+t+\tau (t)}(x,y)-p_{\epsilon^{2}+t}(x,y)\right]\right|\;\;\leq E\left[\left(1_{A_{1}}+1_{A_{2}}+1_{A_{3}}\right)\left|p_{\epsilon^{2}+t+\tau \left(t\right)}\left(x,y\right)-p_{\epsilon^{2}+t}\left(x,y\right)\right|\right].$$

For the first term, note that if a<b then

$$\left|p_{a}(x,y)-p_{b}(x,y)\right|\;=\frac{1}{(2\pi {)}^{d/2}}\left|\frac{1}{a^{d/2}}e^{-∥x-y{∥}^{2}/2a}-\frac{1}{b^{d/2}}e^{-∥x-y{∥}^{2}/2b}\right|\;\;=\frac{1}{{\left(2\pi a^{2}\right)}^{d/2}}e^{-∥x-y{∥}^{2}/2b}\left|e^{-∥x-y{∥}^{2}\left(\frac{1}{2a}-\frac{1}{2b}\right)}-{\left(\frac{a}{b}\right)}^{d/2}\right|\;\;\leq C{\left(\frac{b}{a}\right)}^{d/2}p_{b}(x,y),$$

where the inequality follows because both terms under the absolute value are less than 1. Since, on the event $A_{1},\;\tau (t)<0$, we can apply this with $a=\epsilon^{2}+t+\tau (t)$ and $b=\epsilon^{2}+t$, and, using the bound (7.6),

$$E\left[1_{A_{1}}\left|p_{a}(x,y)-p_{b}(x,y)\right|\right]\;\leq C{\left(\frac{\epsilon^{2}+t}{\epsilon^{2}}\right)}^{d/2}p_{\epsilon^{2}+t}(x,y)P\left\{\tau (t)<-\frac{\epsilon^{2}+t}{2}\right\}\;\;\leq C\frac{1}{\epsilon^{d}}P\left\{\tau (t)<-\frac{\epsilon^{2}+t}{2}\right\}\;\;\leq \frac{C}{{\left(\epsilon^{2}+t\right)}^{d/2}}exp\left(-\frac{\theta \epsilon^{2}}{2}\right).$$

For the third term, we will first collect some facts. Observe that on the event $A_{3},\;\epsilon^{2}+t+\tau (t)$ is between $\left(\epsilon^{2}+t\right)/2$ and $3\left(\epsilon^{2}+t\right)$, and for any s in this interval,

(7.21) $$p_{2s}(y)\;\leq {\left(\frac{6\left(\epsilon^{2}+t\right)}{\epsilon^{2}+t}\right)}^{d/2}p_{6\left(\epsilon^{2}+t\right)}(x,y)\;\;=6^{d/2}p_{6\left(\epsilon^{2}+t\right)}(x,y).$$

Moreover, since $ue^{-u}\leq e^{-1}$ for all u≥0,

(7.22) $$\frac{∥x-y{∥}^{2}}{s}p_{s}(x,y)\;=\frac{4}{(2\pi s{)}^{d/2}}e^{-\frac{∥x-y{∥}^{2}}{4s}}\frac{∥x-y{∥}^{2}}{4s}e^{-\frac{∥x-y{∥}^{2}}{4s}}\;\;\leq Cp_{2s}(x,y).$$

Now, by the Intermediate Value Theorem,

(7.23) $$\left|p_{\epsilon^{2}+t+\tau (t)}(x,y)-p_{\epsilon^{2}+t}(x,y)\right|=|\tau (t)|\left|\frac{\partial p_{s}(x,y)}{\partial s}\right|$$

for some s between $\epsilon^{2}+t+\tau (t)$ and $\epsilon^{2}+t$. Since

$$\partial_{s}p_{s}(x,y)\;=\partial_{s}\left(\frac{1}{(2\pi s{)}^{d/2}}exp\left(-\frac{∥x-y{∥}^{2}}{2s}\right)\right)\;\;=-\frac{d}{2s}p_{s}(x,y)+\frac{∥x-y{∥}^{2}}{2s^{2}}p_{s}(x,y),$$

applying the inequality (7.22), using the fact that $p_{s}(x,y)\leq 2^{d/2}p_{2s}(x,y)$, and then (7.21), we have that for any $s\in \left(\left(\epsilon^{2}+t\right)/2,3\left(\epsilon^{2}+t\right)\right)$,

$$\left|\frac{\partial }{\partial s}p_{s}(x,y)\right|\leq \frac{C}{s}p_{2s}\left(x,y\right)\leq \frac{C}{\epsilon^{2}+t}p_{6\left(\epsilon^{2}+t\right)}\left(x,y\right).$$

Therefore, recalling that $E[\tau (t{)}^{2}]=t/\theta$, substituting into (7.23),

$$E\left[1_{A_{3}}\left|p_{\epsilon^{2}+t+\tau (t)}(x,y)-p_{\epsilon^{2}+t}(x,y)\right|\right]\;\leq \frac{C}{\epsilon^{2}+t}p_{6\left(\epsilon^{2}+t\right)}(x,y)E[|\tau (t)|]\;\;\leq \frac{C}{\epsilon^{2}+t}p_{6\left(\epsilon^{2}+t\right)}(x,y)E{\left[\tau (t{)}^{2}\right]}^{1/2}\;\;={\left(\frac{Ct}{\theta {\left(\epsilon^{2}+t\right)}^{2}}\right)}^{1/2}p_{6\left(\epsilon^{2}+t\right)}(x,y)\;\;\leq \frac{C}{\sqrt{\theta \epsilon^{2}}}p_{6\left(\epsilon^{2}+t\right)}\left(x,y\right),$$

where the last inequality follows from $2\epsilon^{2}t\leq {\left(\epsilon^{2}+t\right)}^{2}$.

Finally, on the event $A_{2}=\left\{\tau (t)>2\left(\epsilon^{2}+t\right)\right\}$, we simply use

$$\left|p_{\epsilon^{2}+t+\tau (t)}(x,y)-p_{\epsilon^{2}+t}(x,y)\right|\leq \frac{C}{{\left(\epsilon^{2}+t\right)}^{d/2}},$$

so that

$$E\left[1_{A_{2}}\left|p_{\epsilon^{2}+t+\tau (t)}(x,y)-p_{\epsilon^{2}+t}(x,y)\right|\right]\leq \frac{C}{{\left(\epsilon^{2}+t\right)}^{d/2}}P\left\{\tau (t)>2\left(\epsilon^{2}+t\right)\right\},$$

and apply Lemma 7.5 with A=2. □

The last result will be useful when combined with the next bound for the heat kernel.

#### Lemma 7.7

*Let* s>0, and $x,\;y,\;z\in R^{d}$. *The following estimate holds:*

$$\left|p_{s}(x,z)-p_{s}(y,z)\right|\leq \frac{C∥x-y∥}{\sqrt{s}}\left(p_{2s}\left(x,z\right)+p_{2s}\left(y,z\right)\right),$$

where the constant C does not depend on x,y,z or s.

#### Proof

Expanding the difference of two squares,

$$e^{-\frac{∥y-z{∥}^{2}}{2s}}-e^{-\frac{∥x-z{∥}^{2}}{2s}}=\left(e^{-\frac{∥y-z{∥}^{2}}{4s}}-e^{-\frac{∥x-z{∥}^{2}}{4s}}\right)\left(e^{-\frac{∥y-z{∥}^{2}}{4s}}+e^{-\frac{∥x-z{∥}^{2}}{4s}}\right).$$

Now, thinking of the first term in brackets as a function of a single variable x on the line segment [y,z] connecting y to z, we can apply the Intermediate Value Theorem and take the modulus to bound this expression by

$$∥y-x∥\left(\frac{2∥w-z∥}{4s}exp\left(-\frac{∥w-z{∥}^{2}}{4s}\right)\right)(4\pi s{)}^{d/2}\left(p_{2s}(y,z)+p_{2s}(x,z)\right)$$

for some w∈[y,z]. Using the fact that $xe^{-x^{2}}$ is uniformly bounded, we can bound the first bracket in the last equation by $C/\sqrt{s}$, and the result follows. □

We now have the ingredients that we need to write down a continuity estimate for $\rho_{\epsilon }*\eta$. We fix δ>0 and suppose that s>δ. Let us write

$$\hat{\epsilon }\left(\delta ,\epsilon ,\theta \right):=\frac{1}{{\left(\epsilon^{2}+\delta \right)}^{d/2}}e^{-\epsilon^{2}\theta /2},$$

and note that under the assumption that $\epsilon^{2}\theta \to \infty$, for each fixed $\delta >0,\;{lim}_{\epsilon \to 0,\theta \to \infty }\;\hat{\epsilon }(\delta ,\epsilon ,\theta )=0$. Using the semimartingale decomposition (7.4), and Lemma 7.6, we have

(7.24) $$\left|\rho_{\epsilon }*\eta_{s}(y)-\rho_{\epsilon }*\eta_{s}(w)\right|=\left|\left\langle p_{\epsilon^{2}}(y,z)-p_{\epsilon^{2}}(w,z),\eta_{s}(dz)\right\rangle \right|\;\;\leq \left\langle \left|p_{\epsilon^{2}+s}(y,z)-p_{\epsilon^{2}+s}(w,z)\right|,\eta_{0}(dz)\right\rangle \;\;+\int_{0}^{s-\delta }\left\langle |p_{s-r+\epsilon^{2}}(y,z)-p_{s-r+\epsilon^{2}}(w,z)|||F\left(\rho_{\epsilon }*\eta_{r}(z)\left|,\eta_{r}(dz)\right\rangle dr\right.\right.\;\;+\langle \frac{C}{{\left(\theta \epsilon^{2}\right)}^{1/2}}\left(p_{6\left(\epsilon^{2}+s\right)}(y,z)+p_{6\left(\epsilon^{2}+s\right)}(w,z)\right)+C\hat{\epsilon }(\delta ,\epsilon ,\theta ),\eta_{0}(dz)\rangle \;\;+\int_{0}^{s-\delta }\langle \left\{\frac{C}{{\left(\epsilon^{2}\theta \right)}^{1/2}}\left|p_{6\left(s-r+\epsilon^{2}\right)}(y,z)+p_{6\left(s-r+\epsilon^{2}\right)}(w,z)\right|+\hat{\epsilon }(\delta ,\epsilon ,\theta )\right\}\rangle F\left(\rho_{\epsilon }*\eta_{r}(z)\left|,\eta_{r}(dz)\right\rangle dr\right.\;\;+\int_{s-\delta }^{s}\left\langle \left|\psi_{s-r}^{\epsilon ,y}(z)+\psi_{s-r}^{\epsilon ,w}(z)\right|\right|F\left(\rho_{\epsilon }*\eta_{r}(z)\left|,\eta_{r}(dz)\right\rangle dr\right.\;\;+\left|M_{s}(y)\right|+\left|M_{s}(w)\right|\;\;\leq \langle \frac{∥y-w∥}{\sqrt{s+\epsilon^{2}}}\left(p_{2\left(s+\epsilon^{2}\right)}(y,z)+p_{2\left(s+\epsilon^{2}\right)}(w,z)\right),\eta_{0}(dz)\rangle \;\;+\int_{0}^{s-\delta }\left\langle \frac{∥y-w∥}{\sqrt{s-r+\epsilon^{2}}}\left(p_{2\left(s-t+\epsilon^{2}\right)}(y,z)+p_{2\left(s-r+\epsilon^{2}\right)}(w,z)\right)\right|F\left(\rho_{\epsilon }*\eta_{r}(z)\left|,\eta_{r}(dz)\right\rangle dr\right.\;\;+\langle \frac{C}{{\left(\theta \epsilon^{2}\right)}^{1/2}}\left(p_{6\left(\epsilon^{2}+s\right)}(y,z)+p_{6\left(\epsilon^{2}+s\right)}(w,z)\right)+C\hat{\epsilon }(\delta ,\epsilon ,\theta ),\eta_{0}(dz)\rangle \;\;+\int_{0}^{s-\delta }\left\langle \left\{\frac{C}{{\left(\epsilon^{2}\theta \right)}^{1/2}}\left(p_{6\left(s-r+\epsilon^{2}\right)}(y,z)+p_{6\left(s-r+\epsilon^{2}\right)}(w,z)\right)+\hat{\epsilon }(\delta ,\epsilon ,\theta )\right\}\right|F\left(\rho_{\epsilon }*\eta_{r}(z)\left|,\eta_{r}(dz)\right\rangle dr\right.\;\;+\int_{s-\delta }^{s}\left\langle \left|\psi_{s-r}^{\epsilon ,y}(z)+\psi_{s-r}^{\epsilon ,w}(z)\right|\right|F\left(\rho_{\epsilon }*\eta_{r}(z)\left|,\eta_{r}(dz)\right\rangle dr\right.\;\;+\left|M_{s}(y)\right|+\left|M_{s}(w)\right|.$$

Although this expression is lengthy, we have successfully isolated the terms involving ∥y-w∥, which will control the regularity as we pass to the limit. Asymptotically, we don’t expect the martingale terms to contribute, since their quadratic variation scales with $\theta /\left(N\epsilon^{d}\right)$; under the assumption that $\epsilon^{2}\theta \to \infty$, for any fixed δ>0, the terms arising from approximating the transition density $\psi_{s-r}^{\epsilon ,\cdot }(\cdot )$ of the Gaussian walk by $p_{s-r+\epsilon^{2}}(\cdot ,\cdot )$ at times with s-r>δ will tend to zero; and the moment bounds of Lemma 7.2 will allow us to control the integral over [s-δ,s]. There is some technical work to be done to rigorously identify the limit points of $\rho_{\epsilon }*\eta^{N}$, but it really amounts to applying the tower property and our moment bounds from Lemma 7.2 and Corollary 7.3.

### 7.3 Identification of the limit

We now turn to the identification of the limit points of the sequence ${\left\{{\left(\rho_{\epsilon }*\eta_{t}^{N}(x)dx\right)}_{t\geq 0}\right\}}_{N\geq 1}$. We would like to show that any limit point solves (2.16) in the limit, i.e.,

(7.25) $$\left\langle f\left(x\right),\varphi \left(t,x\right)dx\right\rangle =\int_{0}^{t}\left\langle \frac{1}{2}\Delta f\left(x\right)+f\left(x\right)F\left(\varphi \left(s,x\right)\right),\varphi \left(s,x\right)dx\right\rangle ds.$$

Since $\left\langle f,\rho_{\epsilon }*\eta_{t}^{N}(x)dx\right\rangle =\left\langle \rho_{\epsilon }*f(x),\eta_{t}^{N}(dx)\right\rangle$, and the limit is deterministic, this will follow if we can show that each of the terms in the semimartingale decomposition (2.3), with the test function f replaced by $\rho_{\epsilon }*f(\cdot )$, converges to the corresponding term in (7.25).

The linear term is straightforward. Write T. for the heat semigroup, so that $\rho_{\epsilon }*f(x)=T_{\epsilon^{2}}f(x)$. By a Taylor expansion,

$$\int_{0}^{t}\langle {ℒ}^{\theta }T_{\epsilon^{2}}f,\eta_{s}^{N}(dx)\rangle ds\;=\int_{0}^{t}\left\langle \frac{1}{2}\Delta T_{\epsilon^{2}}f(x),\eta_{s}^{N}(dx)\right\rangle +𝒪\left(\frac{1}{\theta }\right)\;\;=\int_{0}^{t}\left\langle \frac{1}{2}\Delta f(x),\rho_{\epsilon }*\eta_{s}^{N}(x)dx\right\rangle ds+𝒪\left(\frac{1}{\theta }\right).$$

Thus, from weak convergence we can deduce that under our scaling, for any (weakly) convergent subsequence ${\left\{\rho_{\epsilon }*\eta^{N}(x)dx\right\}}_{N\geq 1}$,

$$\int_{0}^{t}\langle {ℒ}^{\theta }T_{\epsilon^{2}}f,\eta_{s}^{N}(dx)\rangle ds\to \int_{0}^{t}\left\langle \frac{1}{2}\Delta f(x),\varphi (s,x)dx\right\rangle ds.$$

The nonlinear term in the semimartingale decomposition is more intricate. It takes the form

$$E\left[\int_{0}^{t}\left\langle T_{\epsilon^{2}}f(y)F\left(\rho_{\epsilon }*\eta_{s}(y)\right),\eta_{s}(dy)\right\rangle ds\right]$$

and we should like to show that this converges to

$$\int_{0}^{t}\int f\left(y\right)F\left(\varphi \left(s,y\right)\right)\varphi \left(s,y\right)dyds.$$

We proceed in stages. First we should like to transfer the heat semigroup from $T_{\epsilon^{2}}f$ onto $\eta_{s}$. Since f is smooth, this will follow easily if we can show that

$$E\left[\int_{0}^{t}\left\langle T_{\epsilon^{2}}f(y)F\left(\rho_{\epsilon }*\eta_{s}(y)\right),\eta_{s}(dy)\right\rangle ds\right]∼E\left[\int_{0}^{t}\left\langle T_{\epsilon^{2}}f\left(y\right)F\left(\rho_{\epsilon }*\eta_{s}\left(y\right)\right),\rho_{\epsilon }*\eta_{s}\left(y\right)dy\right\rangle ds\right].$$

This is the content of Proposition 7.8.

#### Proposition 7.8

Under the conditions of Theorem 2.20,

(7.26) $$\underset{\epsilon \to 0}{lim}\;E\left[\left|\int_{0}^{t}\left\langle T_{\epsilon^{2}}f(y)F\left(\rho_{\epsilon }*\eta_{s}(y)\right),\eta_{s}(y)\right\rangle -\left\langle T_{\epsilon^{2}}f(y)F\left(\rho_{\epsilon }*\eta_{s}(y)\right),\rho_{\epsilon }*\eta_{s}(y)dy\right\rangle ds\right|\right]=0.$$

#### Proof

In fact we are going to fix δ>0, with t>δ, and show that the expression on the left hand side of (7.26) is less than a constant times δ, with a constant independent of δ,N, and ϵ. Since δ is arbitrary, the result will follow.

We first note that,

$$\left\langle T_{\epsilon^{2}}f(y)F\left(\rho_{\epsilon }*\eta_{s}(y)\right),\rho_{\epsilon }*\eta_{s}(y)dy\right\rangle -\left\langle T_{\epsilon^{2}}f(y)F\left(\rho_{\epsilon }*\eta_{s}(dy)\right),\eta_{s}(dy)\right\rangle \;\;=\left\langle \int T_{\epsilon^{2}}f(y)F\left(\rho_{\epsilon }*\eta_{s}(y)\right)\rho_{\epsilon }(y-w)dy,\eta_{s}(dw)\right\rangle -\left\langle T_{\epsilon^{2}}f(w)F\left(\rho_{\epsilon }*\eta_{s}(w)\right),\eta_{s}(dw)\right\rangle \;\;=\left\langle \int \left\{T_{\epsilon^{2}}f(y)F\left(\rho_{\epsilon }*\eta_{s}(y)\right)-T_{\epsilon^{2}}f(w)F\left(\rho_{\epsilon }*\eta_{s}(w)\right)\right\}\rho_{\epsilon }(w-y)dy,\eta_{s}(dw)\right\rangle .$$

Let us denote the integral against dy in the last expression by I, that is

$$I:=\int \left\{T_{\epsilon^{2}}f\left(y\right)F\left(\rho_{\epsilon }*\eta_{s}\left(y\right)\right)-T_{\epsilon^{2}}f\left(w\right)F\left(\rho_{\epsilon }*\eta_{s}\left(w\right)\right)\right\}\rho_{\epsilon }\left(w-y\right)dy,$$

and note that |I| is bounded by

(7.27) $$\left.\int \left\{\left|F\left(\rho_{\epsilon }*\eta_{s}(y)\right)-F\left(\rho_{\epsilon }*\eta_{s}(w)\right)\right|T_{\epsilon^{2}}f(y)+F\left(\rho_{\epsilon }*\eta_{s}(w)\right)|T_{\epsilon^{2}}f(y)-T_{\epsilon^{2}}f(w)\right)|\right\}\rho_{\epsilon }(w-y)dy\;\;\leq \int ∥f{∥}_{\infty }\left|F\left(\rho_{\epsilon }*\eta_{s}\left(y\right)\right)-F\left(\rho_{\epsilon }*\eta_{s}\left(w\right)\right)\right|\rho_{\epsilon }\left(w-y\right)dy+C\epsilon {∥f^{'}∥}_{\infty }\left|F\left(\rho_{\epsilon }*\eta_{s}\left(w\right)\right)\right|,$$

where we have used that

$$\int \left|T_{\epsilon^{2}}f\left(y\right)-T_{\epsilon^{2}}f\left(w\right)\right|p_{\epsilon^{2}}\left(w,y\right)dy\leq {∥f^{'}∥}_{\infty }\int \left|y-w\right|p_{\epsilon^{2}}\left(w,y\right)dy.$$

Now recall that F is a polynomial of degree n, and so there exist real numbers $b_{k}$ such that $F(a)-F(b)=(a-b)\sum_{k=1}^{n-1}\;b_{k}a^{k}b^{n-1-k}$ and so

$$\left|F\left(\rho_{\epsilon }*\eta_{s}(y)\right)-F\left(\rho_{\epsilon }*\eta_{s}(w)\right)\right|\leq \left|\rho_{\epsilon }*\eta_{s}(y)-\rho_{\epsilon }*\eta_{s}(w)\right|\sum_{k=1}^{n-1}\left|b_{k}\right|\left(\rho_{\epsilon }*\eta_{s}{\left(y\right)}^{n-1}+\rho_{\epsilon }*\eta_{s}{\left(w\right)}^{n-1}\right).$$

Combining the above, we have reduced the problem to showing that for any k≥0,

(7.28) $$\underset{\epsilon \to 0}{lim}\;E[\int_{0}^{t}\langle \int \left|\rho_{\epsilon }*\eta_{s}(y)-\rho_{\epsilon }*\eta_{s}(w)\right|{\left(\rho_{\epsilon }*\eta_{s}(y)\right)}^{k}+\rho_{\epsilon }*\eta_{s}(w{)}^{k}p_{\epsilon^{2}}(w,y)dy,\eta_{s}(dw)\rangle ds]=0.$$

We are going to use the estimate (7.24). First note that by Lemma 7.2 the contribution to (7.28) from the integral over the time interval [0,δ] is 𝒪(δ). We focus instead on the interval (δ,t].

The first term in (7.24) gives

$$\int_{\delta }^{t}\;E[\langle \int \langle \frac{∥y-w∥}{\sqrt{s+\epsilon^{2}}}\left(p_{2\left(s+\epsilon^{2}\right)}(y,z)+p_{2\left(s+\epsilon^{2}\right)}(w,z)\right),\eta_{0}(dz)\rangle \;\;\left.\;(\rho_{\epsilon }*\eta_{s}(y{)}^{k}+\rho_{\epsilon }*\eta_{s}(w{)}^{k})p_{\epsilon^{2}}(w,y)dy,\eta_{s}(dw)\right\rangle ]ds.$$

We “borrow” from the exponential term to see that $∥y-w∥p_{\epsilon^{2}}(w,y)\leq C\epsilon p_{2\epsilon^{2}}(w,y)$ and so bound this by

(7.29) $$C\int_{\delta }^{t}\;\frac{\epsilon }{\sqrt{s+\epsilon^{2}}}E\left[\left\langle \int \left\langle \left(p_{2\left(s+\epsilon^{2}\right)}(y,z)+p_{2\left(s+\epsilon^{2}\right)}(w,z)\right),\eta_{0}(dz)\right\rangle \right.\right.\;\;\left.\left.\;\left(\rho_{\epsilon }*\eta_{s}(y{)}^{k}+\rho_{\epsilon }*\eta_{s}(w{)}^{k}\right)p_{2\epsilon^{2}}(w,y)dy,\eta_{s}(dw)\right\rangle ds\right].$$

The four terms in the product are taken separately, according to the combinations of w and y appearing. First,

$$\int_{\delta }^{t}\frac{\epsilon }{\sqrt{s+\epsilon^{2}}}E\left[\left\langle \int \left\langle p_{2\left(s+\epsilon^{2}\right)}(y,z),\eta_{0}(dz)\right\rangle \rho_{\epsilon }*\eta_{s}(y{)}^{k}p_{2\epsilon^{2}}(w,y)dy,\eta_{s}(dw)\right\rangle \right]ds$$

can be rewritten as

$$\;\int_{\delta }^{t}\;\frac{\epsilon }{\sqrt{s+\epsilon^{2}}}E\left[\int \int \left\langle p_{2\left(s+\epsilon^{2}\right)}(y,z),\eta_{0}(dz)\right\rangle \rho_{\epsilon }*\eta_{s}(y{)}^{k}p_{\epsilon^{2}}(x,y)\rho_{\epsilon }*\eta_{s}(x)dydxds\right]\;\leq \;\int_{\delta }^{t}\;\frac{\epsilon }{\sqrt{s+\epsilon^{2}}}E\left[\int \int \left\langle p_{2\left(s+\epsilon^{2}\right)}(y,z),\eta_{0}(dz)\right\rangle \left(\rho_{\epsilon }*\eta_{s}(y{)}^{k+1}+\rho_{\epsilon }*\eta_{s}(x{)}^{k+1}\right)p_{\epsilon^{2}}(x,y)dydx\right]ds,$$

and using Lemma 7.2 and the tower property, and integrating with respect to s, under our assumptions on $\eta_{0}$, this is bounded by

$$C\epsilon \int_{\delta }^{t}\;\frac{1}{\sqrt{s+\epsilon^{2}}}E\left[\int \int \left\langle p_{2\left(s+\epsilon^{2}\right)}(y,z),\eta_{0}(dz)\right\rangle \right.\;\;\left.\;\left(\rho_{\epsilon }*\eta_{0}(y)+\rho_{\epsilon }*\eta_{0}(y{)}^{k+1}+\rho_{\epsilon }*\eta_{0}(x)+\rho_{\epsilon }*\eta_{0}(x{)}^{k+1}\right)p_{\epsilon^{2}}(x,y)dydx\right]ds\;\;\leq C^{'}\epsilon \int_{\delta }^{t}\;\frac{1}{{\left(s+\epsilon^{2}\right)}^{d/2}}ds.$$

For fixed δ, this bound tends to zero as ϵ→0. The term involving $\left\langle p_{2\left(s+\epsilon^{2}\right)}(w,z),\eta_{0}(dz)\right\rangle \rho_{\epsilon }*\eta_{s}(w{)}^{k}$ is handled similarly.

On the other hand

$$\left\langle \int \left\langle p_{2\left(s+\epsilon^{2}\right)}(y,z),\eta_{0}(dz)\right\rangle \rho_{\epsilon }*\eta_{s}(w{)}^{k}p_{2\epsilon^{2}}(w,y)dy,\eta_{s}(dw)\right\rangle \;\;\leq \frac{C}{{\left(s+\epsilon^{2}\right)}^{d/2}}\left\langle 1,\eta_{0}\right\rangle \left\langle \rho_{\epsilon }*\eta_{s}(w{)}^{k},\eta_{s}(dw)\right\rangle ,$$

and since $\left\langle 1,\eta_{0}\right\rangle$ is uniformly bounded we apply Corollary 7.3 to obtain a bound on the contribution to (7.29) from this term of the same form as the others.

Now consider the contribution to the left hand side of (7.28) from the second term in (7.24). Since F is a polynomial, it is bounded by a sum of terms of the form

$$\int_{\delta }^{t}\;\int_{0}^{s-\delta }\;\left\langle \int \frac{∥y-w∥}{\sqrt{s-r+\epsilon^{2}}}\left\langle \left(p_{2\left(s-r+\epsilon^{2}\right)}(y,z)+p_{2\left(s-r+\epsilon^{2}\right)}(w,z)\right)\rho_{\epsilon }*\eta_{r}(z{)}^{j},\eta_{r}(dz)\right\rangle \right.\;\;\left.\rho_{\epsilon }*\eta_{s}(y{)}^{k}p_{\epsilon^{2}}(y,w)dy,\eta_{s}(dw)\right\rangle drds\;\;\leq C\epsilon \int_{\delta }^{t}\;\int_{0}^{s-\delta }\;\frac{1}{\sqrt{s-r+\epsilon^{2}}}\left\langle \int \langle \left(p_{2\left(s-r+\epsilon^{2}\right)}(y,z)+p_{2\left(s-r+\epsilon^{2}\right)}(w,z)\right)\rho_{\epsilon }*\eta_{r}(z{)}^{j},\eta_{r}(dz)\rangle \right.\;\;\left.\rho_{\epsilon }*\eta_{s}(y{)}^{k}p_{2\epsilon^{2}}(y,w)dy,\eta_{s}(dw)\right\rangle drds,$$

where as usual we have “borrowed” from the exponential term in $p_{\epsilon }^{2}(y,w)$ to replace ∥y-w∥ by a constant times ϵ.

Once again, our approach is to rearrange terms so that we can apply Lemma 7.2 or Corollary 7.3 to obtain a bound on the contribution to (7.28) from these terms of the form Cϵ (where C may depend on δ but not ϵ).

For example, using the Chapman-Kolmogorov equation to rewrite

$$\int \left\langle \left\langle p_{2\left(s-r+\epsilon^{2}\right)}(y,z)\rho_{\epsilon }*\eta_{r}(z{)}^{j},\eta_{r}(dz)\rangle \rho_{\epsilon }*\eta_{s}(y{)}^{k}p_{2\epsilon^{2}}(y,w),\eta_{s}(dw\right)\right\rangle dy$$

as

$$\int \int \langle p_{2\left(s-r+\epsilon^{2}\right)}(y,z)\rho_{\epsilon }*\eta_{r}(z{)}^{j},\eta_{r}(dz)\rangle \rho_{\epsilon }*\eta_{s}(y{)}^{k}p_{\epsilon^{2}}\left(y,x\right)\rho_{\epsilon }*\eta_{s}\left(x\right)dxdy,$$

and using Lemma 7.2 and the tower property, we are led to control terms of the form

$$E\left[\int \left\langle p_{2\left(s-r+\epsilon^{2}\right)}(y,z)\rho_{\epsilon }*\eta_{r}(z{)}^{j},\eta_{r}(dz)\right\rangle \rho_{\epsilon }*\eta_{r}(y{)}^{k+1}dy\right].$$

This, in turn, is at most

$$E\left[\left\langle \rho_{\epsilon }*\eta_{r}(z{)}^{j+k+1},\eta_{r}(dz)\right\rangle \right]+E\left[\int \int p_{2(s-r)}(y,x)\rho_{\epsilon }*\eta_{r}(x)\rho_{\epsilon }*\eta_{r}(y{)}^{j+k+1}dydx\right]\;\;\leq E\left[\left\langle \rho_{\epsilon }*\eta_{r}(z{)}^{j+k+1},\eta_{r}(dz)\right\rangle \right]+2\int E\left[\rho_{\epsilon }*\eta_{r}(x{)}^{j+k+2}\right]dx,$$

which is bounded by Lemma 7.2.

We now turn to the contribution arising from the martingale terms in (7.24):

$$E\left[\int_{\delta }^{t}\;\left\langle \int \left(\left|M_{s}(y)\right|+\left|M_{s}(w)\right|\right)\left(\rho_{\epsilon }*\eta_{s}(y{)}^{k}+\rho_{\epsilon }*\eta_{s}(w{)}^{k}\right)p_{\epsilon^{2}}(w,y)dy,\eta_{s}(dw)\right\rangle ds\right].$$

Since $\psi_{t-s}^{\epsilon ,x}(y)=E\left[p_{T(t-s)+\epsilon^{2}}(x,y)\right]$, rearranging (7.4) we see that we can pull a convolution with $p_{\epsilon^{2}/2}$ out of our expressions for $M_{s}(y)$ and $M_{s}(w)$ and so all the manipulations that we used to control terms above with still be valid. To deal with the two terms in the product involving $\left|M_{s}(y)\right|$, we write the first as $\int \left|M_{s}(y)\right|\rho_{\epsilon }*\eta_{s}(y{)}^{k+1}dy$ and then use Hölder’s inequality, Lemma 7.2, and the fact that $E\left[{\left|M_{s}(y)\right|}^{2}\right]$ is $𝒪\left(\theta /\left(N\epsilon^{d}\right)\right)$ to see that the contribution from this term tends to zero in the limit. For the second, we use the idea of the proof of Corollary 7.3 to reduce to a form to which we can apply Hölder’s inequality.

Control of the terms arising from approximating $\psi^{\epsilon ,x}$ by the heat kernel follows in an entirely analogous way.

Combining the above, we see that given δ>0,

$$\underset{\epsilon \to 0}{lim}\;E\left[\left|\int_{0}^{t}\left\langle T_{\epsilon^{2}}f(y)F\left(\rho_{\epsilon }*\eta_{s}(y)\right),\eta_{s}(y)\right\rangle ds-\int_{0}^{t}\left\langle T_{\epsilon^{2}}f(y)F\left(\rho_{\epsilon }*\eta_{s}(y)\right),\rho_{\epsilon }*\eta_{s}(y)dy\right\rangle ds\right|\right]<C\delta ,$$

where the constant C is independent of δ. Since δ was arbitrary, the proof is complete. □

Since f is smooth, $T_{\epsilon }^{2}f-f$ is 𝒪(ϵ), with an application of the triangle inquality,

$$\underset{\epsilon \to 0}{lim}\;E\left[\left|\int_{0}^{t}\left\langle T_{\epsilon^{2}}f(y)F\left(\rho_{\epsilon }*\eta_{s}(y)\right),\eta_{s}(y)\right\rangle ds-\int_{0}^{t}\left\langle f(y)F\left(\rho_{\epsilon }*\eta_{s}(y)\right),\rho_{\epsilon }*\eta_{s}(y)dy\right\rangle ds\right|\right]<C\delta ,$$

now follows immediately. Thus to complete the characterisation of the limit, it remains to show that if we take a convergent subsequence $\left\{{\left(\rho_{\epsilon }*\eta_{t}^{N}(dx)\right)}_{t\geq 0}\right\}$ converging to a limit point $(\varphi (t,x)dx{)}_{t\geq 0}$, then

$$\int_{0}^{t}\int f\left(x\right)\rho_{\epsilon }*\eta_{s}\left(x\right)F\left(\rho_{\epsilon }*\eta_{s}^{N}\left(x\right)\right)dxds\to \int_{0}^{t}\int f\left(x\right)\varphi \left(s,x\right)F\left(\varphi \left(s,x\right)\right)dxds.$$

Since F is a polynomial, we consider powers of $\rho_{\epsilon }*\eta$. To illustrate the approach, we first prove that

(7.30) $$\int_{0}^{t}\int f(x)\rho_{\epsilon }*\eta_{s}^{N}(x{)}^{2}dxds\to \int_{0}^{t}\int f(x)\varphi (s,x{)}^{2}dxds.$$

The convergence of higher powers will follow in an entirely analogous manner, but with more complex expressions.

The approach is standard. We fix τ>0 and, in keeping with our notation $\rho_{\epsilon }$, in this subsection, use $\rho_{\tau }$ to denote the symmetric Gaussian kernel with variance parameter $\tau^{2}$. Our strategy is to show that, up to an error that tends to zero as τ→0,

(7.31) $$\int_{0}^{t}\int f(z)\rho_{\epsilon }*\eta_{s}(z{)}^{2}dzds∼\int_{0}^{t}\int \int f\left(z\right)\left(\rho_{\epsilon }*\eta_{s}\right)\left(z\right)\rho_{\tau }\left(z-y\right)\left(\rho_{\epsilon }*\eta_{s}\right)\left(y\right)dzdyds.$$

Analogously, also up to an error that vanishes as τ→0,

(7.32) $$\int_{0}^{t}\int f(z)\varphi (s,z{)}^{2}dzds∼\int_{0}^{t}\int \int f\left(z\right)\varphi \left(s,z\right)\rho_{\tau }\left(z-y\right)\varphi \left(s,y\right)dzdyds.$$

On the other hand, weak convergence of $\rho_{\epsilon }*\eta$ (plus continuity of the mapping $(z,y)\to \left.f(z)\rho_{\tau }(z-y)\right)$ gives that

(7.33) $$\int_{0}^{t}\int \int f(z)\left(\rho_{\epsilon }*\eta_{s}\right)(z)\rho_{\tau }(z-y)\left(\rho_{\epsilon }*\eta_{s}\right)(y)dzdyds\to \int_{0}^{t}\int \int f(z)\varphi (s,z)\rho_{\tau }(z-y)\varphi (s,y)dzdyds.$$

Since τ is arbitrary, the convergence (7.30) will follow.

#### Proposition 7.9

*Under the conditions of Theorem 2.20, we have that along any convergent subsequence,*

(7.34) $$\;\underset{\epsilon \to 0}{lim\;sup}\;E\left[|\int_{0}^{t}\;\int f(y)\rho_{\epsilon }*\eta_{s}(y{)}^{2}dyds\right.\;\;\left.-\int_{0}^{t}\;\int \int f(z)\left(\rho_{\epsilon }*\eta_{s}\right)(z)\rho_{\tau }(z-y)\left(\rho_{\epsilon }*\eta_{s}\right)(y)dzdyds|\right]\leq C\tau ,$$

where C is independent of τ.

#### Proof

First note,

(7.35) $$\int_{0}^{t}\;E[|\langle f(y),{\left(\rho_{\epsilon }*\eta_{s}(y)\right)}^{2}dy\rangle -\int \int f(y)\left(\rho_{\epsilon }*\eta_{s}\right)(z)\rho_{\tau }(z-y)\left(\rho_{\epsilon }*\eta_{s}\right)(y)dzdy|]ds\;\;\leq ∥f{∥}_{\infty }\int \int_{0}^{t}\;E\left[\int \left\{\left|\left(\rho_{\epsilon }*\eta_{s}\right)(y)-\left(\rho_{\epsilon }*\eta_{s}\right)(z)\right|\rho_{\tau }(z-y)dz\right\}\left(\rho_{\epsilon }*\eta_{s}\right)(y)\right]dsdy.$$

Now proceed exactly as in the proof of Proposition 7.8. The only distinction is that $|p_{\epsilon^{2}}(y,z)-p_{\epsilon^{2}}(w,z)|$ is replaced by $\left|p_{\tau }(y,z)-p_{\tau }(w,z)\right|$ and the estimate $∥y-w∥p_{\tau^{2}}(y,w)\leq C\tau p_{2\tau^{2}}(y,w)$ replaces the corresponding statement with $\epsilon^{2}$ replacing $\tau^{2}$ in our previous argument. □

The extension of Proposition 7.9 to higher moments is straightforward, if notationally messy. For fixed (but arbitrary) τ, one shows that

$$\underset{\epsilon \to 0}{lim\;sup}\;E\left[|\int_{0}^{t}\;\int f(y)\rho_{\epsilon }*\eta_{s}^{N}(y{)}^{k}dyds\right.\;\;\left.-\int_{0}^{t}\;\int \cdots \int f\left(y_{1}\right)\rho_{\epsilon }*\eta_{s}^{N}\left(y_{1}\right)\prod_{i=2}^{k}\;\rho_{\tau }\left(y_{i}-y_{i-1}\right)\rho_{\epsilon }*\eta_{s}^{N}\left(y_{i}\right)dy_{k}\ldots dy_{1}ds|\right]\leq C\tau ,$$

as well as a corresponding statement with $\rho_{\epsilon }*\eta_{s}^{N}(x)$ replaced by φ(s,x) and then use weak convergence to see that, up to an error of order τ, any limit point of the sequence $\left\{\rho_{\epsilon }*\eta^{N}(x)dx\right\}$ solves (the weak form of) equation (2.16). Since τ was arbitrary, the proof of Theorem 2.20 is complete.

## 8 Proofs of results for the lookdown process and ancestral lineages

Now we turn to results about the lookdown process, first establishing the basic connection between the population process $\eta^{N}$ and the lookdown process $\xi^{N}$, Proposition 5.3, and then in the next section, convergence of the lookdown process itself.

### Proof [of Proposition 5.3]

This proposition is the content of the Markov Mapping Theorem, reproduced from Etheridge and Kurtz [2019] as Theorem A.1, applied to our situation. The function γ of that theorem is what we have called κ above, and the kernel α of that theorem is the transition function that assigns levels uniformly on [0,N] (in the first case) or as a Poisson process with Lesbegue intensity (in the limiting case). We need a continuous $\psi^{N}(\xi )\geq 1$ such that $\left|A^{N}f(\xi )\right|\leq c_{f}\psi^{N}(\xi )$ for all f in the domain of $A^{N}$ (and similarly a function ψ for A). We also need that applying the lookdown generator to a function and averaging over levels is equivalent to applying the population process generator to the function whose dependence on levels has been averaged out, a condition which we precisely state, and verify, in Lemmas A.2 and A.3 of the Appendix.

For finite N, taking f(ξ) of the form (5.6), we can use $\psi^{N}(\xi )=\langle C(1+u|F(x,\eta )|),\xi (dx,du)\rangle$ for an appropriate constant C. For the scaling limit, recall that the test functions f are of the form $f(\xi )=\prod_{(x,u)\in \xi }\;g(x,u)$ with g(x,u)=1 for $u\geq u_{0}$, and consulting (5.11), we see that most terms in Af(ξ) can be bounded as above by constant multiples of ⟨1,η⟩. However, the term involving F is, as usual, more troublesome. Since 0≤f(ξ)/g(x,u)≤1 for any $\left(x,u\right)\in \xi ,$

$$|f(\xi )\sum_{(x,u)\in \xi }\;F(x,\eta )u\frac{\partial_{u}g(x,u)}{g(x,u)}|\;\leq {∥\partial_{u}g∥}_{\infty }\sum_{(x,u)\in \xi }\;\left|F(x,\eta )u1_{u\leq u_{0}}\right|\;\;\leq {∥\partial_{u}g∥}_{\infty }e^{u_{0}}\sum_{(x,u)\in \xi }\;\left|F(x,\eta )ue^{-u}\right|.$$

The first line would be just what we want, except that ψ(ξ) cannot depend on f, and hence neither on $u_{0}$. So, the second line provides us with the required bound: we absorb ${∥\partial_{u}g∥}_{\infty }e^{u_{0}}$ into $c_{f}$ and take $\psi (\xi )=1+\left\langle 1+F(x,\eta )ue^{-u},\xi (dx,du)\right\rangle$. □

### 8.1 Tightness of the Lookdown Process

Now we turn to the main theorem on convergence of the lookdown process, Theorem 5.4, whose proof follows a similar pattern to that of convergence for the population processes in Section 6.2.

We first give a description of the lookdown process $\xi^{N}$ in terms of the lines of descent introduced in Section 5.2. Each line of descent gives birth to lines at higher levels at rate $2(N-u)c_{\theta }(x,\eta )$, and each such new line chooses a level uniformly from [u,N], a spatial location y from the kernel

(8.1) $$q^{m}(x,dy,\eta )=r(y,\eta )q(x,dy)/\int_{R^{d}}r(y,\eta )q(x,dy),$$

and the two lines swap spatial locations with probability 1/2; the level of each line of descent evolves according to equation (5.21).

It is evident from the description of the process (or, by differentiating in Definition 5.1) that

(8.2) $$\left\langle f,\xi_{t}^{N}\right\rangle =\left\langle f,\xi_{0}^{N}\right\rangle +M_{t}^{f}\;\;+\int_{0}^{t}\;\left\langle c_{\theta }\left(x,\eta_{s}^{N}\right)\int_{u}^{N}\;\int_{R^{d}}\;\left(f\left(y,u_{1}\right)+f\left(x,u_{1}\right)+f(y,u)-f(x,u)\right)q^{m}\left(x,dy,\eta_{s}^{N}\right)du_{1}\right.\;\;\left.\;+\left(c_{\theta }\left(x,\eta_{s}^{N}\right)u^{2}-b_{\theta }\left(x,\eta_{s}^{N}\right)u\right)\frac{d}{du}f(x,u),\xi_{s}^{N}(dx,du)\right\rangle ds,$$

where $M^{f}$ is a martingale with angle bracket process

(8.3) $${\langle M^{f}\rangle }_{t}=\int_{0}^{t}\langle c_{\theta }(x,\eta_{s}^{N})\rangle \int_{u}^{N}\int_{{\mathbb{R}}^{d}}[f{\left(y,u_{1}\right)}^{2}\;\;+{\left(f\left(x,u_{1}\right)+f(y,u)-f(x,u)\right)}^{2}]du_{1}q^{m}\left(x,dy,\eta_{s}^{N}\right),\xi_{s}^{N}(dx,du)\rangle ds.$$

#### Remark 8.1

*In addition to tightness of the measure-values processes* $\xi^{N}$, *the bounds used in the proofs below also imply tightness of the number of lines of descent and the number of births below a fixed level, and of the motion of individual lines of descent. In other words, the limiting “line of descent” construction of Section 5.2 holds.*

#### Proof [Proof of Theorem 5.4]

As in Section 6.2, the theorem will follow from tightness and characterization of the limit points. This time, the processes $\xi^{N}$ take values in $ℳ(\overset{-}{R^{d}}\times [0,\infty )$), the space of locally finite measures on space × levels. (They will in fact be point measures, including the limit, but that is a consequence of this theorem.) Again, tightness follows from a compact containment condition, tightness of one-dimensional distributions, and an application of Ethier and Kurtz [1986] Theorem 3.9.1.

Lines of descent can escape to infinite level in finite time, and so we endow $ℳ(\overset{-}{R^{d}}\times [0,\infty ))$ with the vague topology “in the level coordinate”, induced by test functions on $\overset{-}{R^{d}}\times [0,\infty )$ of the form g(x)h(u), where $g\in C_{b}(\overset{-}{R^{d}})$ is bounded and continuous and $h\in C_{c}([0,\infty ))$ is compactly supported (following, e.g., Etheridge and Kurtz [2019], Condition 2.1). In several places below we require a dense subset of $C_{b}(ℳ(\overset{-}{R^{d}}\times [0,\infty )))$, the bounded, continuous functions on $ℳ(\overset{-}{R^{d}}\times [0,\infty ))$. The functions ξ↦⟨f,ξ⟩ do not form not a dense subset of $C_{b}(ℳ(\overset{-}{R^{d}}\times [0,\infty )))$, but they do separate points and vanish nowhere, i.e., for any $\xi_{1}$ and $\xi_{2}$ there is an f with $\left\langle f,\xi_{1}\right\rangle \neq \left\langle f,\xi_{2}\right\rangle$, and a g such that $\left\langle g,\xi_{1}\right\rangle \neq 0$. Therefore, by the Stone-Weierstrass theorem, the algebra they generate is dense in $C_{b}(ℳ(\overset{-}{R^{d}}\times [0,\infty )))$ (with respect to uniform convergence on compact subsets). Topologized in this way, the space $ℳ(\overset{-}{R^{d}}\times [0,\infty ))$ is completely metrizable, and we may choose a countable set of nonnegative $f_{k}$, each supported on $R^{d}\times \left[0,u_{k}\right]$ for some $u_{k}<\infty$, such that a subset $K\subset ℳ(\overset{-}{R^{d}}\times [0,\infty ))$ is relatively compact if and only if ${sup}_{\xi \in K}\;\left\langle f_{k},\xi \right\rangle <\infty$ for each k. (To see this, use Theorem A.2.3 of Kallenberg [1997] and the first argument made in the proof of Lemma B.3.) Below, Lemma 8.4 proves exactly this, and therefore compact containment. Here we have compactified $R^{d}$ for convenience (since it turned out to be straightforward to show that mass does not escape to infinity in space); however, we need to use the vague topology “in the level direction” because *levels* may escape to infinity in finite time in the limit.

In order to apply Ethier and Kurtz [1986] Theorem 3.9.1 we require that ${\left\{{\left(F\left(\xi_{t}^{N}\right)\right)}_{t\geq 0}\right\}}_{N}$ is tight as a sequence of real-valued càdlàg processes, for all F in a subset of $C_{b}(ℳ(\overset{-}{R^{d}}\times [0,\infty )))$ that is dense with respect to uniform convergence on compact subsets. Lemma 8.5 shows that ${\left\{\left\langle f,\xi_{t}^{N}\right\rangle \right\}}_{N}$ is a tight sequence for any $f:\overset{-}{R^{d}}\times [0,\infty )\to R$ with compact support in the level direction. Since as above the algebra generated by the functions ξ↦⟨f,ξ⟩ is dense in $C_{b}(ℳ(\overset{-}{R^{d}}\times [0,\infty )))$, it suffices to show that tightness for the processes $\left\langle f,\xi_{t}^{N}\right\rangle$ extends to finite sums and products of these processes, which is shown in Lemma B.3. The fact that martingale properties are preserved under passage to the limit is straightforward, and can be proved in a way analogous to Lemma 6.6; we omit the proof. Finally, we must show that the limiting lookdown process ξ projects to the limiting process η, i.e., a solution of the martingale problem in Theorem 2.10. Let $N_{k}\to \infty$ be a sequence along which $\xi^{N_{k}}$ converges. By Theorem 2.10, there is a subsequence $N_{k(j)}$ along which the projected population processes $\eta^{N_{k(j)}}$ converge, and the limit solves the martingale problem. Thus any limit point of $\xi^{N}$ projects to a population process η solving the martingale problem of Theorem 2.10. □

What we need for compact containment will come from the following Lemma. The generality is unimportant – for concreteness one may take $h(u)=e^{-u}$.

#### Lemma 8.2

*Let* h *be a positive, continuous, nonincreasing, differentiable function on* [0,∞) *such that* $\int_{0}^{\infty }\;\int_{u}^{\infty }\;h(v)dvdu,\;\int_{0}^{\infty }\;u^{2}\left|h^{'}(u)\right|du$, and $\int_{0}^{\infty }\;h(u{)}^{2}du$ *are all finite. Suppose that Assumptions 2.8 hold, and that* θ/N→α *and* $\xi_{0}^{N}\to \xi_{0}$ *weakly as* N→∞, *where each* $\xi_{0}^{N}$ *is conditionally uniform given* $\eta_{0}^{N}$ *in the sense of* (5.12) *and* $\xi_{0}$ *is conditionally* Poisson given $\eta_{0}$ *in the sense of* (5.13). Then for any T there exists a constant K(T) *such that for all* M>0,

$$\underset{N\to \infty }{lim\;sup}\;P\left\{\underset{0\leq t\leq T}{sup}\;\left\langle h,\xi_{t}^{N}\right\rangle >M\right\}<\frac{K(T)}{M}.$$

We postpone the proof of this Lemma until we have shown how it yields compact containment. First, we show that this implies compact containment of the processes ${\left(\left\langle f,\xi_{t}^{N}\right\rangle \right)}_{0\leq t\leq T}$ for arbitrary compactly supported f.

#### Lemma 8.3

*Suppose* $f\in C({\overset{-}{R}}^{d}\times [0,\infty ))$ *and there is a* $u_{f}$ *such that if* $u\geq u_{f}$ *then* ${sup}_{x}\;f(x,u)=0$. *Under the assumptions of* Lemma 8.2, *for any* T *there exists a constant* K(f,T) *such that for all* M>0,

$$\underset{N\to \infty }{lim\;sup}\;P\left\{\underset{0\leq t\leq T}{sup}\;\left\langle f,\xi_{t}^{N}\right\rangle >M\right\}<\frac{K(f,T)}{M}.$$

#### Proof [of Lemma 8.3]

Let h be as in Lemma 8.2, so there is a $c_{f}<\infty$ such that $f(x,u)\leq c_{f}h(u)$ for all x and u. Therefore, ⟨f,ξ⟩≤⟨h,ξ⟩, and so by Lemma 8.2,

$$\underset{N\to \infty }{lim\;sup}\;P\left\{\underset{0\leq t\leq T}{sup}\;\left\langle f,\xi_{t}^{N}\right\rangle >M\right\}\leq \underset{N\to \infty }{lim\;sup}\;P\left\{\underset{0\leq t\leq T}{sup}\;\left\langle h,\xi_{t}^{N}\right\rangle >M/c_{f}\right\}<\frac{K\left(T\right)c_{f}}{M}.$$

□

#### Lemma 8.4 (Compact containment for ξ)

Let $f_{1},f_{2},\ldots$ be a sequence of functions each satisfying the conditions of Lemma 8.3. Under the assumptions of Lemma 8.2, for any T and δ>0 there exists a sequence $\left(C_{1},C_{2},\ldots \right)$ of finite constants such that

(8.4) $$\underset{N\to \infty }{lim\;sup}\;P\left\{\underset{0\leq t\leq T}{sup}\;\left\langle f_{k},\xi_{t}^{N}\right\rangle >C_{k}\;\text{for}\;\text{some}\;k\geq 1\right\}<\delta .$$

In other words, the processes $\xi^{N}$ stay in the set

$$\{\xi \in ℳ(\overset{-}{R^{d}}\times [0,\infty )):\left\langle f_{k},\xi \right\rangle \leq C_{k}\;\text{for}\;\text{all}\;k\geq 1\},$$

for all 0≤t≤T with uniformly high probability, a set which (as discussed in the proof of Theorem 5.4) is relatively compact for an appropriate choice of ${\left\{f_{k}\right\}}_{k\geq 1}$.

#### Proof [of Lemma 8.4]

By a union bound,

$$\mathbb{P}\left\{\underset{0\leq t\leq T}{\sup}\langle f_{k},\xi_{t}^{N}\rangle >C_{k}\;\text{for}\;\text{some}\;k\geq 1\right\}\leq \sum_{k\geq 1}\mathbb{P}\left\{\underset{0\leq t\leq T}{\sup}\langle f_{k},\xi_{t}^{N}\rangle >C_{k}\right\},$$

so (8.4) follows by taking $C_{k}=2^{k-1}K\left(f_{k},T\right)/\delta$ and using Lemma 8.3. □

Finally, we prove the key lemma.

#### Proof [of Lemma 8.2]

Applied to f(x,u)=h(u), the martingale representation (8.2) is

$$\left\langle h,\xi_{t}^{N}\right\rangle =\left\langle h,\xi_{0}^{N}\right\rangle \;+M_{t}^{h}\;\;+\int_{0}^{t}\;\left\langle 2c_{\theta }\left(x,\eta_{s}^{N}\right)\int_{u}^{N}\;h(v)dv,\xi_{s}^{N}(dx,du)\right\rangle ds\;\;+\int_{0}^{t}\;\left\langle \left(c_{\theta }\left(x,\eta_{s}^{N}\right)u^{2}-b_{\theta }\left(x,\eta_{s}^{N}\right)u\right)h^{'}(u),\xi_{s}^{N}(dx,du)\right\rangle ds,$$

where $M_{t}^{h}$ is a martingale with angle bracket process

$${\left\langle M^{h}\right\rangle }_{t}=\int_{0}^{t}\left\langle 2c_{\theta }\left(x,\eta_{s}^{N}\right)\int_{u}^{N}\;h(v{)}^{2}dv,\xi_{s}^{N}(dx,du)\right\rangle ds.$$

Now, note that $0\leq c_{\theta }\left(x,\eta_{x}^{N}\right)\leq C_{a}<\infty$ and $b_{\theta }\left(x,\eta_{s}^{N}\right)\leq C_{b}<\infty$, and we have assumed that $h^{'}(u)\leq 0$ (since h is nonincreasing), so we may bound

(8.5) $$\left\langle h,\xi_{t}^{N}\right\rangle \leq \left\langle h,\xi_{0}^{N}\right\rangle +M_{t}^{h}\;\;+\int_{0}^{t}\;\left\langle 2C_{a}\int_{u}^{\infty }\;h(v)dv+\left(C_{a}u^{2}+C_{b}u\right)\left|h^{'}(u)\right|,\xi_{s}^{N}(dx,du)\right\rangle ds.$$

Now, since $\xi_{t}^{N}$ is conditionally uniform given $\eta_{t}^{N}$ in the sense of (5.12), we know that for compactly supported $f,E\left[\left\langle f,\xi_{t}^{N}\right\rangle \right]=E\left[\left\langle {\tilde{f}}_{N},\eta_{t}^{N}\right\rangle \right]$, where ${\tilde{f}}_{N}(x)=\int_{0}^{N}f(x,u)du$. By our assumptions on h, we know that

$$\int_{0}^{\infty }\left(2C_{a}\int_{u}^{\infty }\;h(v)dv+\left(C_{a}u^{2}+C_{b}u\right)\left|h^{'}(u)\right|\right)du<C$$

for some C<∞, and so (by dominated convergence)

$$E\left[\left\langle h,\xi_{t}^{N}\right\rangle \right]\leq E\left[\left\langle h,\xi_{0}^{N}\right\rangle \right]+C\int_{0}^{t}E\left[\left\langle 1,\eta_{s}^{N}\right\rangle \right]ds,$$

which we know by Lemma 6.1 is bounded by $C_{0}e^{C_{1}t}$ for some other constants $C_{0}$ and $C_{1}$.

Now consider the maximum. By (8.5), using that the integrand is nonnegative,

$$\underset{0\leq t\leq T}{sup}\;\left\langle h,\xi_{t}^{N}\right\rangle \leq \left\langle h,\xi_{0}^{N}\right\rangle +\underset{0\leq t\leq T}{sup}\;M_{t}^{h}\;\;+\int_{0}^{T}\;\left\langle 2C_{a}\int_{u}^{\infty }\;h(v)dv+\left(C_{a}u^{2}+C_{b}u\right)\left|h^{'}(u)\right|,\xi_{s}^{N}(dx,du)\right\rangle ds.$$

Since $\sqrt{x}\leq 1+x$ for x≥0, the Burkholder-Davis-Gundy inequality tells us that there is a $C^{'}$ such that

$$E\left[\underset{0\leq t\leq T}{sup}\;M_{t}^{h}\right]\;\leq C^{'}\left(1+E\left[{\left[M^{h}\right]}_{T}\right]\right)\;\;\leq C^{'}\left(1+\int_{0}^{T}\;E\left[\left\langle 2c_{\theta }\left(x,\eta_{s}^{N}\right)\int_{u}^{\infty }\;h(v{)}^{2}dv,\xi_{s}^{N}(dx,du)\right\rangle \right]ds\right)\;\;\leq C^{'}\left(1+2C_{a}\int_{0}^{\infty }\;h(v{)}^{2}dv\int_{0}^{T}\;E\left[\left\langle 1,\xi_{s}^{N}(dx,du)\right\rangle \right]ds\right)\;\;\leq C_{2}e^{C_{1}T},$$

for a constant $C_{2}$ which is finite by our assumption that $\int_{0}^{\infty }\;h(v{)}^{2}dv<\infty$

Therefore,

$$E\left[\underset{0\leq t\leq T}{sup}\;\left\langle h,\xi_{t}^{N}\right\rangle \right]\leq E\left[\left\langle h,\xi_{0}^{N}\right\rangle \right]+\left(C_{2}+C_{0}/C_{1}\right)e^{C_{1}T},$$

and so

$$P\left\{\underset{0\leq t\leq T}{sup}\;\left\langle h,\xi_{t}^{N}\right\rangle >K\right\}\leq \frac{E\left[\left\langle h,\xi_{0}^{N}\right\rangle \right]+\left(C_{2}+C_{0}/C_{1}\right)e^{C_{1}T}}{K}.$$

□

#### Lemma 8.5

*Let* f *be a bounded, continuous real-valued function on* $R^{d}\times [0,\infty )$ *with uniformly bounded first and second derivatives for which there exists a* $u_{0}$ *such that if* $u>u_{0}$ *then* f(x,u)=0. *Then, the sequence of real-valued processes* ${\left(\left\langle f,\xi_{t}^{N}\right\rangle \right)}_{t\geq 0}$ *for* N≥1 *is tight in* ${𝒟}_{\left[0,\infty \right)}(R)$.

#### Proof [of Lemma 8.5]

Again, we use the Aldous-Rebolledo criterion. Tightness of $\left\langle f,\xi_{t}\right\rangle$ for a fixed t follows from Lemma 8.3, so we need only prove conditions analogous to (6.9) and (6.10) applied to the martingale representation of equations (8.2) and (8.3). Rewriting (8.2) with $c_{\theta }=c_{\theta }\left(x,\eta_{s}\right),$

$$\left\langle f,\xi_{t}\right\rangle =\left\langle f,\xi_{0}\right\rangle +M_{t}^{f}+\int_{0}^{t}\;\left\langle c_{\theta }\int_{u}^{N}\;\int \left(f\left(y,u_{1}\right)+f\left(x,u_{1}\right)\right)q^{m}(x,dy,\eta )du_{1}\right.\;\;+c_{\theta }(N-u)\left.\int_{0}^{t}\;(f(y,u)-f(x,u))q^{m}(x,dy,\eta )+\left(c_{\theta }u^{2}-b_{\theta }u\right)\frac{d}{du}f,\xi_{s}\right\rangle ds.$$

The bounds analogous to (6.9) and (6.10) follow as in the proof of Lemma 6.3: for instance, observe that using that $c_{\theta }\leq C_{a}$ for some $C_{a}$, the predictable part of this semimartingale decomposition is bounded by

$$\left\langle 2C_{a}∥f{∥}_{\infty }u_{f}+(1-u/N)\gamma B_{f}^{\theta }+\left(C_{a}u^{2}-b_{\theta }u\right)\frac{d}{du}f,\xi_{s}\right\rangle ,$$

the last term of which is bounded by

$$\langle C_{a}u_{f}^{2}+\underset{x}{sup}\;\left|b_{\theta }\left(x,\eta_{s}\right)\right|u_{f}{∥\frac{d}{du}f∥}_{\infty }\rangle ,$$

which can be bounded as we did for (6.9). □

### 8.2 Motion of ancestral lineages

In this section we prove Theorem 2.23. The argument follows directly from the discussion in Section 5.3.

#### Proof [of Theorem 2.23]

For brevity, in the proof we write γ(x) or γ for γ(x,η).

Here we have taken the high-density, deterministic limit (so, θ,N→∞ and θ/N→0). We first proceed informally, as if the limiting process has a density $\varphi_{t}(x)$ at location x and time t (which it may not), and follow this with an integration against test functions to make the argument rigorous. Let Y denote the spatial motion followed by a single line of descent. Above equation (5.19), we showed that Y is a diffusion with generator at time s

$${ℒ}_{s}^{Y}g(x)=\gamma \left(x,\eta_{s}\right)\left(ℬ\left(r\left(\cdot ,\eta_{s}\right)g(\cdot )\right)(x)-g(x)ℬr\left(x,\eta_{s}\right)\right).$$

The diffusion is time-inhomogeneous if the density is not constant in time. Let $\varphi_{t}(x)$ be the limiting density, which is a weak solution to (1.1), $\partial_{t}\varphi_{t}=r{ℬ}^{*}\left[\varphi_{t}\gamma \right]+\varphi_{t}F$. Formally, the intensity of individuals at y at time t that are descended from individuals that were at x at time s (with s<t) is

(8.6) $$\varphi_{s}\left(x\right)E_{s,x}\left[\exp\left(\int_{s}^{t}\;\left(F+\gamma ℬr\right)\left(Y_{u}\right)du\right)1_{Y_{t}=y}\right]dy,$$

where the subscript s,x in the expectation indicates that $Y_{s}=x$. To see why this should be true, suppose that an ancestor at time s has level v. Conditional on its spatial motion ${\left\{Y_{u}\right\}}_{s\leq u\leq t}$, its level at time t will be $v\;exp\left(-\int_{s}^{t}\;(F+\gamma ℬr)\left(Y_{u}\right)du\right)$. This will be less than a given level λ if $v<\lambda \;exp\left(\int_{s}^{t}\;(F+\gamma ℬr)\left(Y_{u}\right)du\right)$. The intensity of levels at y that are descended from individuals at x can therefore be obtained as the limit as λ→∞ of 1/λ times the number of levels at x at time s with $u<\lambda \;exp\left(\int_{s}^{t}\;(F+\gamma ℬr)\left(Y_{u}\right)du\right)$ and for which the corresponding individual is at y at time t, which is precisely the quantity in (8.6).

By our construction in Section 5.3, when we integrate (8.6) with respect to x we recover $\varphi_{t}(y)dy$. Consider an individual sampled at location y at time t, and write p(t,s,y,x) for the probability density that their ancestor at time s was at x. As a consequence of (8.6), still formally,

(8.7) $$p\left(t,s,y,x\right)=\frac{\varphi_{s}\left(x\right)}{\varphi_{t}\left(y\right)}E_{s,x}\left[\exp\left(\int_{s}^{t}\;\left(F+\gamma ℬr\right)\left(Y_{u}\right)du\right)1_{Y_{t}=y}\right]\;\text{for}\;s<t.$$

To make (8.7) meaningful, we multiply by suitable test functions f and g and integrate.

$$\int \int f(y)\varphi_{t}(y)p(t,s,y,x)g(x)dydx\;\;=\int g(x)\varphi_{s}(x)E_{x,s}\left[exp\left(\int_{s}^{t}\;(F+\gamma ℬr)\left(Y_{u}\right)du\right)f\left(Y_{t}\right)\right]dx.$$

Writing ${\hat{T}}_{t,s}$ for the time-inhomogeneous semigroup corresponding to the motion of ancestral lineages backwards in time (that is, $\left.{\hat{T}}_{t,s}f(y)=\int p(t,s,x,y)f(x)dy\right)$, we can write this as

(8.8) $$\int f\left(y\right)\varphi_{t}\left(y\right){\hat{T}}_{t,s}g\left(y\right)dy=\int g\left(x\right)\varphi_{s}\left(x\right)E_{s,x}\left[\exp\left(\int_{s}^{t}\;\left(F+\gamma ℬr\right)\left(Y_{u}\right)du\right)f\left(Y_{t}\right)\right]dx.$$

Next, we will differentiate this equation with respect to t. There are two terms in the product on the left-hand side that depend on t, so if we use that $\partial_{t}\varphi_{t}=r{ℬ}^{*}\left[\varphi_{t}\gamma \right]+\varphi F$ (in a weak sense), and write ${ℒ}_{u}$ for the generator of ${\hat{T}}_{t,s}$ at time t=u so that ${\left.\partial_{t}{\hat{T}}_{t,s}g(y)\right|}_{t=s}={ℒ}_{s}g(y)$, then

$$\frac{d}{dt}\int f(y)\varphi_{t}(y){\left.{\hat{T}}_{t,s}g(y)dy\right|}_{t=s}\;\;=\int f\left(y\right)\left\{\varphi_{s}\left(y\right){ℒ}_{s}g\left(y\right)+\left[r\left(y\right){ℬ}^{*}\left(\gamma \varphi_{s}\right)\left(y\right)+\varphi_{s}\left(y\right)F\left(y\right)\right]g\left(y\right)\right\}dy.$$

As for the right-hand side, since $Y_{s}=x$ under $E_{x,s}$,

$${\left.\frac{d}{dt}E_{x,s}\left[exp\left(\int_{s}^{t}\;(F+\gamma ℬr)\left(Y_{u}\right)du\right)f\left(Y_{t}\right)\right]\right|}_{t=s}=[F(x)+\gamma (x)ℬr(x)]f(x)+{ℒ}_{s}^{Y}f(x).$$

Therefore, the derivative of (8.8) (with respect to t, evaluated at t=s) is

$$\int f(y)\left\{\varphi_{s}(y){ℒ}_{s}g(y)+\left(r(y){ℬ}^{*}\left(\gamma \varphi_{s}\right)(y)+\varphi_{s}(y)F(y)\right)g(y)\right\}dy\;\;=\int g(x)\varphi_{s}(x)\left({ℒ}_{s}^{Y}f(x)+[F(x)+\gamma (x)ℬr(x)]f(x)\right)dx\;\;=\int f\left(x\right)\left({\left({ℒ}_{s}^{Y}\right)}^{*}\left(\varphi_{s}g\right)\left(x\right)+\left[F\left(x\right)+\gamma \left(x\right)ℬr\left(x\right)\right]\varphi_{s}\left(x\right)g\left(x\right)\right)dx,$$

where ${\left({ℒ}_{s}^{Y}\right)}^{*}$ is the adjoint of ${ℒ}_{s}^{Y}$. Since f was arbitrary,

$${ℒ}_{s}g=\frac{1}{\varphi_{s}}\left[{\left({ℒ}_{s}^{Y}\right)}^{*}\left(\varphi_{s}g\right)+\gamma \varphi_{s}gℬ(r)-rg{ℬ}^{*}\left(\gamma \varphi_{s}\right)\right].$$

(Note that the $\varphi_{s}Fg$ terms have cancelled.) Since the adjoint of ${ℒ}_{s}^{Y}$ is

$${\left({ℒ}_{s}^{Y}\right)}^{*}f=r{ℬ}^{*}\left(\gamma f\right)-\gamma fℬr,$$

we can rewrite the generator of a lineage as

$${ℒ}_{s}g=\frac{r}{\varphi_{s}}\left[{ℬ}^{*}\left(\gamma \varphi_{s}g\right)-g{ℬ}^{*}\left(\gamma \varphi_{s}\right)\right].$$

This is equation (2.17).

To simplify to equation (2.18), first define $𝒟f(x)=\sum_{ij}\;C_{ij}\partial_{ij}f(x)$, and so the adjoint of 𝒟 is

$${𝒟}^{*}f(x)=\sum_{ij}\partial_{ij}\left(C_{ij}f(x)\right).$$

Note that ${𝒟}^{*}$ satisfies the following identity:

$${𝒟}^{*}(fg)\;=\sum_{ij}\;\left\{g\partial_{ij}\left(C_{ij}f\right)+2f\partial_{i}\left(C_{ij}\right)\partial_{j}(g)+2C_{ij}\partial_{i}(f)\partial_{j}(g)+C_{ij}f\partial_{ij}g\right\}\;\;=g{𝒟}^{*}f+2f\vec{c}\cdot \nabla g+2(C\nabla f)\cdot \nabla g+f𝒟g,$$

where ${\vec{c}}_{j}=\sum_{i}\partial_{j}C_{ij}$. So, with $f=\gamma \varphi_{s}$

$${ℒ}_{s}g\;=\frac{r}{\varphi_{s}}\left[{𝒟}^{*}(\gamma \varphi_{s}g)-\nabla \cdot (\gamma \varphi_{s}g\vec{b})-g{𝒟}^{*}(\gamma \varphi_{s})+g\nabla \cdot \left(\gamma \varphi_{s}\vec{b}\right)\right]\;\;=\frac{r}{\varphi_{s}}\left[\gamma \varphi_{s}𝒟g+2\gamma \varphi_{s}\vec{c}\cdot \nabla g+2\left(C\nabla \left(\gamma \varphi_{s}\right)\right)\cdot \nabla g-\gamma \varphi_{s}\vec{b}\cdot \nabla g\right]\;\;=r\gamma \left[𝒟g+2\vec{c}\cdot \nabla g+2\left(C\nabla \;log\left(\gamma \varphi_{s}\right)\right)\cdot \nabla g-\vec{b}\cdot \nabla g\right],$$

which is equation (2.18). □

#### Proof [of Corollary 2.26]

For the moment, we will write r(x) for r(x,η) and γ(x) for γ(x,η). First note that since in this case the semigroup does not depend on time, we can write $ℒ={ℒ}_{s}$, and

$$ℒf=\sigma^{2}r\gamma \left(\Delta f+\nabla \left(2\;log(\gamma \varphi )-h/\sigma^{2}\right)\cdot \nabla f\right).$$

Now, observe that

$$\int_{R^{d}}e^{H\left(x\right)}f\left(x\right)\left(\Delta +\nabla H\left(x\right)\cdot \nabla \right)g\left(x\right)dx=-\int_{R^{d}}e^{H\left(x\right)}\left\{\nabla f\left(x\right)\cdot \nabla g\left(x\right)\right\}dx,$$

so that by choosing $H\left(x\right)=2\;log(\gamma (x)\varphi (x))-h(x)/\sigma^{2}$ and

$$\pi \left(x\right)=e^{H\left(x\right)}/\left(\sigma^{2}r\left(x\right)\gamma \left(x\right)\right)=\frac{\gamma (x)\varphi (x{)}^{2}e^{-h\left(x\right)/\sigma^{2}}}{\sigma^{2}r\left(x\right)},$$

we have that

$$\int_{R^{d}}\pi \left(x\right)f\left(x\right)ℒg\left(x\right)dx=-\int_{R^{d}}e^{H\left(x\right)}\nabla f\left(x\right)\cdot \nabla g\left(x\right)dx.$$

Since this is symmetric in f and g, the process Y is reversible with respect to π; the constant factor of $\sigma^{2}$ does not affect the result. □

## A Markov Mapping Theorem

The following appears as Theorem A.2 in Etheridge and Kurtz [2019], specialized slightly here to the case that the processes are càdlàg and have no fixed points of discontinuity. For an $S_{0}$-valued, measurable process $Y,\;{\hat{ℱ}}_{t}^{Y}$ denotes the completion of the σ-algebra generated by Y(0) and $\left\{\int_{0}^{r}\;h(Y(s))ds,r\leq t,h\in B\left(S_{0}\right)\right\}$. Also, let $D_{S}[0,\infty )$ denote the space of càdlàg, S-valued functions with the Skorohod topology, and $M_{S}[0,\infty )$ the space of Borel measurable functions from [0,∞) to S, topologized by convergence in Lesbegue measure. For other definitions see Etheridge and Kurtz [2019].

### Theorem A.1 (Markov Mapping Theorem)

*Let* (S,d) *and* $\left(S_{0},d_{0}\right)$ *be complete, separable metric spaces. Let* $A\subset C_{b}(S)\times C(S)$ *and* ψ∈C(S), ψ≥1. *Suppose that for each* f∈𝒟(A) *there exists* $c_{f}$ *such that*

$$|Af(x)|\leq c_{f}\psi (x),\;x\in A,$$

and define $A_{0}f(x)=Af(x)/\psi (x).$

Suppose that $A_{0}$ is a countably determined pre-generator, and suppose that $𝒟(A)=𝒟\left(A_{0}\right)$ is closed under multiplication and is separating. Let $\gamma :S\to S_{0}$ be Borel measurable, and let α be a transition function from $S_{0}$ into $S\left(y\in S_{0}\to \alpha (y,\cdot )\in 𝒫(S)\right.$ is Borel measurable) satisfying ∫h∘γ(x)α(y,dx)=h(y) for $y\in S_{0}$ and $h\in B\left(S_{0}\right)$, that is, $\alpha \left(y,\gamma^{-1}(y)\right)=1$. Assume that $\tilde{\psi }(y)\equiv \int_{S}\psi (z)\alpha (y,dz)<\infty$ for each $y\in S_{0}$ and define

(A.1)
$$C=\left\{\int_{S}\;f(z)\alpha (\cdot ,dz),\int_{S}\;Af(z)\alpha (\cdot ,dz):f\in 𝒟(A)\right\}.$$

*Let* $\mu_{0}\in 𝒫\left(S_{0}\right)$ *and define* $\nu_{0}=\int \alpha (y,\cdot )\mu_{0}(dy)$.

- *If* $\tilde{Y}$ *satisfies* $\int_{0}^{t}\;E[\tilde{\psi }(\tilde{Y}(s))]ds<\infty$ for all t≥0 and $\tilde{Y}$ is a solution of the martingale problem for $\left(C,\mu_{0}\right)$, then there exists a solution X of the martingale problem for $\left(A,\nu_{0}\right)$ such that $\tilde{Y}$ has the same distribution on $M_{S_{0}}[0,\infty )$ as Y=γ∘X. If Y and $\tilde{Y}$ are cádlág, then Y and $\tilde{Y}$ have the same distribution on $D_{S_{0}}[0,\infty )$.
- For t≥0,
  $$P\left\{X(t)\in \Gamma |{\hat{ℱ}}_{t}^{Y}\right\}=\alpha \left(Y\left(t\right),\Gamma \right),\;\;\text{for}\;\Gamma \in ℬ\left(S\right).$$
- If, in addition, uniqueness holds for the martingale problem for $\left(A,\nu_{0}\right)$, then uniqueness holds for the $M_{S_{0}}[0,\infty )$-martingale problem for $\left(C,\mu_{0}\right)$. If $\tilde{Y}$ has sample paths in $D_{S_{0}}[0,\infty )$ then uniqueness holds for the $D_{S_{0}}[0,\infty )$-martingale problem for $\left(C,\mu_{0}\right)$.
- If uniqueness holds for the martingale problem for $\left(A,\nu_{0}\right)$ then Y is a Markov process.

In our application, we have taken S to be the space of locally finite counting measures on $R^{d}\times [0,N)$ or on $R^{d}\times [0,\infty )$, and $S_{0}$ the space of finite measures on ${\overset{-}{R}}^{d}$. Then, A corresponds to the generator for the lookdown process (i.e., either $A^{N}$ or A), and C corresponds to the generator for the spatial population process (i.e., either ${𝒫}^{N}$ or 𝒫). The “γ” of the theorem is our spatial projection operator that we have called $\kappa^{N}$ or κ, and the “α“ of the theorem will be named $\Gamma^{N}$ or Γ below. Finally, “X” of the theorem is our lookdown process, ξ, and “Y” is our spatial process, η.

### A.1 Lookdown Generators

In this section we verify one of the conditions of the Markov Mapping Theorem, namely, that “integrating out levels” in the generator of the lookdown process we obtain the generator of the projected process. In the notation of the theorem, we are verifying that C defined in (A.1) is in fact ${𝒫}^{N}$ (if defined with $A^{N}$) or ${𝒫}^{\infty }$ (if defined with A). We will work with test functions of the form

(A.2)
$$f(\xi )=\prod_{(x,u)\in \xi }g(x,u)=\exp(\langle \log g,\xi \rangle ),$$

where 0≤g≤1 and g(x,u)=1 for all $u\geq u_{g}$ for some $u_{g}<\infty$. Furthermore, recall that $\kappa^{N}(\xi )(\cdot )=\xi (\cdot \times [0,N))/N$ is the “spatial projection operator”, and define the transition function $\Gamma^{N}:{ℳ}_{F}\left(R^{d}\right)\to ℳ\left(R^{d}\times [0,N)\right)$ so that for $\eta \in {ℳ}_{F}\left(R^{d}\right)$, if ${\hat{g}}_{N}(x)=\int_{0}^{N}g(x,u)du/N$, then

$$F_{g}^{N}(\eta )\;:=\int f(\xi )\Gamma^{N}(\eta ,d\xi )\;\;=exp\left(N\left\langle log\frac{1}{N}\int_{0}^{N}\;g(x,u)du,\eta (dx)\right\rangle \right)\;\;=exp\left(N\left\langle log{\hat{g}}_{N}(x),\eta (dx)\right\rangle \right),$$

i.e., $\Gamma^{N}$ assigns independent labels on [0,N] to each of the points in η. It follows from Lemma 6.5 that for test functions of this form the generator of $\eta_{t}^{N}$ is

(A.3)
$${𝒫}^{N}F_{g}^{N}(\eta )=F_{g}^{N}(\eta )N\theta \langle \gamma (x,\eta )\;\int r(z,\eta )\left({\hat{g}}_{N}(z)-1\right)q_{\theta }(x,dz)\;\;\left.+\mu_{\theta }(x,\eta )\left(\frac{1}{{\hat{g}}_{N}(x)}-1\right),\eta (dx)\right\rangle .$$

(Note that f here differs from the f used in Lemma 6.5 so as to agree with standard usage in the literature on lookdown processes.) The generator of $\xi_{t}^{N}$ is $A^{N}$, defined in equation (5.10).

#### Lemma A.2

*For all finite counting measures* η *on* $R^{d}$, *if* f *is of the form* (A.2), *then*

(A.4)
$$\int A^{N}f\left(\xi \right)\Gamma^{N}\left(\eta ,d\xi \right)={𝒫}^{N}F_{g}^{N}\left(\eta \right).$$

For the limiting process, recall that $\kappa (\xi )(\cdot )={lim}_{u\to \infty }\;\xi (\cdot \times [0,u))/u$ is the “spatial projection operator”, and define the probability kernel $\Gamma :{ℳ}_{F}\left(R^{d}\right)\to ℳ\left(R^{d}\times [0,\infty )\right)$ so that for $\eta \in {ℳ}_{F}\left(R^{d}\right)$, defining $\tilde{g}(x)=\int_{0}^{\infty }\;(g(x,u)-1)du,$

$$F_{g}(\eta )\;:=\int f(\xi )\Gamma (\eta ,d\xi )\;\;=exp\left(\left\langle \int_{0}^{\infty }\;(g(x,u)-1)du,\eta (dx)\right\rangle \right)\;\;=e^{\left\langle \tilde{g}(x),\eta (dx)\right\rangle }.$$

i.e., Γ(η,⋅) is the distribution of a conditionally Poisson process with intensity a product of η and Lebesgue measure. It again follows from Lemma 6.5 that for test functions of this form the generator of $\eta_{t}$ is

(A.5)
$${𝒫}^{\infty }F_{g}\left(\eta \right)=F_{g}\left(\eta \right)\left\langle \gamma \left(x,\eta \right)ℬ\left(\tilde{g}\left(\cdot \right)r\left(\cdot \right)\right)\left(x\right)+F\left(x,\eta \right)\tilde{g}\left(x\right)+\alpha \gamma \left(x,\eta \right)r\left(x,\eta \right){\tilde{g}}^{2}\left(x\right),\eta \left(dx\right)\right\rangle .$$

The generator of $\xi_{t}$ is A, defined in equation (5.11).

#### Lemma A.3

*For all* $\eta \in {ℳ}_{F}\left(R^{d}\right)$, if f is of the form (A.2), *then*

(A.6)
$$\int Af\left(\xi \right)\Gamma \left(\eta ,d\xi \right)={𝒫}^{\infty }F_{g}\left(\eta \right).$$

#### Proof [of Lemma A.2]

First, break the generator $A^{N}$ into three parts,

$$A_{1}^{N}f(\xi )=f(\xi )\sum_{(x,u)\in \xi }\;2c_{\theta }(x,\eta )\int_{u}^{N}\;\left(\frac{1}{2}\frac{g\left(x,v_{1}\right)}{g(x,u)}\int_{R^{d}}\;(g(y,u)-g(x,u))q_{\theta }^{m}(x,dy,\eta )\right)dv_{1,}$$

$$A_{2}^{N}f\left(\xi \right)=f\left(\xi \right)\sum_{\left(x,u\right)\in \xi }\;2c_{\theta }\left(x,\eta \right)\int_{u}^{N}\left(\frac{1}{2}\int_{R^{d}}\left(\frac{g\left(y,v_{1}\right)+g\left(x,v_{1}\right)}{2}-1\right)q_{\theta }^{m}\left(x,dy,\eta \right)\right)dv_{1},$$

$$A_{3}^{N}f\left(\xi \right)=f\left(\xi \right)\sum_{\left(x,u\right)\in \xi }\left(c_{\theta }\left(x,\eta \right)u^{2}-b_{\theta }\left(x,\eta \right)u\right)\frac{\partial_{u}g\left(x,u\right)}{g\left(x,u\right)},$$

where $q^{m}$ was defined in equation (8.1), so that

$$A^{N}f\left(\xi \right)=A_{1}^{N}f\left(\xi \right)+A_{2}^{N}f\left(\xi \right)+A_{3}^{N}f\left(\xi \right).$$

We now integrate each piece against $\Gamma^{N}$. First note that by the product form of f,

$$\int f\left(\xi \right)\sum_{\left(x,u\right)\in \xi }\frac{\ell \left(x,u\right)}{g\left(x,u\right)}\Gamma^{N}\left(\eta ,d\xi \right)=F_{g}^{N}\left(\eta \right)\left\langle \frac{1}{N{\hat{g}}_{N}\left(x\right)}\int_{0}^{N}\;\ell \left(x,u\right)du,N\eta \left(dx\right)\right\rangle .$$

Therefore,

$$\int A_{1}^{N}f(\xi )\Gamma^{N}(\eta ,d\xi )\;\;=F_{g}^{N}(\eta )\left\langle \frac{c_{\theta }(x,\eta )}{{\hat{g}}_{N}(x)}\int_{0}^{N}\left\{\int_{u}^{N}\left(\frac{1}{2}g\left(x,v_{1}\right)\int_{R^{d}}\;(g(y,u)-g(x,u))q_{\theta }^{m}(x,dy,\eta )\right)dv_{1}\right\}du,\eta (dx)\right\rangle \;\;=F_{g}^{N}\left(\eta \right)\left\langle \frac{c_{\theta }\left(x,\eta \right)}{{\hat{g}}_{N}\left(x\right)}\left\{\int_{R^{d}}\left\{\int_{0}^{N}\;\int_{u}^{N}g\left(x,v_{1}\right)\left(g\left(y,u\right)-g\left(x,u\right)\right)dv_{1}du\right\}q_{\theta }^{m}\left(x,dy,\eta \right),\eta \left(dx\right)\right\rangle \right..$$

For the second generator, we have

$$\int A_{2}^{N}f(\xi )\Gamma^{N}(\eta ,d\xi )\;\;=F_{g}^{N}(\eta )\left\langle \frac{c_{\theta }(x,\eta )}{{\hat{g}}_{N}(x)}\int_{0}^{N}g(x,u)\left\{\int_{u}^{N}\left(\int_{R^{d}}\left(\frac{g\left(y,v_{1}\right)+g\left(x,v_{1}\right)}{2}-1\right)q_{\theta }^{m}(x,dy,\eta )\right)dv_{1}\right\},\eta (dx)\right\rangle \;\;=F_{g}^{N}(\eta )\left\langle \frac{c_{\theta }(x,\eta )}{{\hat{g}}_{N}(x)}\int_{R^{d}}\left\{\int_{0}^{N}\int_{u}^{N}g(x,u)\left(g\left(y,v_{1}\right)+g\left(x,v_{1}\right)-2\right)dv_{1}du\right\}q_{\theta }^{m}(x,dy,\eta ),\eta (dx)\right\rangle .$$

For the third generator we have that

$$\int A_{3}^{N}f(\xi )\Gamma^{N}(\eta ,d\xi )\;\;=F_{g}^{N}(\eta )\left\langle \frac{1}{{\hat{g}}_{N}(x)}\int_{0}^{N}\left(c_{\theta }(x,\eta )u^{2}-b_{\theta }(x,\eta )u\right)\partial_{u}g(x,u)du,\eta (dx)\right\rangle \;\;=F_{g}^{N}\left(\eta \right)\left\langle \frac{1}{{\hat{g}}_{N}\left(x\right)}\int_{0}^{N}\left(b_{\theta }\left(x,\eta \right)-2c_{\theta }\left(x,\eta \right)u\right)\left(g\left(x,u\right)-1\right)du,\eta \left(dx\right)\right\rangle .$$

Note that $2\int_{0}^{N}\int_{u}^{N}g\left(x,v_{1}\right)g(y,u)dv_{1}du=N^{2}{\hat{g}}_{N}(x){\hat{g}}_{N}(y)$, and so

$$\int_{0}^{N}\int_{u}^{N}g\left(x,v_{1}\right)(g(y,u)-g(x,u))dv_{1}du+\int_{0}^{N}\int_{u}^{N}g(x,u)\left(g\left(y,v_{1}\right)+g\left(x,v_{1}\right)-2\right)dv_{1}du\;\;=N^{2}\hat{g_{N}}\left(x\right)\left(\hat{g_{N}}\left(y\right)-2\right)+2\int_{0}^{N}ug\left(x,u\right)du.$$

Combining the last equations, and using the fact that $Nc_{\theta }(x,\eta )-b_{\theta }(x,\eta )=\theta \mu_{\theta }(x,\eta )$, we have

$$\int \left(A_{1}^{N}f(\xi )\right.\left.+A_{2}^{N}f(\xi )+A_{3}^{N}f(\xi )\right)\Gamma^{N}(\eta ,d\xi )\;\;=F_{g}^{N}(\eta )\left\langle c_{\theta }(x,\eta )N^{2}\int_{R^{d}}\;\left({\hat{g}}_{N}(y)-2\right)q^{m}(x,dy,\eta )+\frac{1}{{\hat{g}}_{N}(x)}c_{\theta }(x,\eta )\int_{0}^{N}2udu\right.\;\;\left.+\frac{1}{{\hat{g}}_{N}(x)}b_{\theta }(x,\eta )\int_{0}^{N}(g(x,u)-1)du,\eta (dx)\right\rangle \;\;=F_{g}^{N}(\eta )\left\langle c_{\theta }(x,\eta )N^{2}\int_{R^{d}}\left({\hat{g}}_{N}(y)-1\right)q^{m}(x,dy,\eta )+N^{2}c_{\theta }(x,\eta )\left(\frac{1}{{\hat{g}}_{N}(x)}-1\right)\right.\;\;\left.+Nb_{\theta }(x,\eta )\left(1-\frac{1}{{\hat{g}}_{N}(x)}\right),\eta (dx)\right\rangle \;\;=F_{g}^{N}(\eta )\left\langle Nc_{\theta }(x,\eta )\int_{R^{d}}\left({\hat{g}}_{N}(y)-1\right)q^{m}(x,dy,\eta )+\theta \mu_{\theta }(x,\eta )\left(\frac{1}{{\hat{g}}_{N}(x)}-1\right),\eta (dx)\right\rangle .$$

This matches equation (A.3), as desired, because $Nc_{\theta }(x,\eta )q_{m}(x,dy,\eta )=\theta \gamma (x,\eta )q_{\theta }(x,dy)$. □

Before proving Lemma A.3, we recall an important equality for conditionally Poisson point processes (Kurtz and Rodrigues [2011] Lemma A.3).

#### Lemma A.4

*If* $\xi =\sum_{i}\delta_{Z_{i}}$ is a Poisson random measure with mean measure ν, then for $\ell \in L^{1}(\nu )$ and g≥0 with $log\;g\in L^{1}(\nu )$,

(A.7)
$$E\left[\sum_{j}\ell \left(Z_{j}\right)\prod_{i}g\left(Z_{i}\right)\right]=\int \ell gd\nu e^{\int \left(g-1\right)d\nu }.$$

#### Proof [ of Lemma A.3]

By Lemma A.4,

$$\int f(\xi )\sum_{(x,u)\in \xi }\frac{\ell (x,u)}{g(x,u)}\Gamma (\eta ,\xi )=F_{g}(\eta )\langle \int_{0}^{\infty }\ell (x,u)du,\eta (dx)\rangle .$$

Comparing this to the definition of A (equation (5.11)), we see that

$$\int Af\left(\xi \right)\Gamma \left(\eta ,d\xi \right)=F_{g}\left(\eta \right)\left\langle \int_{0}^{\infty }\left(\ell_{1}\left(x,u\right)+\ell_{2}\left(x,u\right)+\ell_{3}\left(x,u\right)\right)du,\eta \left(dx\right)\right\rangle ,$$

where

$$\ell_{1}(x,u)\;=\gamma (x,\eta )(ℬ(g(\cdot ,u)r(\cdot ,\eta ))(x)-g(x,u)ℬr(x,\eta ))\;\;=\gamma (x,\eta )(ℬ((g(\cdot ,u)-1)r(\cdot ,\eta ))(x)-(g(x,u)-1)ℬr(x,\eta ))$$

and

$$\ell_{2}(x,u)=2g(x,u)\alpha \gamma (x,\eta )r(x,\eta )\int_{u}^{\infty }(g(x,v)-1)dv.$$

and

$$\ell_{3}(x,u)=\left(\alpha \gamma (x,\eta )r(x,\eta )u^{2}-\{\gamma (x,\eta )ℬr(x,\eta )+F(x,\eta )\}u\right)\partial_{u}g(x,u)$$

First note that since ℬ acts on space, it commutes with the integral over levels, and so

$$\int_{0}^{\infty }\ell_{1}\left(x,u\right)du=\gamma \left(x,\eta \right)\left(ℬ\left(\tilde{g}\left(\cdot \right)r\left(\cdot ,\eta \right)\right)\left(x\right)-\tilde{g}\left(x\right)ℬr\left(x,\eta \right)\right),$$

since $\tilde{g}(x)=\int_{0}^{\infty }\;(g(x,u)-1)du$. Next,

$$\int_{0}^{\infty }\ell_{2}\left(x,u\right)du=\alpha \gamma \left(x,\eta \right)r\left(x,\eta \right)2\int_{0}^{\infty }g\left(x,u\right)\int_{u}^{\infty }\left(g\left(x,v\right)-1\right)dvdu.$$

Finally, integrating by parts,

$$\int_{0}^{\infty }\;\ell_{3}(x,u)du=-\alpha \gamma (x,\eta )r(x,\eta )\int_{0}^{\infty }\;2u(g(x,u)-1)du\;\;+\{\gamma (x,\eta )ℬr(x,\eta )+F(x,\eta )\}\tilde{g}(x)$$

Now, note that

$$\int_{0}^{\infty }g(x,u)\int_{u}^{\infty }(g(x,v)-1)dvdu-\int_{0}^{\infty }u(g(x,u)-1)du\;\;=\int_{0}^{\infty }g(x,u)\int_{u}^{\infty }(g(x,v)-1)dvdu-\int_{0}^{\infty }\int_{v}^{\infty }(g(x,u)-1)dudv\;\;=\int_{0}^{\infty }(g(x,u)-1)\int_{u}^{\infty }(g(x,v)-1)dvdu\;\;=\tilde{g}(x{)}^{2}/2.$$

Adding these together, we get that

$$\int_{0}^{\infty }\left(\ell_{1}(x,u)+\ell_{2}(x,u)+\ell_{3}(x,u)\right)du\;\;=\gamma (x,\eta )ℬ(\tilde{g}(\cdot )r(\cdot ,\eta ))(x)+F(x,\eta )\tilde{g}(x)+\alpha \gamma (x,\eta )r(x,\eta )\tilde{g}(x{)}^{2},$$

which agrees with (A.5), as desired. □

## B Technical Lemmas

### B.1 Constraints on kernel widths

#### Lemma B.1

Suppose the first three conditions of Assumptions 2.8 hold, and furthermore the kernels $\rho_{r}=p_{\epsilon_{r}^{2}}$ and $\rho_{\gamma }=p_{\epsilon_{\gamma }^{2}}$ are each Gaussian with standard deviations $\epsilon_{r}$ and $\epsilon_{\gamma }$ respectively. Let $\lambda ={sup}_{x}\;{sup}_{y:∥y∥=1}\;y^{T}C(x)y$ be the largest eigenvalue of C(x) across all x. If $\epsilon_{r}^{2}+\frac{2\lambda }{\theta }<\epsilon_{\gamma }^{2}$, then there is a C<∞ such that for all $x\in R^{d},\;\eta \in {ℳ}_{F}\left(R^{d}\right)$,

(B.1)
$$\left|\theta \int_{R^{d}}\left(\rho_{r}*\eta (y)-\rho_{r}*\eta (x)\right)q_{\theta }(x,dy)\right|\leq C\rho_{\gamma }*\eta (x)$$

and

(B.2)
$$\theta \int_{R^{d}}{\left(\rho_{r}*\eta (y)-\rho_{r}*\eta (x)\right)}^{2}q_{\theta }(x,dy)\leq C{\left(\rho_{\gamma }*\eta (x)\right)}^{2}.$$

Note that the right hand side of each is the average density over a wider region (since $\epsilon_{\gamma }>\epsilon_{r}$). The key assumption here is that the spatial scale over which local density affects birth rate is larger than the scale over which it affects establishment. In the simple case of $\vec{b}=0$ and $C=\sigma^{2}I$, the condition is simply that $\epsilon_{r}^{2}+2\sigma^{2}/\theta <\epsilon_{\gamma }^{2}$. This gives a yet more concrete situation in which Condition 2 of Lemma 2.9 holds.

#### Proof [of Lemma B.1]

First we prove (B.1). Recall that $\rho_{r}*\eta (x)=\int p_{\epsilon_{r}^{2}}(x-w)\eta (dw)$, where $p_{t}$ is the density of a Gaussian with mean 0 and variance t, so that applying Fubini, (B.1) is

$$\left|\int_{R^{d}}\theta \int_{R^{d}}\left(p_{\epsilon_{r}^{2}}(y-w)-p_{\epsilon_{r}^{2}}(x-w)\right)q_{\theta }(x,dy)\eta (dw)\right|.$$

Write $p_{s,x}(\cdot )$ for the density of a Gaussian with mean $s\vec{b}(x)$ and covariance $\epsilon_{r}^{2}I+sC(x)$, so that $\int p_{\epsilon_{r}^{2}}(y-w)q_{\theta }(x,dy)=p_{1/\theta ,x}(w-x)$. It therefore suffices to show that for all x and $w\in R^{d}$, there exists K such that

$$\left|\theta \int_{R^{d}}\left(p_{\epsilon_{r}^{2}}(y-w)-p_{\epsilon_{r}^{2}}(x-w)\right)q_{\theta }(x,dy)\right|=\theta \left|p_{1/\theta ,x}(w-x)-p_{0,x}(w-x)\right|\leq Kp_{\epsilon_{\gamma }^{2}}(w-x).$$

However, $\theta \left(p_{1/\theta ,x}(z)-p_{0,x}(z)\right)=\partial_{s}p_{s,x}(z)$ for some 0≤s≤1/θ. Write $\Gamma (s,x)=\epsilon_{r}^{2}I+sC(x)$, so that

$$p_{s,x}(z)=\frac{1}{(2\pi |\Gamma (s,x)|{)}^{d/2}}exp\left(-\frac{1}{2}(z-s\vec{b}(x){)}^{T}\Gamma (s,x{)}^{-1}(z-s\vec{b}(x))\right),$$

and note that if $\lambda_{i}$ are the eigenvalues of C(x) then $|\Gamma (s,x)|=\prod_{i}\left(\epsilon_{r}^{2}+s\lambda_{i}\right)$, and $\partial_{s}|\Gamma (s,x)|=\sum_{i}\lambda_{i}|\Gamma (s,x)|/\left(\epsilon_{r}^{2}+s\lambda_{i}\right)$. Therefore,

$$\partial_{s}p_{s,x}(z)=\left(\vec{b}(x{)}^{T}\Gamma (s,x{)}^{-1}(z-s\vec{b}(x))+(z-s\vec{b}(x){)}^{T}\Gamma (s,x{)}^{-1}C(x)\Gamma (s,x{)}^{-1}(z-s\vec{b}(x))\right.\;\;\left.-\sum_{i}\;\frac{\lambda_{i}}{\epsilon_{r}^{2}+s\lambda_{i}}\right)p_{s,x}\left(z\right),$$

where $z^{T}$ is the transpose of z. This implies that

$$\frac{\theta \int_{R^{d}}\left(p_{\epsilon_{r}^{2}}(y-w)-p_{\epsilon_{r}^{2}}(x-w)\right)q_{\theta }(x,dy)}{p_{\epsilon_{\gamma }^{2}}(x-w).}=h\left(x-w\right)e^{k\left(x-w\right)},$$

where h(z) and k(z) are quadratic polynomials in z whose coefficients depend on s and x but are uniformly bounded, and

$$k(z)=\frac{1}{2\epsilon_{\gamma }^{2}}∥z{∥}^{2}-\frac{1}{2}(z-s\vec{b}(x){)}^{T}\Gamma (s,x{)}^{-1}\left(z-s\vec{b}\left(x\right)\right),$$

Since ${inf}_{z}\;z^{T}\Gamma (s,x{)}^{-1}z/∥z{∥}^{2}=1/\left(s\lambda (x)+\epsilon_{r}^{2}\right)$, where $\lambda (x)=supz^{T}C(x)z/∥z{∥}^{2}$ is the largest eigenvalue of C(x), this is negative for all z outside a bounded region, and so equation (B.1) follows from the assumption that $\epsilon_{r}^{2}+2\;{sup}_{x}\;\lambda (x)/\theta <\epsilon_{\gamma }^{2}$. (Note that we do not yet need the factor of 2.)

Next we prove equation (B.2), in a similar way. Again applying Fubini,

$$\theta \int_{R^{d}}{\left(\rho_{r}*\eta (y)-\rho_{r}*\eta (x)\right)}^{2}q_{\theta }(x,dy)\;\;=\;\int_{R^{d}}\int_{R^{d}}\theta \int_{R^{d}}\left(p_{\epsilon_{r}^{2}}(y-w)-p_{\epsilon_{r}^{2}}(x-w)\right)\left(p_{\epsilon_{r}^{2}}(y-v)-p_{\epsilon_{r}^{2}}(x-v)\right)q_{\theta }(x,dy)\eta (dv)\eta (dw),$$

and so as before, equation (B.2) will follow if the integrand is bounded by $Kp_{\gamma }(x-w)p_{\gamma }(x-v)$. Now, let $Y_{1},\;Y_{2}$, and Z be independent d-dimensional Gaussians with mean zero, where $Y_{1}$ and $Y_{2}$ have covariance $\epsilon_{r}^{2}I$, and Z has covariance C(x). Write $p_{s,t,x}(\cdot ,\cdot )$ for the joint density of $Y_{1}+\sqrt{s}Z+s\vec{b}(x)$ and $Y_{2}+\sqrt{t}Z+t\vec{b}(x)$. Then, observe that

$$\theta \int_{R^{d}}\left(p_{\epsilon_{r}^{2}}(y-w)-p_{\epsilon_{r}^{2}}(x-w)\right)\left(p_{\epsilon_{r}^{2}}(y-v)-p_{\epsilon_{r}^{2}}(x-v)\right)q_{\theta }(x,dy)\;\;=\theta \left(p_{1/\theta ,1/\theta ,x}(x-w,x-v)-p_{0,1/\theta ,x}(x-w,x-v)\right.\;\;\left.\;-p_{1/\theta ,0,x}(x-w,x-v)+p_{0,0,x}(x-w,x-v)\right)\;\;=\partial_{s}p_{s,1/\theta ,x}(x-w,x-v)-\partial_{t}p_{0,t,x}(x-w,x-v),$$

for some 0≤s,t≤1/θ. As before,

$$\frac{\theta \int_{R^{d}}\left(p_{\epsilon_{r}^{2}}(y-w)-p_{\epsilon_{r}^{2}}(x-w)\right)\left(p_{\epsilon_{r}^{2}}(y-v)-p_{\epsilon_{r}^{2}}(x-v)\right)q_{\theta }(x,dy)}{p_{\epsilon_{\gamma }^{2}}(x-w)p_{\epsilon_{\gamma }^{2}}(x-v)}=h(x-w,x-v)e^{k(x-w,x-v)},$$

where $h\left(z_{1},z_{2}\right)$ is a polynomial with uniformly bounded coefficients and

$$k\left(z_{1},z_{2}\right)=\left({∥z_{1}∥}^{2}+{∥z_{2}∥}^{2}\right)/\left(2\epsilon_{\gamma }^{2}\right)-\frac{1}{2}{\left[z_{1},z_{2}\right]}^{T}\Gamma (s,t,x{)}^{-1}\left[z_{1},z_{2}\right],$$

where $\left[z_{1},z_{2}\right]$ is the $R^{2d}$ vector formed by concatenating $z_{1}$ and $z_{2}$, and Γ(s,t,x) is the block matrix

$$\Gamma \left(s,t,x\right)=\left[\begin{array}{cc} \epsilon_{r}^{2}I+sC\left(x\right) & \sqrt{st}C\left(x\right)\\ \sqrt{st}C\left(x\right) & \epsilon_{r}^{2}I+tC\left(x\right) \end{array}\right].$$

If C(x)u=au for some a∈R, then $\left[u\sqrt{s},u\sqrt{t}\right]$ is an eigenvector of Γ(s,t,x) with eigenvalue $\epsilon_{r}^{2}+(s+t)a$, and $\left[u\sqrt{t},-u\sqrt{s}\right]$ is an eigenvector of Γ(s,t,x) with eigenvalue 0. This implies the largest eigenvalue of Γ(s,t,x) is equal to $\epsilon_{r}^{2}+(s+t)\lambda (x)$, where λ(x) is again the largest eigenvalue of C(x). Therefore, if s+t≤2/θ,

$$\left({∥z_{1}∥}^{2}\right.\left.+{∥z_{2}∥}^{2}\right)/\epsilon_{\gamma }^{2}-{\left[z_{1},z_{2}\right]}^{T}\Gamma (s,t,x{)}^{-1}\left[z_{1},z_{2}\right]\;\;\;\leq \left({∥z_{1}∥}^{2}+{∥z_{2}∥}^{2}\right)\left(\frac{1}{\epsilon_{\gamma }^{2}}-\frac{1}{\epsilon_{r}^{2}+\frac{2\lambda \left(x\right)}{\theta }}\right),$$

which is negative by assumption. Therefore, there is a K such that

$$\frac{\left|\partial_{s}p_{s,1/\theta ,x}(x-w,x-v)-\partial_{t}p_{0,t,x}(x-w,x-v)\right|}{p_{\epsilon_{\gamma }^{2}}(x-w)p_{\epsilon_{\gamma }^{2}}(x-v)}\leq K$$

for all θ>1 and all x, v, and $w\in R^{d}$, proving equation (B.2) and hence the lemma. □

### B.2 Tightness of processes

Here we record, for completeness, the fact used above that tightness for a family of processes, if determined by the Aldous-Rebolledo criterion, extends to sums and products of those processes. We first record for reference one version of the Aldous-Rebolledo criteria for tightness of a sequence real-valued processes (as it appears in Etheridge [2000]):

#### Theorem B.2 (Rebolledo [1980])

Let ${\left\{Y^{\left(n\right)}\right\}}_{n\geq 1}$ be a sequence of real-valued processes with càdlàg paths. Suppose that the following conditions are satisfied.

1. For each fixed t∈[0,T], ${\left\{Y_{t}^{\left(n\right)}\right\}}_{n\geq 1}$ is tight.
2. Given a sequence of stopping times $\tau_{n}$, bounded by T, for each ϵ>0 there exists δ>0 and $n_{0}$ such that
  $$\underset{n\geq n_{0}}{sup}\;\underset{\theta \in \left[0,min\left(\delta ,T-\tau_{n}\right)\right]}{sup}\;P\left\{\left|Y_{\tau_{n}+\theta }^{\left(n\right)}-Y_{\tau_{n}}^{\left(n\right)}\right|>\epsilon \right\}\leq \epsilon .$$
  Then the sequence ${\left\{{\left(Y_{t}^{\left(n\right)}\right)}_{t=0}^{T}\right\}}_{n\geq 1}$ is tight.

#### Lemma B.3

Let ${\left\{X^{\left(n\right)}\right\}}_{n\geq 1}$ and ${\left\{Y^{\left(n\right)}\right\}}_{n\geq 1}$ be sequences of jointly defined real-valued processes with càdlàg paths satisfying the conditions of Theorem B.2. Then ${\left\{X^{\left(n\right)}Y^{\left(n\right)}\right\}}_{n\geq 1}$ and ${\left\{X^{\left(n\right)}+Y^{\left(n\right)}\right\}}_{n\geq 1}$ also satisfy the conditions of Theorem B.2.

By “jointly defined” we mean that $X^{\left(n\right)}$ and $Y^{\left(n\right)}$ are defined on the same probability space, so that the products and sums make sense.

#### Proof [of Lemma B.3]

The proof for $X^{\left(n\right)}+Y^{\left(n\right)}$ is similar to but more straightforward than for $X^{\left(n\right)}Y^{\left(n\right)}$, so on only prove the Lemma for the latter. First, note that for any ϵ>0, by tightness of $X_{t}^{\left(n\right)}$ and $Y_{t}^{\left(n\right)}$ there is a K such that $P\left\{X_{t}^{\left(n\right)}>\sqrt{K}\right\}$ and $P\left\{Y_{t}^{\left(n\right)}>\sqrt{K}\right\}$ are both less than ϵ/2, and hence

$$P\left\{X_{t}^{\left(n\right)}Y_{t}^{\left(n\right)}>K\right\}\leq P\left\{X_{t}^{\left(n\right)}>\sqrt{K}\right\}+P\left\{Y_{t}^{\left(n\right)}>\sqrt{K}\right\}\leq \epsilon .$$

Therefore, $X_{t}^{\left(n\right)}Y_{t}^{\left(n\right)}$ is tight.

Next, note that for $0\leq \tau_{n}\leq T,$

$$\underset{0\leq \theta \leq min\left(\delta ,T-\tau_{n}\right)}{sup}\;\left|X_{\tau_{n}+\theta }^{\left(n\right)}Y_{\tau_{n}+\theta }^{\left(n\right)}-X_{\tau_{n}}^{\left(n\right)}Y_{\tau_{n}}^{\left(n\right)}\right|\;\;\leq \underset{0\leq \theta \leq min\left(\delta ,T-\tau_{n}\right)}{sup}\;\left|X_{\tau_{n}+\theta }^{\left(n\right)}\right|\left|Y_{\tau_{n}+\theta }^{\left(n\right)}-Y_{\tau_{n}}^{\left(n\right)}\right|+\left|X_{\tau_{n}+\theta }^{\left(n\right)}-X_{\tau_{n}}^{\left(n\right)}\right|\left|Y_{\tau_{n}}^{\left(n\right)}\right|\;\;\leq \underset{0\leq t\leq T}{sup}\;\left|X_{t}^{\left(n\right)}\right|\underset{0\leq \theta \leq min\left(\delta ,T-\tau_{n}\right)}{sup}\;\left|Y_{\tau_{n}+\theta }^{\left(n\right)}-Y_{\tau_{n}}^{\left(n\right)}\right|+\underset{0\leq \theta \leq min\left(\delta ,T-\tau_{n}\right)}{sup}\;\left|X_{\tau_{n}+\theta }^{\left(n\right)}-X_{\tau_{n}}^{\left(n\right)}\right|\underset{0\leq t\leq T}{sup}\;\left|Y_{t}^{\left(n\right)}\right|,$$

so that for any C,

(B.3)
$$P\left\{\underset{0\leq \theta \leq min\left(\delta ,T-\tau_{n}\right)}{sup}\;\left|X_{\tau_{n}+\theta }^{\left(n\right)}Y_{\tau_{n}+\theta }^{\left(n\right)}-X_{\tau_{n}}^{\left(n\right)}Y_{\tau_{n}}^{\left(n\right)}\right|>\epsilon \right\}\;\;\leq P\left\{\underset{0\leq t\leq T}{sup}\;\left|X_{t}^{\left(n\right)}\right|>C\right\}+P\left\{\underset{0\leq \theta \leq min\left(\delta ,T-\tau_{n}\right)}{sup}\;\left|Y_{\tau_{n}+\theta }^{\left(n\right)}-Y_{\tau_{n}}^{\left(n\right)}\right|>\epsilon /C\right\}\;\;+P\left\{\underset{0\leq \theta \leq min\left(\delta ,T-\tau_{n}\right)}{sup}\;\left|X_{\tau_{n}+\theta }^{\left(n\right)}-X_{\tau_{n}}^{\left(n\right)}\right|>\epsilon /C\right\}+P\left\{\underset{0\leq t\leq T}{sup}\;\left|Y_{t}^{\left(n\right)}\right|>C\right\}$$

Now, since ${max}_{0\leq t\leq T}\;X_{t}^{\left(n\right)}$ is tight (and likewise for Y) (see, e.g., Remark 3.7.3 in Ethier and Kurtz [1986]), we may choose a C≥4 for which

$$P\left\{\underset{0\leq t\leq T}{sup}\;\left|X_{t}^{\left(n\right)}\right|>C\right\}\leq \frac{\epsilon }{4}.$$

Similarly, by assumption we can choose a δ for which

$$P\left\{\underset{0\leq \theta \leq min\left(\delta ,T-\tau_{n}\right)}{sup}\;\left|X_{\tau_{n}+\theta }^{\left(n\right)}-X_{\tau_{n}}^{\left(n\right)}\right|>\epsilon /C\right\}\leq \frac{\epsilon }{C}.$$

If we choose C and δ that do this for both $X^{\left(n\right)}$ and $Y^{\left(n\right)}$, then each of the terms in equation (B.3) are bounded by ϵ/4, and condition (2) is satisfied for the product process. □
